# The distribution of manta rays in the western North Atlantic Ocean off the eastern United States

**DOI:** 10.1038/s41598-022-10482-8

**Published:** 2022-04-21

**Authors:** Nicholas A. Farmer, Lance P. Garrison, Calusa Horn, Margaret Miller, Timothy Gowan, Robert D. Kenney, Michelle Vukovich, Julia Robinson Willmott, Jessica Pate, D. Harry Webb, Timothy J. Mullican, Joshua D. Stewart, Kim Bassos-Hull, Christian Jones, Delaney Adams, Nicole A. Pelletier, Jordan Waldron, Stephen Kajiura

**Affiliations:** 1 NOAA/National Marine Fisheries Service, Southeast Regional Office, 263 13th Ave S., St. Petersburg, FL 33701 USA; 2 NOAA/National Marine Fisheries Service, Southeast Fisheries Science Center, 75 Virginia Beach Dr., Miami, FL 33149 USA; 3 NOAA/National Marine Fisheries Service, Office of Protected Resources, 1315 East-West Highway, Silver Spring, MD 20910 USA; 4 Florida Fish and Wildlife Research Institute, 100 8th Ave SE, St. Petersburg, FL 33701 USA; 5 Graduate School of Oceanography, University of Rhode Island, Bay Campus Box 40, 215 South Ferry Rd., Narragansett, RI 02882 USA; 6 Normandeau Associates Inc., 4581 NW 6th Street, Suite H, Gainesville, FL 32609 USA; 7 Marine Megafauna Foundation, 7750 Okeechobee Blvd, Ste 4-3038, West Palm Beach, FL 33411 USA; 8 Georgia Aquarium, 225 Baker St. NW, Atlanta, GA 30313 USA; 9 The Manta Trust, Catemwood House, Corscombe, Dorchester, Dorset, DT2 0NT UK; 10 NOAA/National Marine Fisheries Service, Southwest Fisheries Science Center, La Jolla Shores Drive, La Jolla, CA 92037 USA; 11 Sharks and Rays Conservation Research Program, Mote Marine Laboratory, 1600 Ken Thompson Pkwy, Sarasota, FL 34236 USA; 12 NOAA/National Marine Fisheries Service, Southeast Fisheries Science Center, 3209 Frederic Street, Pascagoula, MS 39567-4112 USA; 13 Western Washington University, 516 High Street, Bellingham, WA 98225 USA; 14 Florida Atlantic University, 777 Glades Road, Boca Raton, FL 33431 USA

**Keywords:** Marine biology, Conservation biology

## Abstract

In 2018, the giant manta ray was listed as threatened under the U.S. Endangered Species Act. We integrated decades of sightings and survey effort data from multiple sources in a comprehensive species distribution modeling (SDM) framework to evaluate the distribution of giant manta rays off the eastern United States, including the Gulf of Mexico. Manta rays were most commonly detected at productive nearshore and shelf-edge upwelling zones at surface thermal frontal boundaries within a temperature range of approximately 20–30 °C. SDMs predicted highest nearshore occurrence off northeastern Florida during April, with the distribution extending northward along the shelf-edge as temperatures warm, leading to higher occurrences north of Cape Hatteras, North Carolina from June to October, and then south of Savannah, Georgia from November to March as temperatures cool. In the Gulf of Mexico, the highest nearshore occurrence was predicted around the Mississippi River delta from April to June and again from October to November. SDM predictions will allow resource managers to more effectively protect manta rays from fisheries bycatch, boat strikes, oil and gas activities, contaminants and pollutants, and other threats.

## Introduction

Manta rays are filter-feeding rays in the family Mobulidae, characterized by a terminal mouth, diamond-shaped bodies with wing-like pectoral fins, and long cephalic fins^1^. The taxonomic history of the genus *Manta* is complex^1–8^, with more recent studies supporting a split of the *Manta* genus into two species: *M. birostris* and *M. alfredi*^9^, and synonymizing the genus *Manta* with the genus *Mobula*^10^. Of the two manta species, only giant manta rays (*Mobula birostris*) occur in the western North Atlantic Ocean^11^. In 2018, giant manta rays were listed as a threatened species (under the taxonomic designation of *Manta birostris*) by the U.S. National Oceanic and Atmospheric Administration (NOAA) under the U.S. Endangered Species Act (ESA) due to significant declines in abundance from overutilization in the Indo-Pacific and eastern Pacific portion of its range, exacerbated by a lack of effective management measures to control this threat and the species’ inherent vulnerability to depletion due to its slow growth, late maturation, and low reproductive output^11^.

Effective conservation and management of highly mobile marine species requires an understanding of the environmental drivers of their spatio-temporal distribution. NOAA did not designate critical habitat under the ESA for giant manta rays due to a lack of available data to identify physical or biological features essential to their conservation within areas under U.S. jurisdiction^12^. Significant data gaps were identified regarding giant manta ray movements, foraging areas, aggregation sites and nursery grounds. Giant manta rays appear to conduct seasonal migrations following prey abundance^13–15^, with prey including planktonic and micronektonic organisms such as euphausiids, copepods, mysids, decapod larvae and shrimp, and fish spawn^16–19^. Studies from several locations around the world have documented seasonal sighting patterns associated with movements of prey, current circulation and tidal patterns, seasonal upwelling, seawater temperature, and possibly mating behavior^1,20,21^.

**Figure 1:**
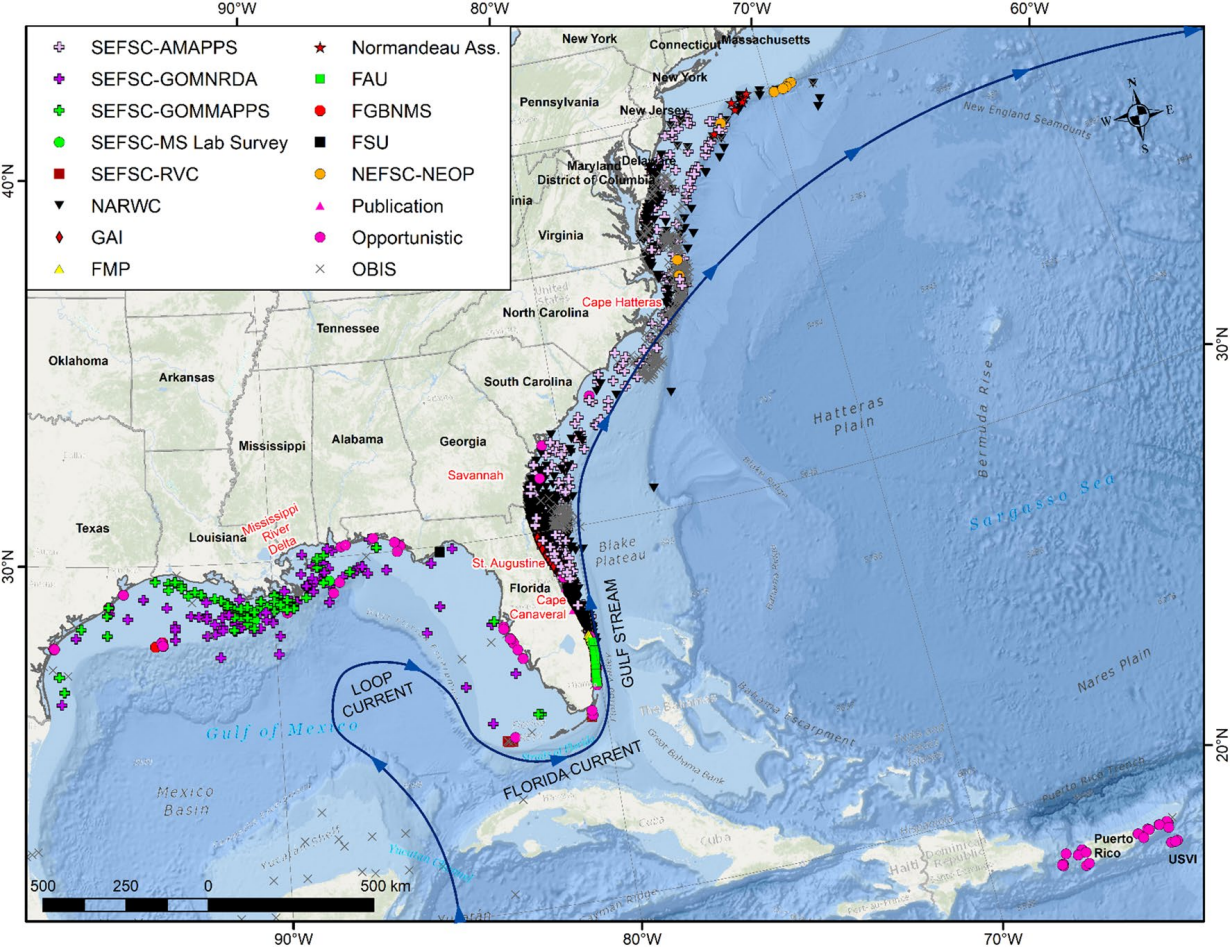
Reported sightings of manta rays (1925–2020) relative to regional landmarks and ocean currents. Sightings from Southeast Fisheries Science Center (SEFSC) Atlantic Marine Assessment Program for Protected Species (AMAPPS), Gulf of Mexico Marine Assessment Program for Protected Species (GOMAPPS), Gulf of Mexico Natural Resource Damage Assessment (GOMNRDA) aerial surveys, Mississippi Lab pelagic longline surveys (MS Lab Survey), and Reef Visual Census (RVC) SCUBA-based survey; Florida Atlantic University (FAU) Kajiura Lab aerial elasmobranch surveys, Flower Garden Banks National Marine Sanctuary (FGBNMS) staff sightings, boat-based and aerial surveys by trained Florida Manta Project (FMP) staff, Florida State University (FSU) Grubbs Lab elasmobranch gillnet surveys, Georgia Aquarium (GAI) aerial surveys, Ocean Biodiversity Information System (OBIS) open-access data, North Atlantic Right Whale Consortium (NARWC) ship-based and aerial surveys, Northeast Fisheries Science Center (NEFSC) Northeast Observer Program (NEOP) trawl encounters, APEM and Normandeau Associates Aerial Digital Baseline Survey of Marine Wildlife in Support of Offshore Wind Energy for New York State Energy Research and Development Authority (NYSERDA) and U.S. Bureau of Ocean Energy Management, and verified opportunistic sightings reported to the authors or pulled from social media and news reports. Basemap used with permission from ESRI Ocean Basemap and its partners, showing marine water body names, undersea feature names, and derived depth values in meters. Map generated in ArcMap 10.8.1 (https://support.esri.com/en/products/desktop/arcgis-desktop/arcmap/10-8).

The eastern United States (EUS), defined here as the U.S. exclusive economic zone from Maine to Texas, is characterized by three major current systems that comprise segments of the North Atlantic Gyre: (1) the Loop Current, (2) the Florida Current, and (3) the Gulf Stream. The warm waters of the Loop Current travel up from the Caribbean, between Cuba and Mexico’s Yucatan Peninsula, and into the Gulf of Mexico to form the Gulf Loop Current^22^ (Fig. 1). The Gulf Loop Current is variable; sometimes barely entering the Gulf of Mexico before traveling to the Atlantic, and at other times traveling nearly to the coast of Louisiana before curving east and south along Florida's west coast. The current is also known as the Florida Current as it flows through the Florida Strait, into the Gulf Stream. The Gulf Stream is an intense, warm ocean current in the western North Atlantic Ocean^23^. It moves quickly north along the coast of Florida at an average speed of 6.4 km/h and then turns eastward off Cape Hatteras, North Carolina, and slows to 1.6 km/h as it flows northeast across the Atlantic. Regular manta ray sightings have been reported in putative nursery areas at Flower Garden Banks National Marine Sanctuary (FGBNMS) in the Gulf of Mexico^24,25^ and along Florida’s eastern coast^26^ (Webb et al. unpublished data). A putative species or subspecies of the giant manta ray has been suggested (referred to as *M. cf. birostris*^9,27^) to occur off southeastern Florida^26^, FGBNMS^25^, the Yucatan peninsula^28^, Brazil^29^, and the Caribbean (N. Pelletier, unpublished data). Given the lack of easily recognized visually distinguishing characteristics, our analysis of manta ray distributions in the EUS should be considered inclusive of *M. cf. birostris* and we refer to this aggregate as ‘manta rays’ throughout.

The spatio-temporal distribution of manta rays in the EUS is poorly understood. A recent study evaluated habitat suitability and regional hotspots for manta rays in the Gulf of Mexico and Caribbean^30^. This ecological niche model^30^ was developed using presence-only data for putative manta ray sightings, and found that distributions were best predicted by chlorophyll-a and bathymetric slope, with minimal predictive input from sea surface temperature (SST). The authors noted that the majority of sightings were from coastal waters, potentially due to increased but unquantified survey effort^30^. In our study, we integrate characterized manta ray sightings and survey effort data across numerous sources from decades of sampling across a broad geographic range, then evaluate these distance-weighted sampling data in a comprehensive species distribution modeling (SDM) framework to evaluate environmental drivers of manta ray occurrence. Finally, we assess the predictive utility of our SDM outputs through comparison with independent confirmed sightings data.

Although manta rays are not targeted by fisheries in the United States, they are adversely affected by commercial and recreational fishing bycatch, boat strikes, oil and gas activities, contaminants and pollutants, military activities, and climate change^26,31^. A more comprehensive understanding of the distribution of the species will allow managers to more effectively protect manta rays from these threats. Understanding the environmental drivers of their distribution will allow researchers to more efficiently locate these animals for scientific study and potentially predict changes in their distribution under climate change scenarios. SDMs also provide managers a better understanding of the factors influencing giant manta ray movements and habitat use, facilitating more accurate assessment of the likelihood and duration of exposure to potentially harmful anthropogenic activities (i.e., oil and gas exploration, construction activities, dredging, etc.). Finally, understanding the spatiotemporal distribution of manta rays enables more effective timing and coordination of conservation efforts (e.g., outreach to reduce recreational fisheries bycatch and vessel collisions).

## Methods

### Sightings data

Few dedicated surveys for manta rays exist in the EUS; however, due to their large size and distinct appearance, they are often observed and recorded during visual aerial surveys that target marine mammals and sea turtles. To better characterize the distribution of manta rays in EUS waters, we assembled a comprehensive geographic information system database of manta sightings from peer-reviewed literature, survey databases, gray literature reports, and opportunistic sources (e.g., social media, press reports, personal communications). A comprehensive internet search for the terms “giant manta rays”, “rays”, or “manta rays” through Google and social media (i.e., Instagram, Facebook, YouTube, and Twitter) revealed many photos and videos of manta sightings from news outlets and citizen scientists. Opportunistic and social media sightings are biased towards locations with higher concentrations of people. A snowball technique was also used during social media searches; after a post meeting the search criteria was discovered, the comments were reviewed to find more posts. Publishers of sightings content were then contacted and asked about manta size, location, and date of the sighting. Additional opportunistic sightings were received through NOAA’s reporting email: manta.ray@noaa.gov. Only sightings confirmed through photos, videos, or informal interviews were included in the database generated from social media, news, grey literature (N. Pelletier and D. Adams, unpublished data), and sighting reports (C. Horn, unpublished data). All methods were carried out with informed consent from all subjects in accordance with relevant guidelines and regulations as approved by NOAA Fisheries. Presence-only records from these searches and reports are biased towards more heavily populated areas due to their unquantifiable search effort, but provide a useful source for independent validation of SDMs generated from the surveys described below.

#### Southeast fisheries science center (SEFSC) aerial surveys

Aerial line-transect surveys were conducted along the U.S. Gulf of Mexico and Atlantic coast between Texas and New Jersey by the National Marine Fisheries Service SEFSC between 2010 and 2019. These aerial surveys were primarily designed to estimate the abundance of marine mammals and sea turtles in continental shelf waters. Surveys were conducted once per season in the Gulf of Mexico during 2011–2012 as part of the Natural Resources Damage Assessment associated with the Deepwater Horizon oil spill (SEFSC-GOMNRDA^32^), and three surveys of the Gulf of Mexico were conducted during 2017–2018 as part of the Gulf of Mexico Marine Assessment Program for Protected Species (SEFSC-GoMMAPPS^33^) and covered the continental shelf and the inner continental slope from Texas to southwest Florida. Along the U.S. Atlantic coast, similar surveys were flown covering all four seasons between 2010 and 2019. These surveys were conducted as part of the Atlantic Marine Assessment Program for Protected Species (SEFSC-AMAPPS^34,35^) and covered the continental shelf and the inner continental slope from southeast Florida to New Jersey.

All SEFSC surveys were conducted aboard a DeHavilland DHC-6 Twin Otter and were flown at an altitude of 182 m and a target airspeed of 185 km/h. Surveys were typically flown during favorable sighting conditions at Beaufort sea states ≤ 4 (surface winds < 30 km/h). Two independent teams of visual observers searched for marine mammals and sea turtles from directly beneath the aircraft out to a perpendicular distance of approximately 600 m from the trackline. The aircraft included bubble windows and a belly window, allowing the area underneath the trackline to be observed. Team 1 (forward) consisted of two bubble window observers, each stationed at one side of the aircraft and one data recorder. Team 2 (aft) consisted of one observer on the right bubble and one on the belly window, in addition to a data recorder. The two-team configuration allows for the estimation of detection probability on the trackline^36^. The aircraft location, survey effort status, and viewing conditions (e.g., sea state, glare, visibility) were recorded every 10 s using a data-logging program. Observers updated viewing conditions whenever needed and generally after turns into a new trackline. Upon sighting a marine mammal, sea turtle, or other target of interest (including manta rays), the observer measured the angle from the vertical to the animal (or group) using a digital clinometer. This sighting angle, *θ*, was converted to the perpendicular sighting distance from the trackline (PSD) by PSD = tan(*θ*) × Altitude. While manta rays were not the primary focus of the surveys, observers were instructed to record all large fish sightings (including rays, sharks, tuna, etc.) and collected angles for estimating sighting distances wherever possible.

Sightings and effort data were combined for all SEFSC aerial surveys (Figure S1). An initial investigation of the distribution of PSDs indicated a reduction in the number of sightings very close to the trackline for Team 2, and therefore Team 2 data were truncated at the minimum distance that manta rays were observed and sightings between 0 and 3.2 m were removed from the analysis. Sighting angles were determined for both observer teams based on side (i.e., left, right) and position (e.g., belly, bubble) of each recorded sighting. Because the survey was not specifically designed for manta rays, sighted individuals were not assigned unique identifiers. Using forward team sightings as a reference, aft team sightings were matched to forward team sightings when an equal number of animals were recorded by the aft team within 15 s, on the same side of the aircraft, and with an angle difference of < 15 degrees. Any sightings where the aft team could not have seen the animal due to the sighting angle recorded by the forward team were eliminated from the detection function analysis. Based on histograms and quantiles of sightings distance, the right truncation distance was set at 300 m (Figure S2).

Effective search effort for manta rays was determined in a mark-recapture distance sampling framework using package ‘*mrds’* in R^37^ for “On Effort” sightings by the forward and aft survey teams (Tables S1–S2, Figures S3–S5). The probability of detection and effective area searched were derived using the independent-observer approach assuming point independence^36,37^. A hazard rate MRDS^37^ model was selected by Akaike’s Information Criterion (AIC^38^). Fitting of the detection model considered all possible permutations of covariates that may influence detection probability in the surveyed strip with MCDS and detection probability on the trackline with MRDS, including Beaufort sea state, cloud cover, glare intensity (level of visual obstruction due to sea surface glare), glare coverage (proportion of viewing area obstructed), and turbidity, along with interactions between distance and observer in the MRDS function (Supplemental File 1). All combinations of variables were considered for inclusion, and the best model was selected from the candidate models based on the lowest AIC. For a given trackline segment, search effort was expressed as the multiple of trackline length, estimated detection probability within the strip, and the truncation distance.

#### North Atlantic right whale consortium (NARWC) surveys

The North Atlantic Right Whale Consortium (NARWC)’s sightings database^39^ (Figure S6) serves as a repository for sightings of marine mammals, sea turtles, and large fishes, as well as for corresponding survey effort where available. Survey platforms and protocols for recording manta sightings vary across the many contributors that submit data to the database. Therefore, most contributed datasets were used only for external validation of models, including sightings north of Cape Hatteras and vessel-based surveys. Surveys conducted by the New England Aquarium in a Skymaster airplane during November–March/April in 1989/90, 1990/91, and 1991/92 were the only surveys that reliably recorded sighting distances for manta rays. These surveys were primarily conducted off Florida and Georgia during winter (November–April). Sightings distances for these surveys were reported in intervals, with higher resolution for closer sightings, corresponding to wing-strut markings on the survey platform, and were converted to meters. For distance function fitting, sightings were restricted to on-effort sightings at altitudes of ≤ 366 m with Beaufort sea states of ≤ 4 (Figures S7–S8; Table S3). AIC was used to guide selection of the best-fitting detection function considering possible covariates of sea state, cloud cover, and glare using function ‘*ds*’ in the R ‘*Distance*’ package^40^.

This detection function (Figure S9) was then applied to estimate effective search area for on-effort surveys by the Florida Fish and Wildlife Research Institute (FWRI) from 2002 and 2010–2017, which were also conducted in a Skymaster and consistently recorded manta sightings but did not record detection distance. FWRI surveys were also primarily conducted off Florida and Georgia during winter. Because departures from the trackline for North Atlantic right whale sightings were not explicitly coded as such for the FWRI surveys, and it was unclear whether manta rays would be recorded during this activity, off-track effort was eliminated by dropping waypoints that deviated from the previous heading by > 20 degrees. Visual inspection of tracklines indicated this approach was effective at eliminating loops off the trackline. Sightings from all other NARWC surveys were retained for external validation of model fits, but not included in the distribution modeling input due to lack of clarity regarding whether manta rays would have been explicitly recorded during all on-effort surveys and lack of data suitable for fitting a detection function.

#### New York State energy research and development authority (NYSERDA) surveys

The New York State Energy Research and Development Authority (NYSERDA) contracted with Normandeau Associates Inc. (Normandeau) and teaming partner, APEM Ltd., to use high-resolution, large-format aerial digital imagery to collect data on birds, marine mammals, sea turtles, cartilaginous fish, and other taxa encountered offshore within the New York Offshore Planning Area. Transect surveys of abutting imagery were conducted four times a year between August 2016 and May 2019, with an area of > 3000 km^2^ imaged on each survey—representing > 7% coverage of the > 43,745 km^2^ survey area. Each survey took between six and eight days to complete, depending on weather conditions and flight restrictions. Image resolution was 1.5 cm at the sea surface collected with downward-facing cameras from a flight altitude of 414.5 m and at a flight speed of 220 km/h. Animal targets were extracted from imagery using a combination of detection software and manual review, with detected animals made available to taxonomists for species-level identification through Normandeau’s ReMOTe data portal*.*

Effort was expressed as the swept area within the camera view, and detection probability was assumed to be 100% within the swept area for animals at or near the surface. To estimate size of detected manta rays, we used the Normandeau data portal’s measuring tool to sum across known pixel resolution at the sea surface for manta ray disc width. These measurements were unable to compensate for the unknown depth of the animal in the water column; consequently an element of minor (< 5 cm) error is associated with NYSERDA estimates of manta ray size.

### Distribution modeling

#### Species identification

All animal target identifications by NYSERDA survey detection software underwent a quality-control review of 20% of identifications. Additionally, federally-listed Endangered species went through 100% review of identifications, and 10% of all imagery considered not to contain animal targets also underwent manual review. We re-evaluated taxonomic identifications of all large rays to confirm species identification. Of the 21,539 rays identified in the surveys, 504 were initially identified as manta rays; however, review of digital photo archives by a trained observer determined only 7 were actually manta rays. The majority of misidentified rays were *M. mobular* or *M. tarapacana*. Only those 7 confirmed observations were retained for analysis in the NYSERDA data.

NARWC observers suggested possible misidentification of manta rays with *Mobula tarapacana* and *M. mobular* in aerial surveys north of Cape Hatteras, North Carolina (T. Pusser, pers. comm. to C. Jones). On SEFSC surveys, *M. tarapacana* were identified to species; however, there was a potential for misidentification of *M. mobular*. SEFSC and NARWC observers did not note similar concerns south of Cape Hatteras; however, a supplemental review of photographic archives collected during Normandeau Associates Bureau of Ocean Energy Management aerial surveys suggested that < 30% of Mobulid sightings from North Carolina and South Carolina might be *M. mobular* (J. Robinson Willmott and C. Horn, unpublished data). Photo archives and discussions with Georgia Aquarium observers verified > 1500 manta ray sightings off northeastern Florida but noted that other Mobulid rays were sometimes sighted. Similarly, a supplemental review of photographic archives from FWRI photographic archives from North Atlantic right whale aerial surveys flown in winter (December–March) from approximately Savannah, Georgia (31.93° N) to Cape Canaveral, Florida (28.67° N) revealed 3 of 85 (3.5%) sightings identified as “manta rays” were *M. mobular*, with no recorded sightings of *M. tarapacana*, suggesting a very low potential misidentification rate with other large Mobulids for winter surveys conducted in that region (J. Jakush, pers. comm.).

To minimize bias associated with species misidentification, sightings and effort north of Cape Hatteras (35° N) were excluded from the SEFSC and NARWC surveys for species distribution modeling efforts. All purported “manta ray” sightings from both surveys were retained for external validation of the models and for detection function development, given similarities in size between manta rays and other large Mobulid species.

#### Model selection

Depth was assigned to transect segments from the NOAA National Centers for Environmental Information Coastal Relief Model (CRM), which provides 3 arc-second resolution bathymetry for most areas in the study domain. Data gaps were filled with 1 arc-minute resolution bathymetry from the NOAA ETOP01 database using the R ‘*marmap’* package^41^. Slope was derived from bathymetry using Spatial Analyst in ESRI ArcMap 10.7, with higher-resolution CRM-derived bathymetry and slope retained when available. Satellite observations of daily SST and 8-day averaged chlorophyll-a (Chl-a; Figure S11), along with model-generated estimates of primary productivity (Aqua MODIS, NPP, Global, 2003–present); north-bound water velocity from HYCOM models; and predicted wave height from the Global Wave Model were assigned to daily transect segments from the ERDDAP server using the R ‘*rerddap’* package^42^. Frontal gradients of SST were computed using the R ‘*grec’* package^43,44^.

Data varied with regards to their availability. SST was available for 100% of observations at 1-km resolution and was evaluated because it was hypothesized that manta distributions would be limited by thermal tolerance. Frontal gradients, Chl-a, and primary productivity are all proxies for areas of high productivity, with differing levels of availability and model resolution in the time series. Daily standardized frontal gradient (‘Front-Z’) was available for 100% of observations at 1-km resolution and was computed by dividing SST-derived frontal gradient raster values by the daily maximum within the raster domain. This provided a high-resolution daily image of the position of substantial thermal gradients. Because thermal gradients were standardized to the maximum within the daily image, well-defined fronts were identifiable even during summer warming of surface layers. Preliminary summertime satellite tagging of manta rays off southeast Florida suggested these normalized frontal gradients were useful predictors of relocations in nearshore waters (N. Farmer, unpublished data), potentially because coastal productivity can be tidally-driven or associated with upwelling driven by the intrusion of warm Gulf Stream waters into the nearshore environment. Due to substantial gaps owing to regional cloud cover, Chl-a was averaged across 8 days and was available for 92% of observations at 4-km resolution. Although Chl-a was the most important predictor for manta occurrence in a recent study^30^, here it was limited by spatial (4-km for Chl-a vs. 1-km for SST/Front-Z) and temporal (8-d for Chl-a vs. 1-d for SST/Front-Z) resolution and by the substantial concentration of manta ray observations in tidal coastal waters where remote sensing of Chl-a can become problematic^45^. Primary productivity was available for 90% of observations at 4-km resolution, and was computed by NOAA Fisheries SWFSC as the composite of vertically integrated primary productivity one-day files using the Behrenfield—Falkowski method and satellite-based measurements of Chl-a, incident visible surface irradiance, and SST^46^. Although primary productivity was the most direct measure of the biological proxy we hypothesized might be driving manta ray occurrence, it suffered from the same limitations as Chl-a. Because the east coast of Florida contained the highest concentration of manta ray sightings, we hypothesized that daily north-bound water velocity (1/12° resolution) would be a useful covariate to explore potential bioenergetically-efficient use of the north-bound Gulf Stream and nearshore south-bound counter-current flow for manta rays to remain within a preferred temperature range or areas of concentrated prey with minimal swimming effort. This type of conveyor belt movement has been observed in some satellite-tagged manta rays within the area (N. Farmer, unpublished data). Predicted daily maximal wave height (50-km resolution) was hypothesized to capture manta avoidance of rough water where surface-feeding on zooplankton concentrations would be unlikely due to physical mixing, and surface basking would be bioenergetically costly.

Sightings, effort, and environmental parameters for daily tracklines were summarized to a 10 × 10-km grid for all surveys. The 10 × 10-km model domain encompassed all surveys from all sources. Trackline segments were assigned to grid cells, sightings and effort were summed within each grid cell, depth and environmental characteristics were averaged within each cell, and the maximum observed value for bathymetric slope on the trackline segment was retained. Generalized additive models (GAMs) were fit to all possible permutations of bathymetry and environmental parameters using the ‘*mgcv’* package in R^47^, with log-transformed effort derived from the survey-specific detection functions as an offset. GAMs were fit with a binomial distribution using a logit link function, such that the resultant models describe the probability of species presence, also termed “habitat suitability”^48^ or “habitat preference”^49^. To minimize effects of collinearity, correlated predictor variables (i.e., ρ > 0.7) were not included in the same model (Figure S16). For example, Chl-a and primary productivity were highly correlated and were tested separately but never included in the same model. Because Front-Z was derived from SST rasters, an interaction term for SST and Front-Z was also tested. Models were fit using tensor spline functions of predictor variables limited to 3 knots^47^. Preliminary model-fitting showed a tendency for overprediction at the extremes (especially peak positive values) when extrapolating predictions to other months/years. Constraining to 3 knots eliminated this issue while preserving the functional relationships with environmental covariates.

The best-fitting model was selected by lowest AIC and compared to three competing GAM configurations tiered off the best-fitting GAM by excluding non-significant terms in the model summary. Daily models were generated from the shoreline to the maximum depth surveyed; 1835 m for the SEFSC and 2200 m for the combined surveys, respectively. For each survey, the final model was selected by comparing residual deviance explained and predictive power as evaluated through tenfold internal cross-validation using within-survey sightings and external validation using independent sources. Cross-validation was conducted using the ‘*pROC’* package in R^50^, generating estimates for area under the curve (AUC) and associated false positive and false negative rates for a model fit to 90% of the available data compared to sighting locations from the remaining 10% of the data. External validation was similarly accomplished using ‘*pROC’* to evaluate AUC for the full GAM model compared to sighting records from independent sources, including off-effort sightings from the survey under evaluation. For external validation through AUC, sighting effort was unknown, and assumed equal to mean sighting effort from distance-sampling surveys. As such, external validation outcomes were less reliable for evaluating quality of model fit but useful for relative comparison between models. An additional external validation metric was developed to gauge consistency between SDM predictions and the locations of independent sightings of manta rays*.* We divided SDM predictions for point-specific independent sightings by domain-wide daily median Z-score transformed SDM predictions across valid depths, then centered these transformed scores to zero by subtracting one. The greater the proportion of retained Z-scores above zero, the higher the consistency between SDM predictions and independent observations^51,52^.

We fit models to each survey independently following the approaches above. Due to the limited spatiotemporal coverage of distance-sampling data for the NARWC and NYSERDA data, those SDM results were not reported independently, but were used to generate ensemble models following two approaches. The first (‘weighted ensemble’) was a weighted mean prediction across the best models fit to the three independent surveys (i.e., SEFSC, NARWC, and NYSERDA). Model predictions for each 10-km cell were expressed as a weighted mean across the three surveys, with the complement of the standard error (i.e., 1-SE) of the model prediction from each survey used as the weighting term, to place more emphasis on the model prediction with the least uncertainty at that given location. The second (‘combined surveys’) was developed by fitting a GAM using the iterative fitting process described above to an appended data series of all three surveys combined, with survey-specific sighting effort expressed in the same units of swept area (m^2^).

Annual trends in predicted manta distributions were evaluated by fitting previously described distribution models for SEFSC, NARWC, and combined data to monthly average environmental conditions from January 2003 to December 2019. The monthly weighted mean latitudinal centroid of predicted manta distribution was computed for each model. Seasonal auto-regressive integrated moving average (SARIMA) time-series models with annual and monthly differencing terms were fit using R package “*astsa*”^53,54^. As no annual terms were included in the model, our SDM assumed stationary overall probability of occurrence across years; thus, any interannual differences in mean probability of occurrence would be driven only by differences in the dynamic terms in the model (e.g., SST, frontal gradients, Chl-a) rather than changes in population abundance.

## Results

### Sightings

**Figure 2:**
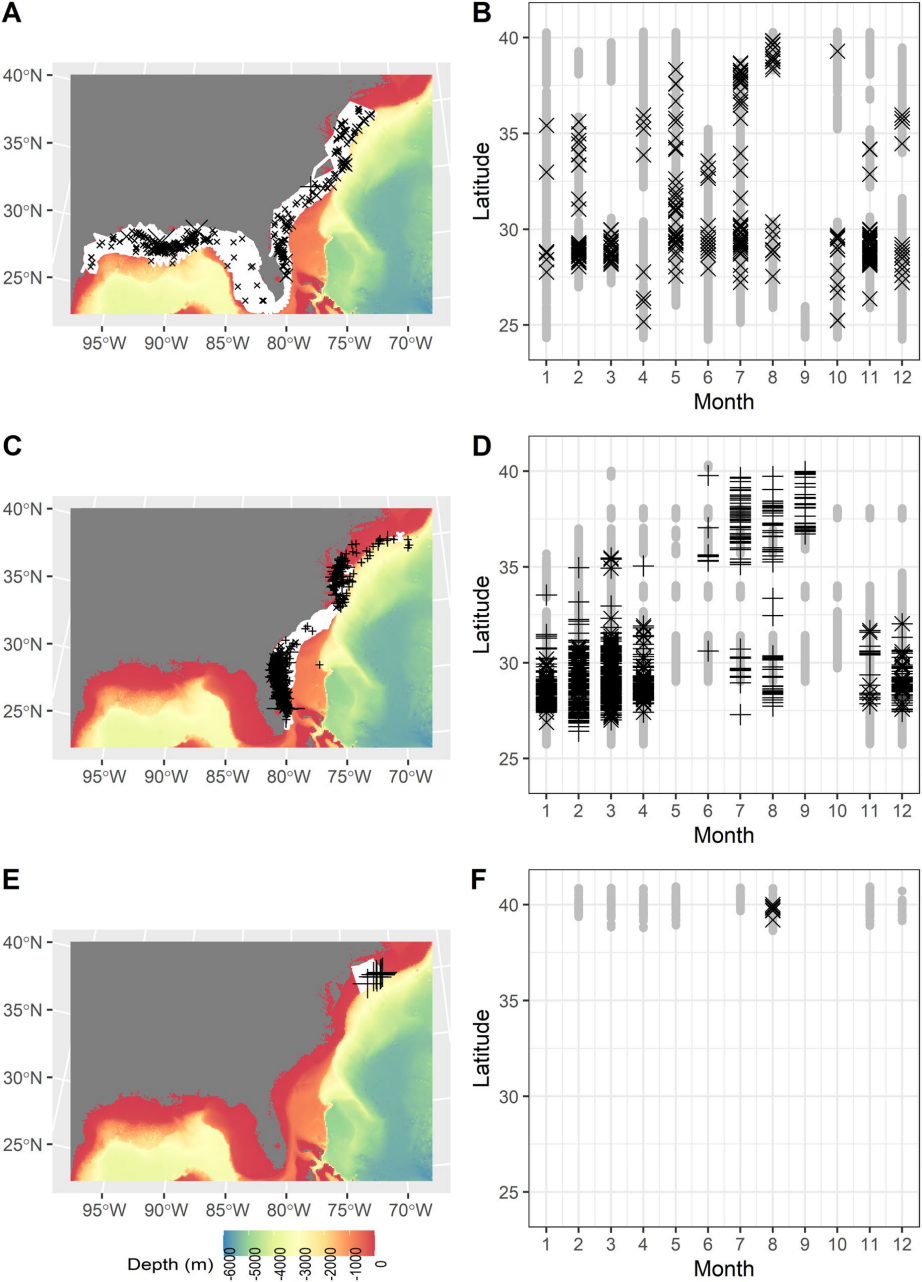
(**A**) Spatial distribution relative to coarse-scale bathymetry (red = shallow; blue = deep) and survey effort (white lines) and (**B**) spatio-temporal distribution of survey effort (gray circles) and manta ray sightings (X: on effort, + : off effort; scaled to number reported within survey) by Southeast Fisheries Science Center (SEFSC), (**C**,**D** ) North Atlantic Right Whale Consortium (NARWC), and (**E**,**F**) Normandeau Associates aerial surveys for New York State Energy Research and Development Authority (NYSERDA). Map generated in R v4.1.2 (https://cran.r-project.org/bin/windows/base/).

Over 5000 manta sightings were identified in the EUS from 1925 to 2020 (Table 1, Fig. 1). Dedicated aerial surveys for manta rays off northeastern Florida funded by the Georgia Aquarium had the most sightings, followed by the multi-contributor NARWC data, which covered much of the U.S. east coast. In the Gulf of Mexico, the highest concentration of sightings was in nearshore waters off Louisiana. On the U.S. east coast, the highest concentration of sightings was in nearshore to shelf-edge waters off Florida and Georgia and nearshore and shelf-edge waters from Cape Hatteras north to New York. Opportunistic manta sightings were also recorded in waters around Puerto Rico and the U.S. Virgin Islands. In the U.S. Virgin Islands, small manta rays were sighted in shallow coastal bays such as Cane’s Bay, Maho Bay, and Francis Bay. In Puerto Rico, the majority of manta sightings were reported from the area surrounding Culebra, Vieques, and Mona Islands. The bulk of manta sightings were recorded between 26° and 30° N, with the highest number of sightings from March through May. The vast majority (82%) of sightings north of 35° N were recorded from June to September (Fig. 2).

**Table 1:** Geographic and temporal range for data sources for manta ray sightings, with number (N) sighted.

Source	Years	N	Latitude (°N)	Area	Description
FAU	2014–2019	99	25–27	Miami Beach to Jupiter Inlet, Florida, USA	Florida Atlantic University Kajiura Lab aerial elasmobranch surveys
FGBNMS	1990–2017	144	27–29	Offshore Texas, USA	Sightings recorded in dive logs by Flower Garden Banks National Marine Sanctuary staff
FMP	2016–2020	142	26–28	Hollywood to Port Salerno, Florida, USA	Boat-based and aerial surveys by trained Florida Manta Project staff, with in-water estimates of size
FSU	2019	1	29–30	Florida Panhandle, USA	Florida State University Grubbs Lab elasmobranch gillnet survey
FWRI	2018–2020	122	27–30	Florida, USA	Florida Fish and Wildlife Research Institute aerial surveys from OBIS (2018–2019) and J. Jakush (pers. comm. to C. Horn, 2020)
GAI	2010–2017	1536	28–30	St. Augustine Inlet to Cape Canaveral, Florida, USA	Georgia Aquarium aerial surveys
IOBIS	1925–2016	1361	36°S–44°N	Global	Ocean Biodiversity Information System open-access data
NARWC	1979–2017	1240	26–40	Florida to Maine, USA	Ship-based and aerial surveys by North Atlantic Right Whale Consortium observers
NEFSC-NEOP	1993–2014	8	35–40	North Carolina to Maine, USA	Northeast Fisheries Science Center Northeast Observer Program observer records in trawl gear
NYSERDA	2016–2017	6	39–41	New York: Long Island to the lower slope roughly between South Wilmington Canyon and Block Canyon	APEM and Normandeau Associates Aerial Digital Baseline Survey of Marine Wildlife in Support of Offshore Wind Energy for New York State Energy Research and Development Authority (NYSERDA) and U.S. Bureau of Ocean Energy Management
Opportunistic	1999–2020	239	17–34	Global	Verified reports to the authors and manta.ray@noaa.gov, social media, press reports
Publication	1993–1994	3	27–29	Indian River Lagoon, Florida, USA	Adams and Amesbury (1998)
SEFSC-AMAPPS	2010–2019	367	27–40	Florida to New Jersey, USA	Southeast Fisheries Science Center Atlantic Marine Assessment Program for Protected Species aerial surveys
SEFSC-GOMMAPPS	2017–2018	109	25–31	Gulf of Mexico (U.S. waters)	Southeast Fisheries Science Center Gulf of Mexico Marine Assessment Program for Protected Species aerial surveys
SEFSC-GOMNRDA	2011–2012	119	25–31	Gulf of Mexico (U.S. waters)	Southeast Fisheries Science Center Gulf of Mexico Natural Resource Damage Assessment program aerial surveys
SEFSC-MS Lab Survey	1982–2015	5	28–30	Gulf of Mexico (U.S. waters)	Southeast Fisheries Science Center Mississippi Lab pelagic longline survey
SEFSC-RVC	2000–2013	6	24–27	Dry Tortugas and Florida Keys, USA	Southeast Fisheries Science Center Reef Visual Census SCUBA-based survey

#### SEFSC surveys

The combined SEFSC surveys covered U.S. waters in the Gulf of Mexico and the U.S. east coast, with comprehensive spatiotemporal coverage of most months other than March and September (Fig. 2A,B). The most sightings and largest groups were reported in the Gulf of Mexico near the mouth of the Mississippi River, the east coast of Florida, and off Cape Hatteras, North Carolina (Fig. 2A). Sightings north of 35° N were reported throughout the year, with the majority in July–August (Fig. 2B; Figure S12). In the mark-recapture distance-sampling framework, the selected MCDS model included cloud cover, and the selected MRDS function included glare and an interaction between observer and distance (Supplemental File: SEFSC Surveys). Average detection probability within the 300-m swept area was 39.4% (CV = 11.9%).

#### NARWC surveys

The NARWC surveys covered the U.S. east coast, with temporal coverage of all months over the span of the survey; however, only a limited subset, primarily off Florida and Georgia in the winter, contained sufficient distance-sampling information to be used in the SDM (Fig. 2C,D). The most sightings and the largest groups were sighted off Florida and Georgia (Fig. 2C). Sightings were reported in all months except May and October (Fig. 2D). The vast majority of sightings north of 35° N were reported during June–September (Fig. 2D). The selected detection function was a half-normal key function of sea state (Supplemental File: NARWC Surveys). Average detection probability within the 348-m swept area was 55.5% (CV = 48.6%).

#### NYSERDA surveys

The NYSERDA surveys covered the nearshore to offshore marine environments of New York, with temporal coverage during the spring/summer of 2016–2019 and fall/winter of 2016–2018 (Fig. 2E,F). All manta ray sightings and > 99% of Mobulid ray sightings were in summer. Despite comprehensive coast to shelf survey coverage, manta sightings were exclusively in August on the continental shelf edge.

### Distribution modeling

**Figure 3:**
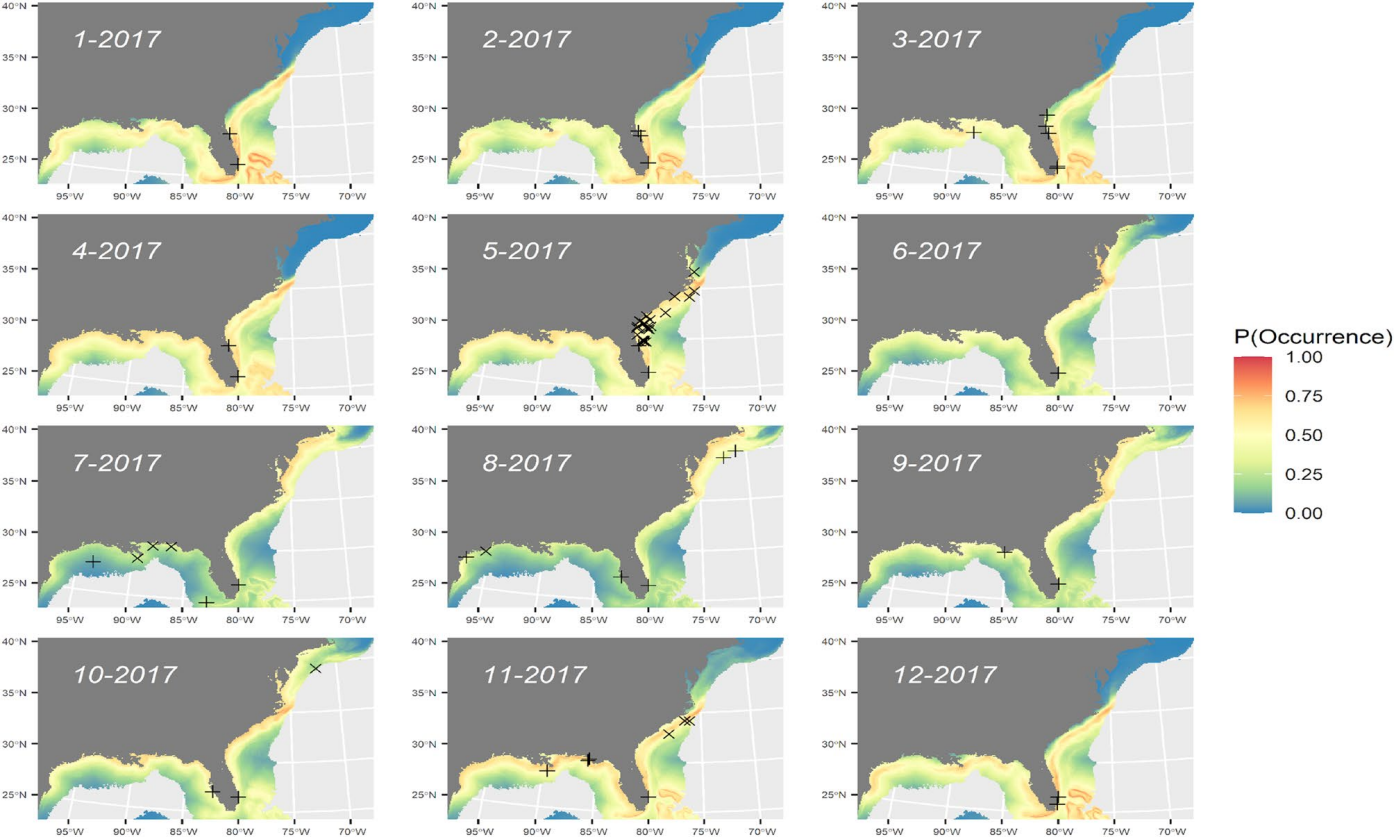
Predicted probability of occurrence for manta rays by SEFSC surveys for monthly average environmental conditions in 2017 with overlay of internal (X) and external (+) validation points. Map generated in R v. 4.1.2 (https://cran.r-project.org/bin/windows/base/).

SDMs generated from combined SEFSC surveys explained 4–5% of residual deviance (Table 2). AUC for internal and external validation were comparable and indicated “acceptable” model fits^55^ (Table 2). The deviance explained for single-covariate models is given in parentheses in the following summary. Sighting probability was highest at SSTs from 17 to 32 °C (3.8%), with peak probability around 23 °C. Sighting probability was highest close to shore (0.8%) at strong thermal fronts (0.5%). The final SEFSC survey SDM predicted fairly high probability of occurrence (> 25%) during most of the year south of Cape Hatteras, North Carolina (Fig. 3). Peak probability of occurrence in the Gulf of Mexico and south of Cape Hatteras was predicted during cooler months (November–April), with peak probability of occurrence north of Cape Hatteras during warmer months (May–October).

**Table 2:** Model fit summaries for different survey datasets with akaike information criterion (AIC), residual deviance explained (DevExpl), and area under the curve (AUC) from ten-fold internal (int) and external (ext) cross-validation from independent samples.

Survey	Model	AIC	Dev expl (%)	AUC	AUC
				(int) (%)	(ext) (%)
SEFSC	offset(log(striparea)) + Front_Z × SST + Front_Z + SST + pp + Depth_m + Slope_deg + DfromShore	1808	5.4	74.9	74.8
SEFSC	offset(log(striparea)) + Front_Z + SST + DfromShore + pp	1809	5.2	74.6	74.6
SEFSC	offset(log(striparea)) + Front_Z + SST + Depth_m + ChlA	1890	4.4	73.3	73.3
SEFSC	offset(log(striparea)) + Front_Z + SST + ChlA	1895	4.1	72.6	72.6
**SEFSC**	**offset(log(striparea)) + Front_Z + SST + DfromShore**	**1997**	**5.3**	**75.0**	**75.0**
Combined surveys	offset(log(striparea)) + Front_Z × SST + SST + pp + Depth_m + Slope_deg	3328	18.5	84.8	84.8
Combined surveys	offset(log(striparea)) + Front_Z × SST + SST + Depth_m + Slope_deg	3563	19.0	84.8	84.8
**Combined surveys**	**offset(log(striparea)) + Front_Z** × **SST + SST + Depth_m + Slope_deg + ChlA**	**3368**	**19.3**	**85.2**	**85.2**
Combined surveys	offset(log(striparea)) + SST + Depth_m + Slope_deg + ChlA	3395	18.5	85.0	85.0
**SEFSC, NARWC, and NYSERDA**	**Weighted ensemble SDM**	**1163**	**18.7**	**84.7**	**84.7**

Selected model in bold for each survey. ‘Combined Surveys’ denotes models fit to data from the SEFSC, NARWC, and NYSERDA surveys.

**Figure 4:**
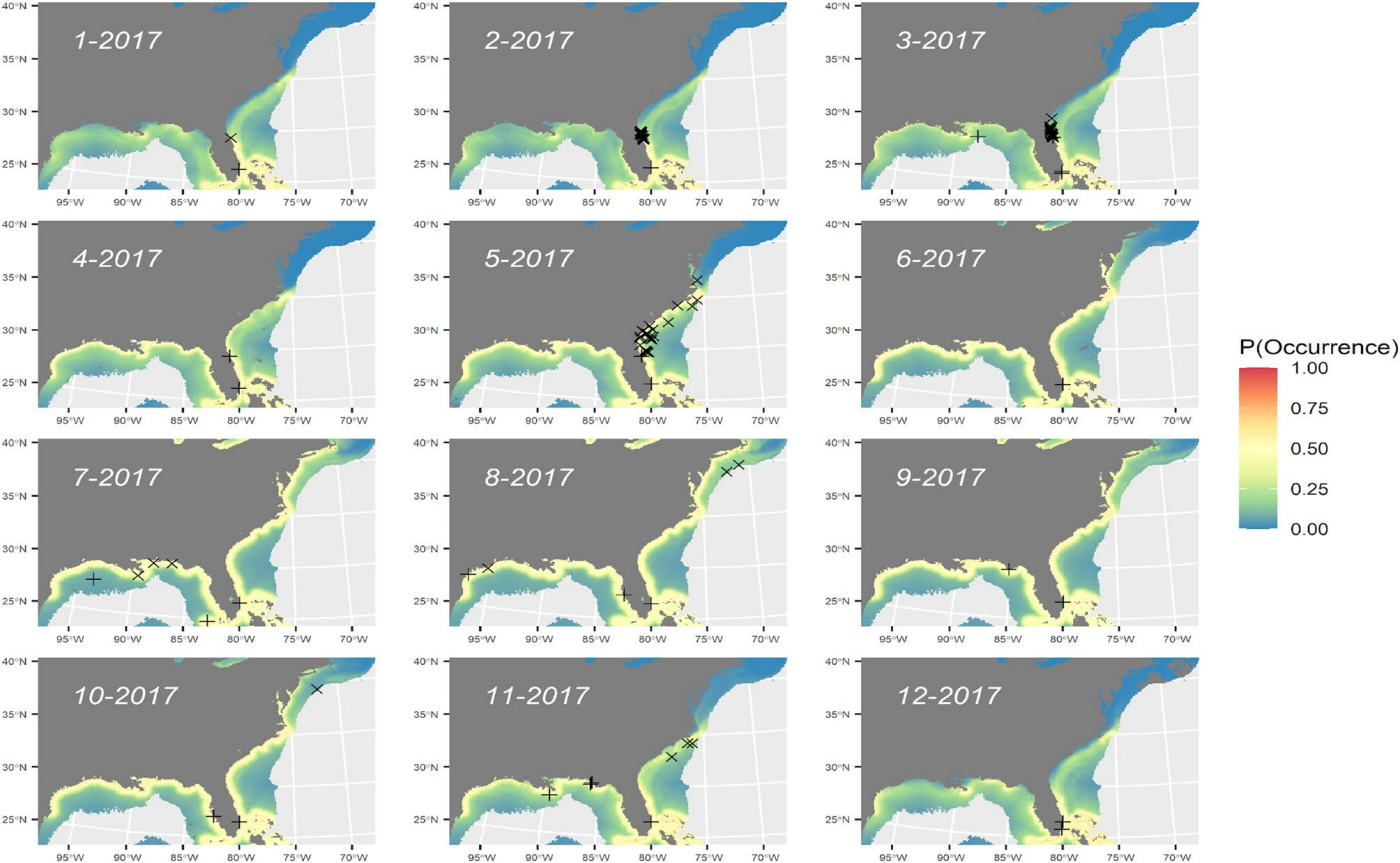
Predicted probability of occurrence for manta rays by a weighted ensemble of model predictions from SEFSC, NARWC, and NYSERDA surveys for monthly average environmental conditions in 2017 with overlay of internal (X) and external (+) validation points. Map generated in R v. 4.1.2 (https://cran.r-project.org/bin/windows/base/).

The ‘weighted ensemble’ of SDMs generated from the SEFSC, NARWC, and NYSERDA surveys had an “acceptable” predictive fit^55^ (Table 2). The weighted ensemble SDM explained 18.7% of residual deviance and predicted a relatively uniform probability of occurrence in nearshore environments (Fig. 4; Figure S13).

**Figure 5:**
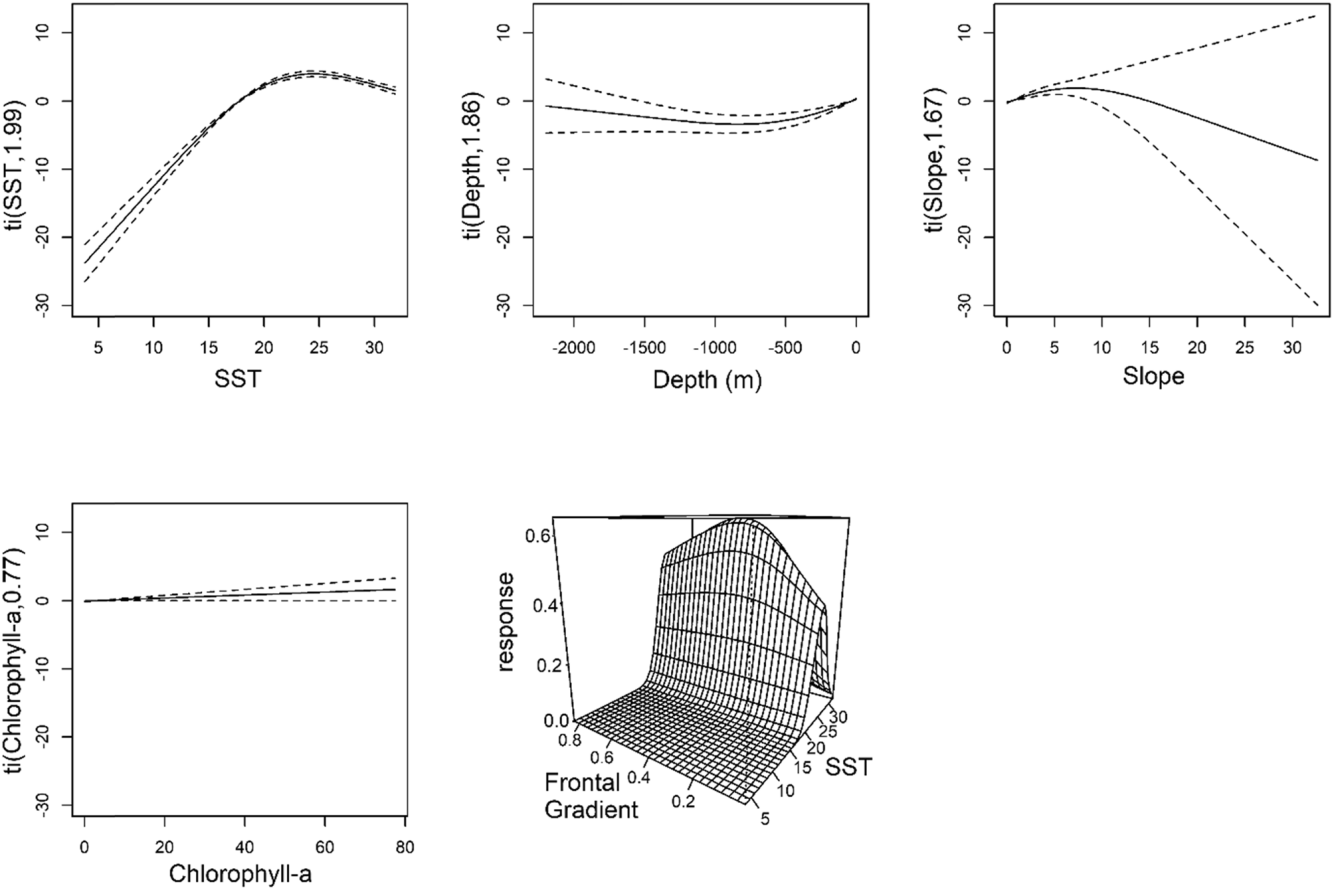
Significant species distribution model GAM predictor terms for combined surveys (SEFSC, NARWC, and NYSERDA) model, including sea surface temperature (SST; °C), Z-transformed SST frontal gradients, depth (m), Chlorophyll-a concentrations (mg/m^3^), and bathymetric slope (degrees).

The ‘combined surveys’ SDM that integrated sightings and effort from SEFSC, NARWC, and NYSERDA surveys explained 19.3% of residual deviance. Deviance explained for single-covariate models is listed in parentheses in the following summary. The combined surveys SDM predicted higher probabilities of observation with SST between 20 and 30 °C (17.5%), increasing Chl-a (8.5%) concentrations, moderate Front-Z (< 1%), nearshore and shelf-edge depths (1.8%), and moderate bathymetric slopes (< 1%) (Fig. 5; Figure S14). AUC for internal and external validation were comparable and indicated “excellent” model fits^55^ (Table 2).

**Figure 6:**
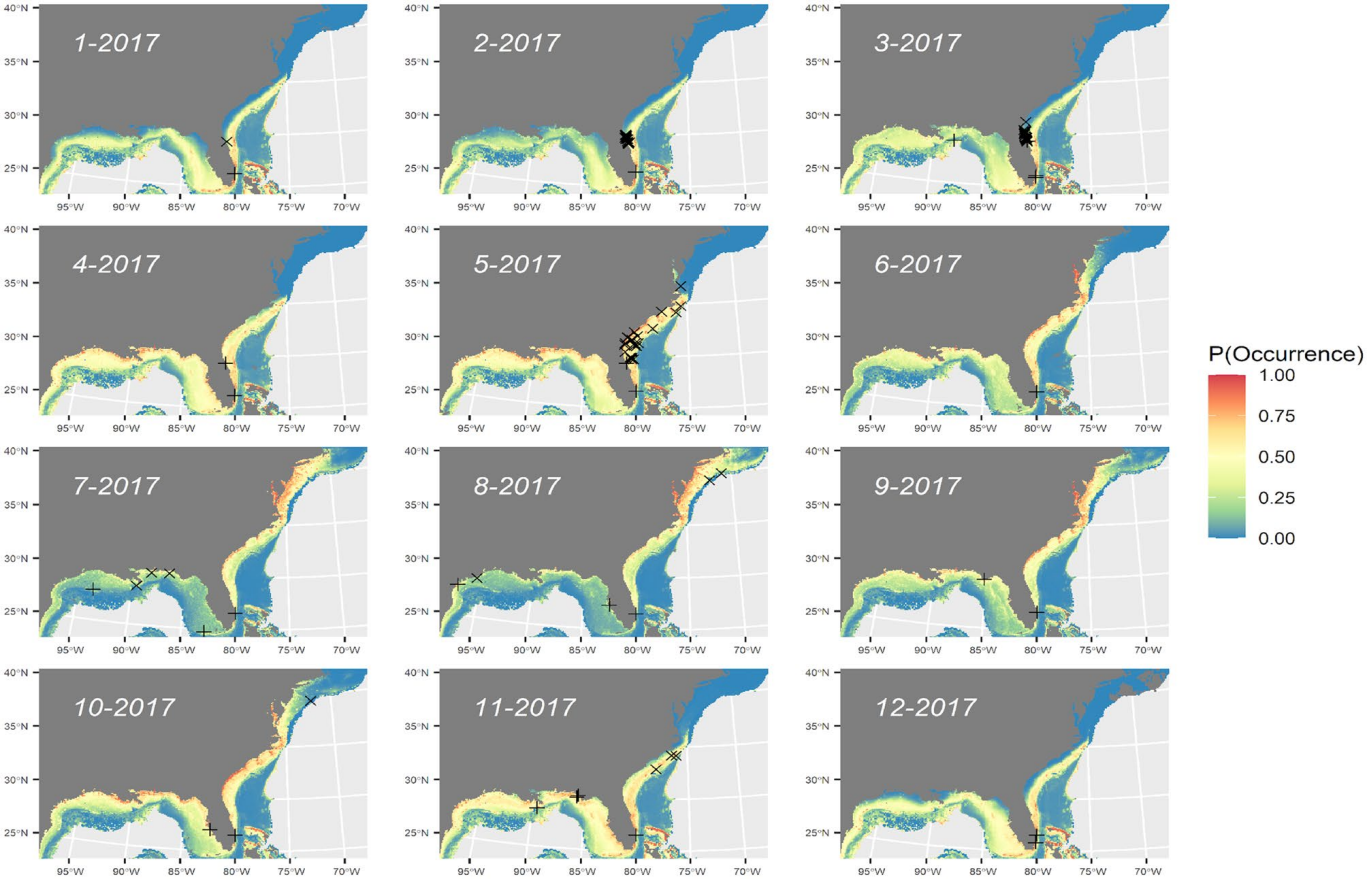
Predicted probability of occurrence for manta rays for combined surveys (SEFSC, NARWC, and NYSERDA) model for monthly average environmental conditions in 2017 with overlay of internal (X) and external (+) validation points. Map generated in R v. 4.1.2 (https://cran.r-project.org/bin/windows/base/).

The combined surveys SDM predicted highest probabilities of detection at offshore sloped habitats (e.g., seamounts) and in the nearshore environments of the Mississippi River delta from April to June and again in October (Fig. 6).

**Figure 7:**
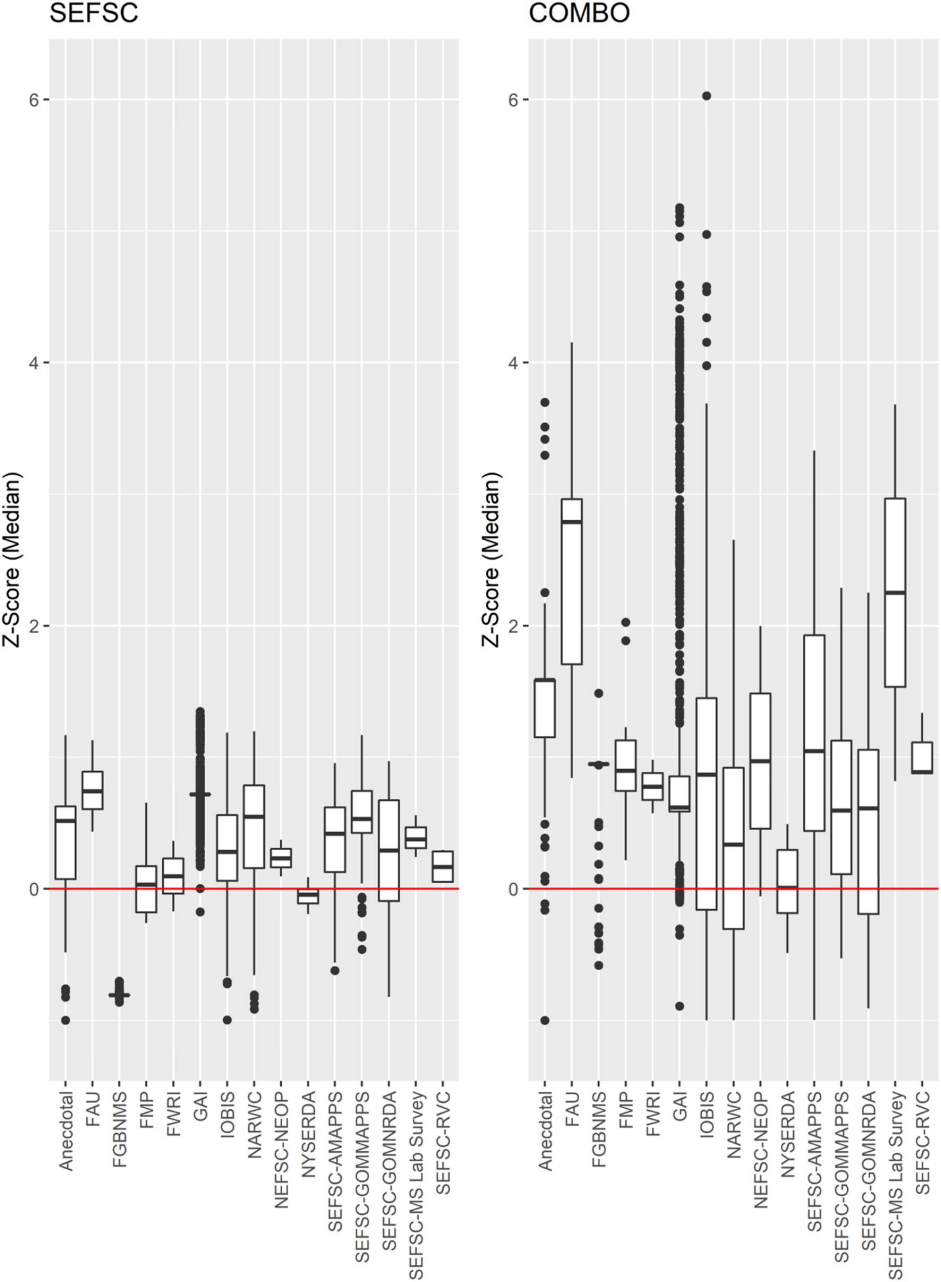
External validation of predictive utility of SEFSC and combined (“COMBO”: SEFSC, NARWC, and NYSERDA) surveys species distribution models, showing predicted median Z-score standardized probabilities for independent observations for manta rays (see Table 1). Positive Z-scores (above red line) indicate consistency between independent observations and model predictions, with higher Z-scores indicative of greater predictive utility.

External validation of the SEFSC and combined survey models was challenged by a lack of necessary environmental data for many independent manta ray observations. Some observations were from periods before satellite data were collected; others were too close to shore to generate frontal gradients. However, where models could be successfully fit, external validation suggested high predictive utility to independent observations, especially for the combined survey model (Fig. 7). The median Z-score standardized probabilities of observation were significantly greater than 0 for both surveys [SEFSC: *t*(14,657) = 241.39, *p* < 0.0001, $\overline{\mbox{x}}$ (95% CI) = 0.592 (0.588–0.597), Combined: *t*(4036) = 59.86, *p* < 0.0001, $\overline{\mbox{x}}$ (95% CI) = 0.873 (0.844–0.902)], confirming that SDM predictions were highly consistent with independent observations of manta rays (Fig. 7). The SEFSC surveys SDM provided the highest predictive utility when compared to NARWC sightings data, but relatively poor predictive utility for manta ray sightings by the Florida Manta Project (FMP) in the juvenile habitats of southeast Florida^26^ and NYSERDA sightings. The combined surveys SDM improved predictive utility across all surveys.

All SDMs predicted similar spatio-temporal distribution trends (Figs. 3, 4, 6), with higher probabilities of detection on the inside edge of the Gulf Stream from January to April, and peak nearshore occurrence off northeastern Florida and Georgia during April. The predicted distribution extends northward during May and then the peak relocates north of Cape Hatteras from June to October. The peak then collapses back to nearshore Georgia and Florida’s east coast, and the Gulf of Mexico from November to December. In the Gulf of Mexico, the peak was concentrated near the Mississippi River delta during the spring and fall, but more dispersed and farther from shore during the remainder of the year. This pattern corresponds to seasonal fluctuations in productivity, as inferred from Chl-a, and SST (Figure S11). All SDMs predicted high concentrations of manta rays in Chesapeake and Delaware Bays; however, there are no reported sightings in these areas and this model prediction is likely confounded by high Chl-a concentrations associated with terrestrial outflow.

**Figure 8:**
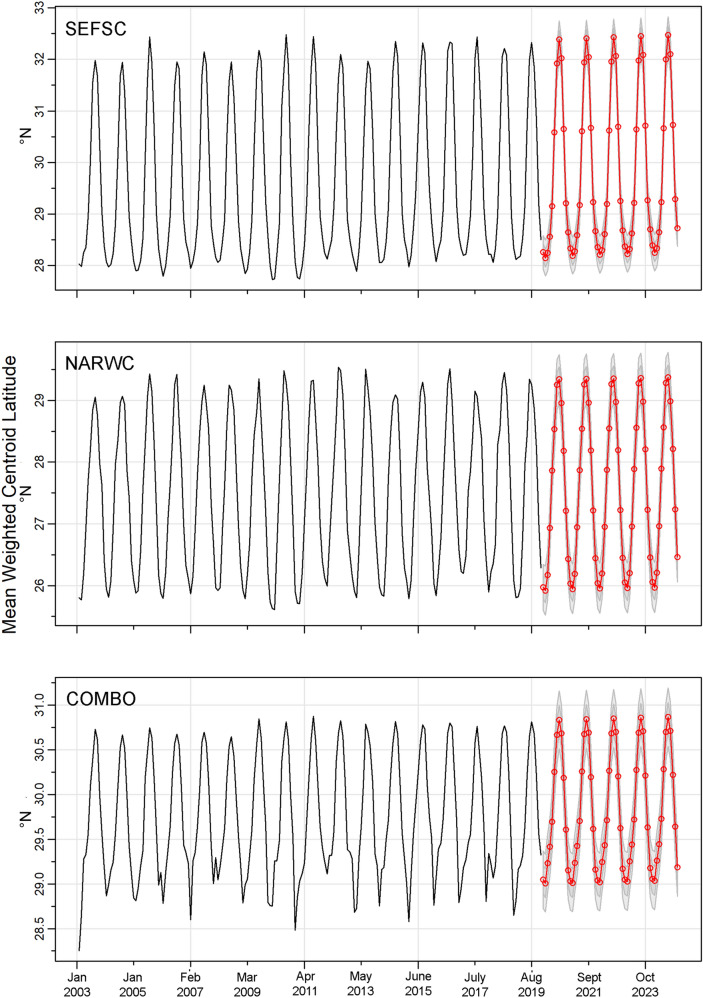
Time-series of predicted mean weighted latitudinal centroid for best-fitting manta ray species distribution models generated from SEFSC, NARWC, and combined surveys (“COMBO”) from January 2003 to December 2019 with seasonal auto-regressive integrated moving average (SARIMA) mean (red) and ± 1 and ± 2 standard error (gray) forecasts for January 2020 to December 2024.

Plots were generated from January 2003 to December 2019 for the SEFSC SDM (Figure S12) and the combined surveys SDMs (Figure S13). SARIMA models for SDMs fit to SEFSC, NARWC, and combined surveys all indicated a significant monthly trend in weighted mean central latitudinal distribution (SEFSC: seasonal model average *sma1* = –0.98, *t*(189) =  − 2.63, *p* = 0.0093; NARWC: *sma1* =  − 0.96, *t*(189) =  − 4.94, *p* < 0.0001; Combined Surveys: *sma1* =  − 1.00, *t*(189) =  − 10.3241, *p* < 0.0001). For both the SEFSC and NARWC models, SARIMA models indicated a significant northerly trend in weighted mean central latitudinal distribution over the 2003–2019 time-series (SEFSC: auto-regressive *ar1* = 0.42, *t*(189) = 6.51, *p* < 0.0001; NARWC: *ar1* = 0.39, *t*(189) = 5.89, *p* < 0.0001). Forward-projection of the SARIMA model predicted a continued northern shift in the overall distribution of manta rays from 2020 through 2024 (Fig. 8).

Size estimates for manta rays were limited, but suggest that smaller animals, likely juveniles, were more common in the warmer waters of the southeast U.S. (e.g., Gulf of Mexico, Florida, and Georgia; Figure S15), whereas larger individuals were observed farther north in the EUS. Anecdotal observations, supported by photo and/or video, of small (1.7 m) individuals from shallow bays in the U.S. Caribbean were also reported.

## Discussion

This study provides a comprehensive evaluation of decades of aerial survey and other sightings for manta rays off the eastern United States (EUS). Combining surveys allowed us to expand our spatio-temporal sampling domain. Innovative approaches to SDM validation using independent sightings data allowed us to demonstrate the substantial predictive utility of our model outputs. Using a distance-weighted sampling framework, we determined that manta ray occurrence in the EUS is influenced primarily by temperature, with a clear expansion to the north during warmer months. Within this thermal range, manta rays occur most frequently either nearshore or along the continental shelf-edge, at locations best predicted by proxies for productivity such as thermal fronts, bathymetric slope, and high chlorophyll-a concentration. Nearshore occurrence, where manta rays would be most consistently impacted by anthropogenic stressors, was most common near the Mississippi River delta in the Gulf of Mexico and along the beaches and inlets of southeastern Florida.

A major conservation priority for manta ray recovery is to improve our understanding of movement and seasonal distribution patterns to inform future management measures for minimizing impacts to the species during key life history functions^56^. Globally, manta rays inhabit tropical, subtropical, and temperate bodies of water and are commonly found offshore, in oceanic waters, and near productive coastlines^9,26,57^ including in estuarine waters near oceanic inlets^58–60^ (J. Pate, unpublished data). Our analysis of decades of manta ray sightings across several different aerial survey platforms in the EUS indicated manta rays were most commonly detected at thermal fronts in productive, warm (20–30 °C) nearshore and shelf-edge waters (Supplemental Video). For the Gulf of Mexico, peak occurrence and observations were clustered off the Mississippi River delta, an area of known high concentrations of large zooplankton (see Figs. 3 and 7 in^61^). Nearshore habitats in the Gulf of Mexico may contain important food sources for manta rays^61^. Similarly, zooplankton biomass estimates^62^ suggest high concentrations of potential manta ray prey in the Mississippi River plume, Florida coastal waters, the upwelling zone near Cape Hatteras, North Carolina, and the northeastern United States shelf-edge areas covered by the NYSERDA surveys. Upwelling caused by shearing and frontal eddies associated with Gulf Stream movement across the continental shelf provides a consistent source of phytoplankton that in turn may create useful prey fields for manta rays at shelf-edge locations^63,64^. Similarly, manta rays appear to utilize nearshore, tidally-driven zooplankton patches^65,66^, especially along Florida’s east coast.

It is noteworthy that > 99% of all rays (all species) observed by the NYSERDA surveys were observed in the spring/summer, despite nearly equal levels of survey effort in the fall/winter. Environmental temperature directly dictates body temperature for most elasmobranchs^67–69^. Many physiological rates scale with temperature according to a thermal performance curve, with performance gradually increasing up to an organism’s optimum temperature, and then quickly declining as temperatures approach lethal levels^70–73^. Manta and other Mobulid rays routinely exhibit basking behaviors presumably to elevate body temperatures after making excursions into deeper, colder habitats^74^. Cold winter air and sea surface temperatures in the western North Atlantic Ocean likely create a physiological barrier to manta (and other) rays that restricts the northern boundary of their distribution.

In this study, SST was the strongest single predictor of manta distribution, with most occurrences between 20 and 30 °C, with a peak around 23 °C. These temperatures are consistent with those reported by other studies for manta rays off the U.S. east coast^9,75^, the Yucatan peninsula^21,76^, New Zealand^77^, and Indonesia^78^. Off the northern Yucatán peninsula, manta ray occurrence was seasonal and associated with high SSTs (> 27 °C), high primary productivity (4500 mg C m^−2^ day^−1^), shallow waters (< 10 m), relatively short distances to shore (< 50 km), and shallow bottom slope (< 0.5°)^21^. In the Western Central Atlantic, Chl-a, bathymetric slope, and SST were important drivers of manta distribution; however, SST was the least important driver, possibly due to the lower variability in SST in the Western Central Atlantic relative to the EUS^30^. By contrast with strong seasonal trends to the north, our SDM predicted year-round occurrences in the southeastern Florida area, which has been identified as a potential nursery habitat^26^. We also identified a possible trend of larger manta rays being sighted farther north (Figure S15). Both southeastern Florida and the other identified potential nursery habitat at FGBNMS^24,25^ maintain mean temperatures in the 20–30 °C range year-round (see Fig. 8.1 in ^79^). Juveniles may grow faster in warmer water^80^ and be more resilient to coastal temperature fluctuations and higher maximum temperatures^73,81,82^, whereas adults are capable of migrating long distances and may be better able to take advantage of seasonal blooms in productivity^73^. The higher thermal inertia of larger manta rays may also allow them to forage in colder waters for longer intervals^83^.

The combined surveys model indicated highest probability of occurrence at moderately-sloped nearshore and shelf-edge habitats with moderate SST fronts and high concentrations of Chl-a; all proxies for high primary production and associated manta prey availability (Fig. 5). Due to the lack of spatially comprehensive zooplankton and micronekton sampling data, we were unable to explicitly test associations between manta rays and prey availability. However, strong associations were observed across data sources between manta ray sightings and proxies for productive upwelling zones. Models predicted higher concentrations of manta rays in warm (20–30 °C) waters from the coast to the shelf south of Cape Hatteras, approximately corresponding with the inside edge of the Gulf Stream current. Offshore, the inside edge of warmer Gulf Stream waters passing the continental shelf provides a consistent source of upwelling and productivity^84^. Similarly, nearshore tidal fronts provide mixing and nutrient concentrations to support high concentrations of potential prey items^85–87^. The offshore, northbound flow of the Gulf Stream is offset by the nearshore, southbound counter-current flow^88^, and may provide a bioenergetically favorable ‘conveyor belt’ for filter-feeding manta rays to efficiently forage while remaining within thermally-optimal conditions. Satellite-tagging studies are needed to evaluate individual movement patterns and quantify population connectivity. Similarly, expanded sampling and associated modeling efforts are needed to better understand the spatio-temporal distribution of manta ray prey resources. We observed a dome-shaped relationship with temperature and significant seasonal and interannual trends in the centroid of manta distributions. Given that the major impact of climate change on Mobulids is likely to be the projected decline in zooplankton in tropical waters^31^, future research should identify manta prey in the EUS, determine the environmental drivers of their prey distribution, and evaluate how those distributions are likely to shift under climate change scenarios.

Retrospective analyses of NYSERDA data and discussions with aerial observers (Table 1) suggested that *M. mobular* and *M. tarapacana* were frequently misidentified as manta rays, especially north of Cape Hatteras, North Carolina. We attempted to minimize this uncertainty by only including photographically-verified sightings north of Cape Hatteras in our modeling efforts. Interviews with observers and reviews of 100s of photos suggested extremely low misidentification rates south of Cape Hatteras; however, photos were not available for all sightings. The Normandeau Associates/APEM digital photo archives for NYSERDA and BOEM data allowed us to compare sightings locations for confirmed species identifications of large Mobulids, including manta rays, and revealed substantial overlap in species habitat utilization along the U.S. East Coast’s continental shelf from South Carolina to New York (Figure S10). Due to a lack of distinguishing dorsal features, we were unable to distinguish the putative third species or subspecies of *M. birostris* (*M. sp. cf. birostris sensu*^9^) resident in the Gulf of Mexico^27,28,89^ and possibly southeastern Florida^26^. Without genetic testing, species identification cannot be completely validated^27,28,90^. The misidentification of *M. birostris* with other Mobulids is likely an issue in other holistic descriptions of manta ray distribution^30^ where observer and OBIS sightings have been used but unverified through interviews and photographs. In 2018, NOAA developed and distributed observer aerial survey guides that should improve the reliability of Mobulid sighting data collected from 2019 on. At present, our SDMs may to some extent represent a shared habitat utilization of Mobulid species, heavily weighted towards manta rays, including *M. sp. cf. birostris sensu*^9^.

Similarly, our model does not account for any differences in depth utilization (e.g., availability bias). Mobulid rays do not have facultative surface breathing requirements but engage in surface feeding^6,91^ and apparent basking behavior^92,93^. Three satellite-tagged manta rays tagged in the shallow (< 15 m) southeast Florida nursery habitat and one in the deeper (> 15 m) FGBNMS nursery habitat (N. Farmer, unpublished data) showed daytime use of the upper two meters of 25% ± 10% and 7%, respectively. Manta rays are frequently observed surface feeding in southeast Florida (J. Pate and N. Farmer, unpublished data), and FGBNMS staff report occasional boat-based sightings of apparently basking manta rays (M. Nuttall, pers. comm.). Satellite tagging has revealed seasonal shifts in primary depth utilization that appeared to track the depth of the thermocline, possibly as a cue to identify regions of high zooplankton density^94^. If manta rays spend proportionally more time in waters below the visual observation depth at particular locations or during particular times of year, a model driven by aerial survey observations within the visible surface layer (approximately 1–2 m depth) would underestimate their utilization of those areas. Preliminary tagging data from the area suggest the model may underestimate utilization of deeper waters, at least during some times of year, given that shallow waters may have a shallower mixed-layer depth and in very nearshore areas there is no refuge from the visible depth range. Similarly, aerial surveys were carried out during daylight hours; thus, if manta rays spend more time at depth during the daytime at certain locations, SDM results would underpredict their utilization of these areas. This seems less likely; consistent with other studies^94^, all four satellite-tagged manta rays spent more time at depth during the night (N. Farmer, unpublished data). Future applications of these data sources will attempt to quantify spatiotemporal misidentification rates following the introduction of observer training guides in 2019 and also attempt to generate estimates of abundance that are adjusted for depth use reported from regional satellite tagging efforts.

In the United States, NOAA is charged with promoting the recovery of giant manta rays. Under Section 7 of the ESA, agencies must consult with NOAA to ensure their proposed actions do not jeopardize the survival of listed species. Understanding the distribution, abundance, migration patterns, and site fidelity of manta rays is essential for accurately estimating the impact of proposed activities. Anthropogenic impacts to individuals can be estimated as the product of: (1) the probability of an activity occurring in an area; (2) the probability of an individual being in an activity area, expressed as a distribution model; (3) the duration of exposure of the individual to the activity; and (4) the probability of the activity impacting the individual, often expressed as a dose–response curve^95^. Managers can work with action agencies to time activities when risk is minimized, and to enact conservation measures to reduce level and duration of exposure. Our SDMs will help managers compute the likelihood of an interaction and recommend environmental windows to minimize risk. Even when duration of exposure and probability of adverse effects are unknown, relative risk assessments can be used to identify preferred alternatives^96^. To more accurately determine anticipated take of giant manta rays from proposed actions, further information is needed on movements, site fidelity, depth utilization, and responses to anthropogenic stressors.

The most significant threats to the recovery of manta rays across their global range are intentional harvest and bycatch in fisheries^97^. Manta rays are targeted or caught as bycatch with virtually every fishing gear type, including small-scale fisheries using driftnets, gillnets, harpoons, gaffs, traps, trawls, and longlines; and large-scale fisheries using driftnets, trawls, and purse seines^98^. Our SDMs will help managers identify areas of spatial and temporal overlap between giant manta rays and commercial fisheries, which could be used to reduce bycatch rates in the EUS. Similarly, a better understanding of the spatiotemporal distribution of the species may help improve precision of bycatch estimates by controlling for relative availability of the species to the gear on any given set and allocating observer coverage to areas of higher bycatch concern. For example, preliminary analysis of 2019–2020 data from the EUS shrimp-trawl fishery estimated mean take of manta rays of nearly 1700 individuals/yr^99^; however, uncertainty was very high given the short time series and limited data. Although observer coverage on shrimp trawls in the EUS is around 1%, the relative observer coverage on shrimp trawls with trawl effort spatially weighted by manta probability of occurrence was less than 0.09% (N. Farmer, unpublished data).

A major NOAA recovery priority for giant manta rays is to investigate the impact of other threats to the species (e.g., foul-hooking, vessel strikes, entanglement, climate change, pollution, tourism) through research, monitoring, modeling, and management^56^. Our SDMs and regional observations suggest that manta rays are frequently associated with nearshore habitats; as such, they are at elevated risk for exposure to a variety of contaminants and pollutants, including brevetoxins, heavy metals, polychlorinated biphenyls, and plastics^26,100,101^. Many of these toxins can bioaccumulate over decades in long-lived filter feeders, leading to a disruption of biological processes (e.g., endocrine disruption), and potentially altering reproductive fitness^102^. Coastal and lagoon habitats are especially sensitive to habitat degradation, pollution, and sedimentation^103^.

There is a strong management interest in understanding the inshore extent of manta movements in bays and tidal inlets. SDM predictions suggest seasonal trends with high probability of occurrence in large bays (e.g., Tampa Bay, Chesapeake Bay); however, reported sightings in bays are extremely limited. It is unclear if this is due to reduced water clarity, rarity of use, or very low levels of survey effort. Manta rays have been reported in bays and inlets in Brazil^29,60^, and we verified several anecdotal reports of use of shallow tropical bays in the U.S. Caribbean. Future efforts will seek to evaluate EUS nearshore sightings relative to currents, tidal phase, and salinity. Manta rays are frequently reported in nearshore environments of southeastern Florida^26^ and somewhat regularly in the U.S. Caribbean. Georgia Aquarium and partners recorded high numbers of manta rays around St. Augustine, Florida during dedicated aerial surveys in 2010–2017 (Table 1); however, the timing and frequency of these observations was variable both seasonally and interannually, with the peak only lasting a few weeks. SDMs capture these trends (Supplemental Video 1), predicting a spring and fall peak in the survey area with the spring peak varying between March and June.

Florida, and southeast Florida specifically, has the highest number of registered recreational vessels and licensed recreational anglers in the U.S., and likely the world^104^. Manta rays are exposed to exceptionally high levels of vessel traffic as well as hook-and-line fishing gear from boats and piers. Manta rays are often observed foraging on tidal outflows at major inlets in southeast Florida, leading to frequent overpasses by vessels moving at high speeds^26^ and vessel strike injuries (J. Pate, unpublished data). Casting in the vicinity of large manta rays is a major component of the recreational cobia fishery along most of the Florida coast^105^ (Pate J, unpublished data, Farmer NA, unpublished data). Fishing line entanglement was documented on 27% of individuals in southeast Florida, along with vessel strike injury and rapid wound healing^26^. Outreach focused on preventing recreational fishery interactions with manta rays, encouraging use of environmentally-friendly tackle, and fostering engagement with anglers as citizen scientists is needed in southeast Florida^106^. Our SDMs will allow managers to more effectively time and coordinate management strategies, including targeted outreach efforts and developing spatiotemporal ‘windows’ for action agencies to reduce the risk of manta interactions.

A major priority for manta ray conservation is to improve understanding of population distribution, abundance, trends, and structure through research, monitoring, and modeling^31,56^. Our preliminary presence-absence modeling approach uses distance-weighted methods to control for perception bias. With further satellite-tagging data on manta ray movements and dive profiles, we may be able to address availability bias for animals that are underwater and apply similar methods to determine population abundance. Further genetic analyses are needed to resolve the taxonomic status and relative abundance of *M. birostris* and *M. sp. cf. birostris sensu*^9,27^. More telemetry studies are also needed to evaluate whether giant manta rays within the Gulf of Mexico and northwestern Atlantic Ocean constitute one large, mixed population, or exist as isolated subpopulations^94^. Observing rare species incurs a heavy cost in time, resources, and boat fuel. Our findings suggest that SDMs combined with real-time satellite data may be used to effectively target manta rays for scientific study, including the attachment of satellite tags and acoustic tags in collaboration with acoustic telemetry networks^107,108^ to inform movements, site fidelity, and dive patterns for these highly mobile animals. Individual impacts can then be summarized across the population to evaluate population consequences of disturbance in the context of population recovery^95^.

## Supplementary Information

Supplementary Information 1.

Supplementary Video 1.

Supplementary Information 2.
