# Supplementary material for: The distribution of manta rays in the western North Atlantic Ocean off the eastern United States
Source: Sci Rep. 2022 Apr 21;12:6544. doi: 10.1038/s41598-022-10482-8 (PMC9023537; doi:10.1038/s41598-022-10482-8)
Supplement: Supplementary file 1 — Supplementary Information 1. [file 41598_2022_10482_MOESM1_ESM.docx]

### Link to Supplemental Video

Supplemental Video 1 (EUS): <https://drive.google.com/file/d/163WevvwTarxoPho3NSXp_Gj1nslZvqZU/view?usp=sharing>
