# Supplementary material for: The distribution of manta rays in the western North Atlantic Ocean off the eastern United States
Source: Sci Rep. 2022 Apr 21;12:6544. doi: 10.1038/s41598-022-10482-8 (PMC9023537; doi:10.1038/s41598-022-10482-8)
Supplement: Supplementary file 3 — Supplementary Information 2. [file 41598_2022_10482_MOESM3_ESM.docx]

**Supplemental File:** Farmer et al. (2021) The distribution of giant manta rays in the western North Atlantic Ocean off the eastern United States. Sci. Reports.

Distance Function Model Fitting

*SEFSC Surveys*

**
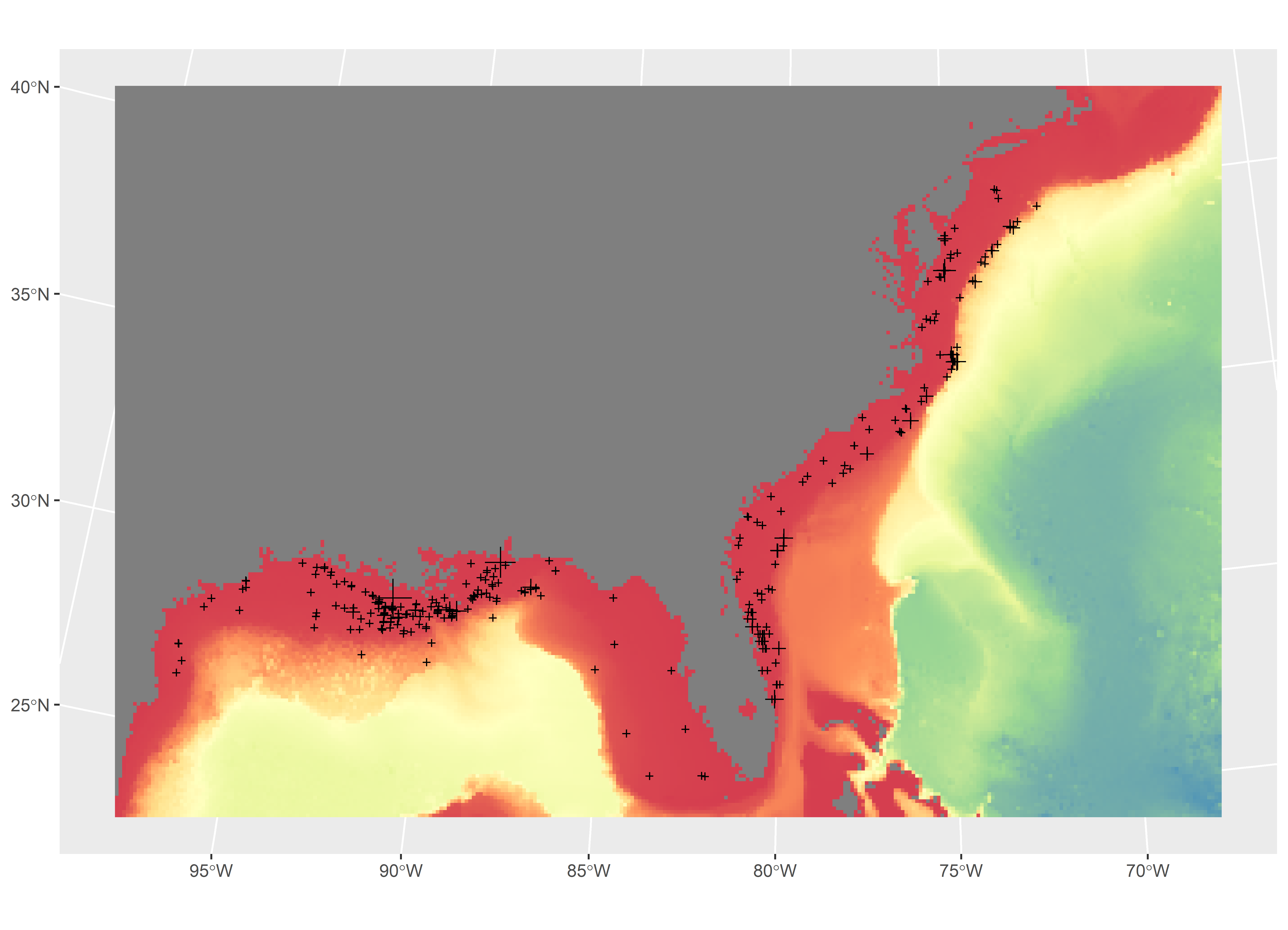
**

**Figure S1.** Sightings from SEFSC surveys relative to bathymetry. Size scaled to number of individuals observed. Map generated in R v4.1.2 (https://cran.r-project.org/bin/windows/base/).


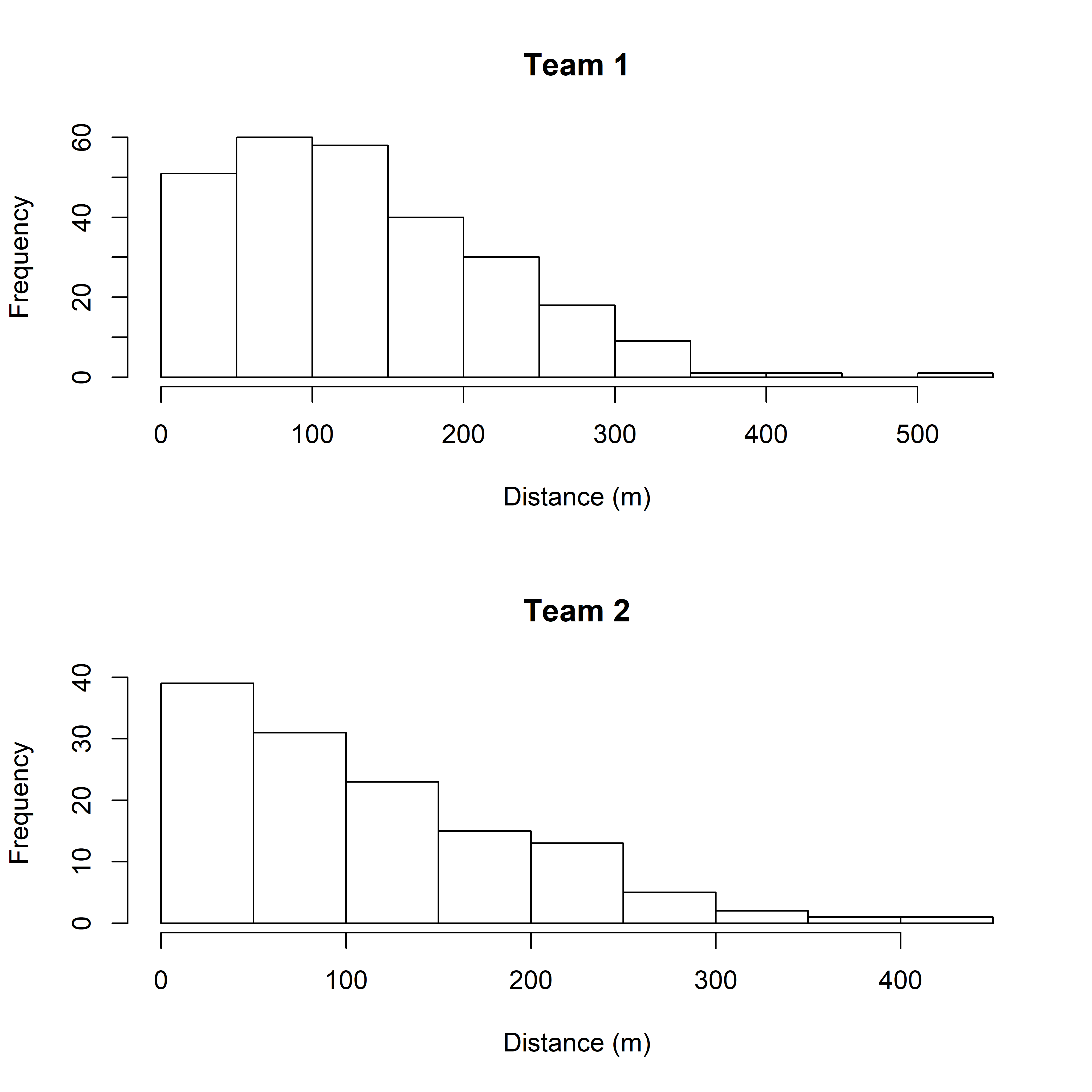


**Figure S2.** SEFSC survey sightings distance histograms for Team 1 and Team 2.

**Table S1.** MCDS model outputs, with selected function in bold.

| **Formula** | **AIC** | **CVM p-value** | **P_a** | **P_a (SE)** | **Delta_AIC** |
| --- | --- | --- | --- | --- | --- |
| **~CloudCover** | **3471.91** | **0.906165** | **0.599822** | **0.032163** | **0** |
| ~1 | 3472.523 | 0.820415 | 0.589046 | 0.033932 | 0.612917 |
| ~Haze+CloudCover | 3473.146 | 0.89357 | 0.603023 | 0.03179 | 1.235486 |
| ~SeaState | 3473.265 | 0.877158 | 0.590794 | 0.033357 | 1.355334 |
| ~g+CloudCover | 3473.423 | 0.895518 | 0.601569 | 0.031885 | 1.512541 |
| ~SeaState+CloudCover | 3473.438 | 0.884026 | 0.600767 | 0.031928 | 1.527394 |
| ~g | 3473.445 | 0.883211 | 0.590866 | 0.033395 | 1.535328 |
| ~SunPen+CloudCover | 3473.647 | 0.913276 | 0.602248 | 0.031964 | 1.736823 |

AIC: Akaike Information Criterion, CVM p-value: Cramer Von-Mises Test p-value, P_a: Estimated detection probability within the truncation distance, P_a (SE): standard error in P_a, Delta_AIC: change in AIC from previous row.

**Table S2.** MRDS Model Outputs, with selected function in bold.

| **MRDS Model** | **AIC** |
| --- | --- |
| ~distance*observer+Haze+Glare+SS | 4134.323 |
| ~distance*observer+Haze+Glare | 4134.771 |
| **~distance*observer+Glare** | **4134.967** |
| ~distance*observer+SeaState+Haze+Glare | 4135.285 |
| ~distance*observer+Haze+g+Glare+SS | 4135.77 |
| ~distance*observer+Glare+SS | 4135.803 |

Results of ddf.gof (Goodness of fit testing):

- MR total chi-square = 17.81 P = 0.16488 with 13 degrees of freedom
- Total chi-square = 26.932 P = 0.080289 with 18 degrees of freedom


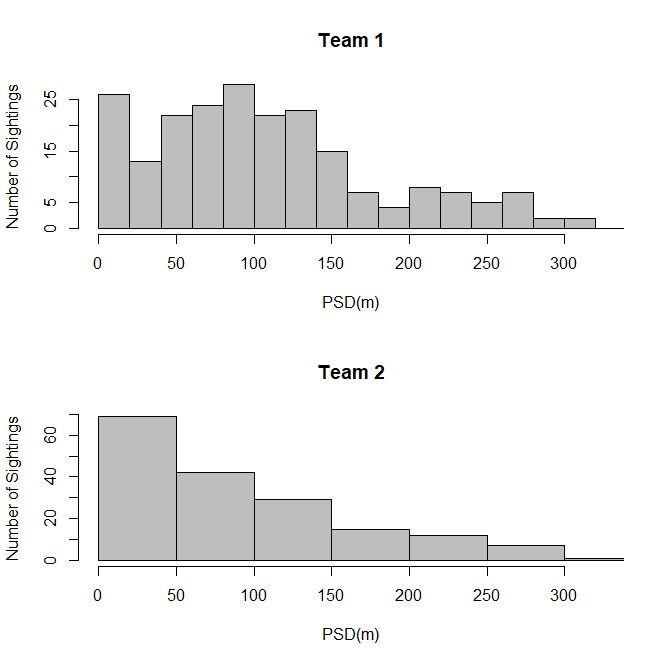


**Figure S3.** Perpendicular sighting distance (PSD) for dual-observer team mark-recapture sampling on combined SEFSC surveys.


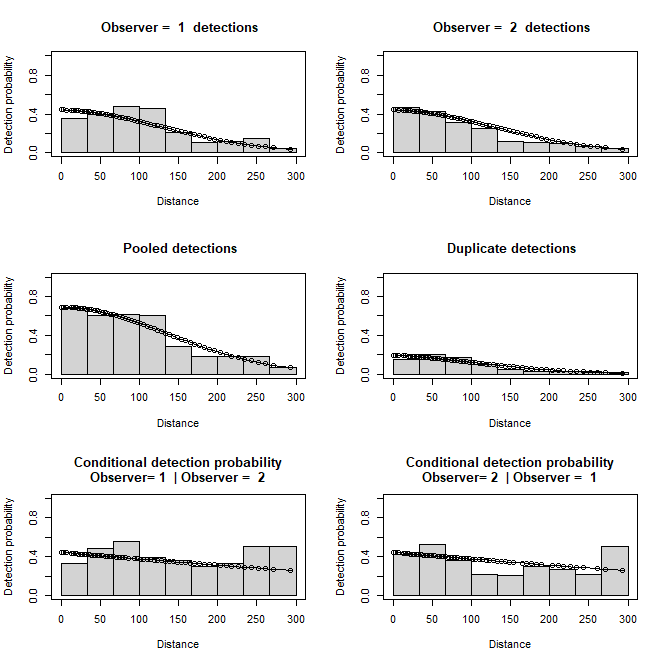


**Figure S4.** Detection probabilities vs. distance and conditional detection probabilities for dual-observer team mark-recapture sampling on combined SEFSC surveys.


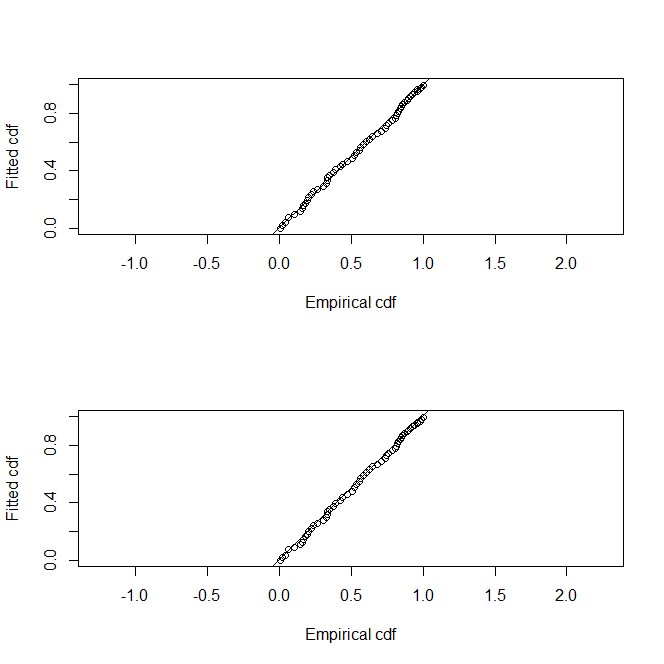


**Figure S5.** Q-Q plot for distance sampling function for combined SEFSC surveys.

mrds.formula <- "~distance * observer + Glare"

mcds.formula <- "~CloudCover"

#Set best model

mrds.best <-mrds::ddf(method='io',dsmodel=~mcds(key= ds.key, formula = mcds.formula),

mrmodel=~glm(link='logit',formula= mrds.formula), data=sight,

meta.data=list(binned=F, width = RT.distance))

summary(mrds.best)

Summary for io.fi object

Number of observations : 314

Number seen by primary : 213

Number seen by secondary : 174

Number seen by both : 73

AIC : 663.0573

Conditional detection function parameters:

estimate se

(Intercept) -0.930701017 0.472932805

distance 0.003431083 0.003663183

observer 0.100996057 0.230822783

Glare 0.839444818 0.308622813

distance:observer -0.004173455 0.001904360

Estimate SE CV

Average primary p(0) 0.4104155 0.06516574 0.1587799

Average secondary p(0) 0.4340387 0.06451705 0.1486435

Average combined p(0) 0.6563283 0.06961203 0.1060628

Summary for ds object

Number of observations : 314

Distance range : 0 - 300

AIC : 3471.91

Detection function:

Hazard-rate key function

Detection function parameters

Scale coefficient(s):

estimate se

(Intercept) 5.08359222 0.11249675

CloudCover -0.06728108 0.04479493

Shape coefficient(s):

estimate se

(Intercept) 1.053164 0.1812558

Estimate SE CV

Average p 0.5998223 0.03216317 0.05362117

Summary for io object

Total AIC value : 4134.967

Estimate SE CV

Average p 0.3936804 0.04689548 0.1191207

N in covered region 797.6013305 101.44847473 0.1271920

*NARWC Surveys*


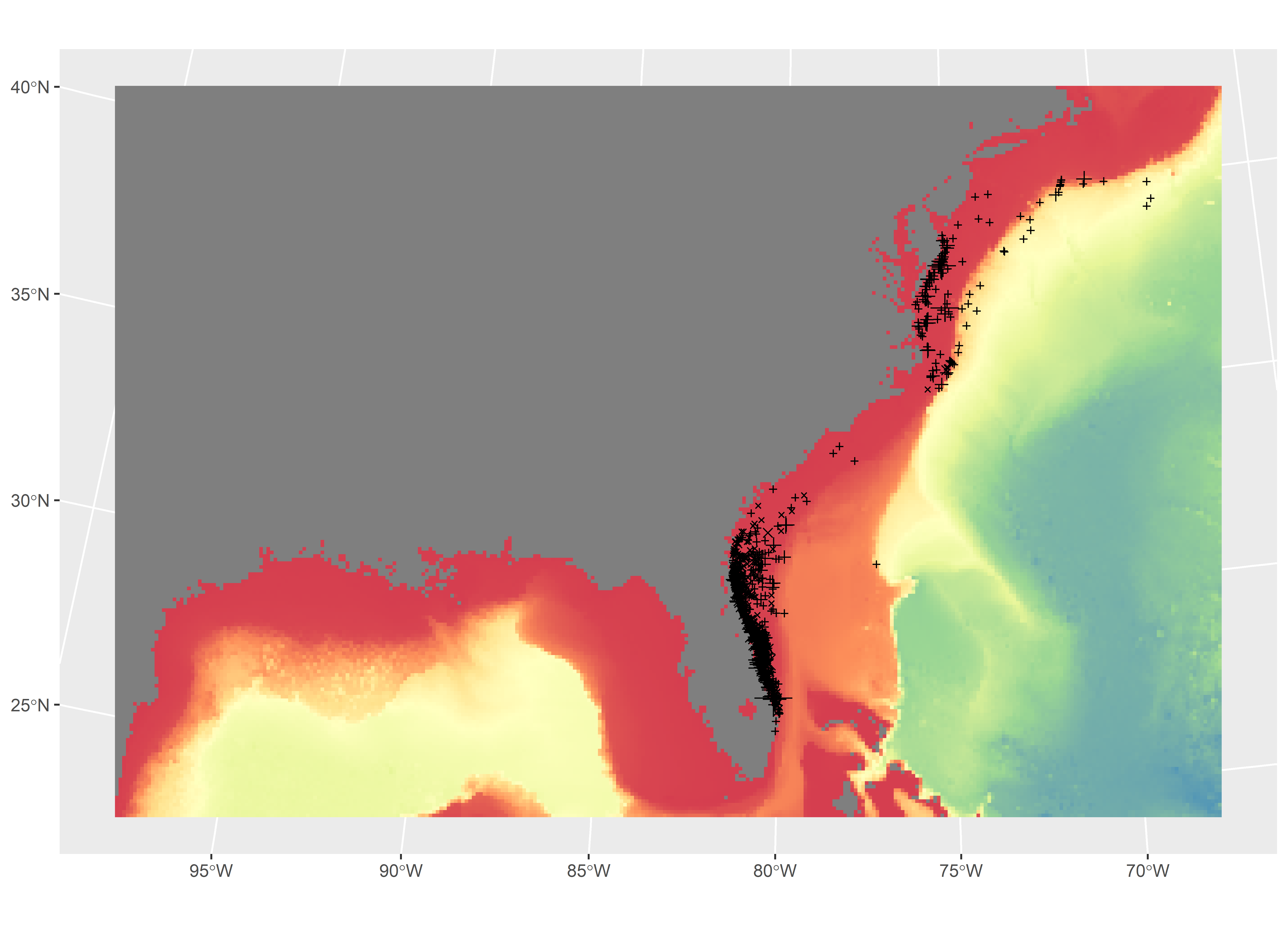


**Figure S6.** Sightings from NARWC surveys relative to bathymetry. Size scaled to number of individuals observed. Map generated in R v4.1.2 (https://cran.r-project.org/bin/windows/base/).


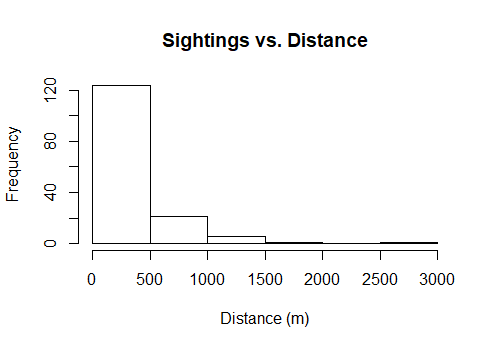


**Figure S7.** Sightings vs. perpendicular distance for on-transect distance-sampling NARWC surveys on the Skymaster platform.


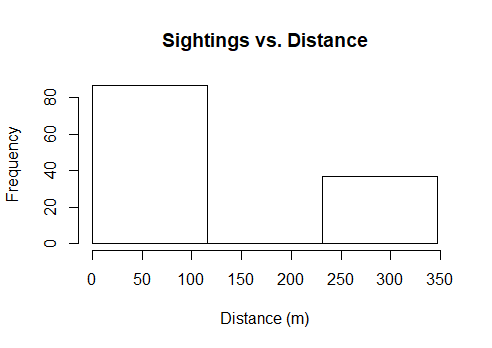


**Figure S8.** Right-truncated (80% Quartile = 348 m) sightings vs. perpendicular distance for on-transect distance-sampling NARWC surveys on the Skymaster platform.

quantile(narwc.sightings$distance,c(0.75,0.8,0.85,0.9,0.975))

#distance doubles between 80%-85%, seems unrealistic; SEFSC survey RT.distance was 300, so going with 348 m here.

75% 80% 85% 90% 97.5%

347.25 347.25 694.50 694.50 1157.50

**Table S3.** Distance-sampling function outputs for NARWC surveys on Skymaster platform, with selected function in bold.

| **Distance Model** | **Key** | **Adjustment** | **Order** | **Covariates** | **df** | **AIC** |
| --- | --- | --- | --- | --- | --- | --- |
| manta.hn.cos | hn | cos | 1 |  | 1 | 153.154 |
| manta.hn.poly | hn | poly | 1 |  | 1 | 153.154 |
| manta.hr.cos | hr | cos | 1 |  | 2 | 155.144 |
| manta.hr.poly | hr | poly | 1 |  | 2 | 155.144 |
| **manta.hn.ss** | **hn** | **cos** | **1** | **SeaState** | **5** | **155.07** |
| manta.hn.ss2 | hn | cos | 2 | SeaState | 6 | 157.07 |
| manta.hr.ss | hr | cos | 1 | SeaState | 6 | 183.829 |
| manta.hr.p | hr | poly | 1 |  | 6 | 156.79 |
| manta.hn.ss.cloud | hn | cos | 1 | SeaState+CloudCover | 8 | 160.313 |
| manta.hn.ss.glare | hn | cos | 1 | SeaState+Glare | 9 | 157.084 |
| manta.hn.ss.cloud.glare | hn | cos | 1 | SeaState+CloudCover+Glare | 12 | 151.668 |

hn: half-normal, hr: hazard rate, cos: cosine, poly: polynomial

Model with SeaState only selected. Although including both CloudCover and Glare improved the distance function, these variables were not available on most non-distance sampled Skymaster flights; SeaState was always available and appeared to make a difference (see below).


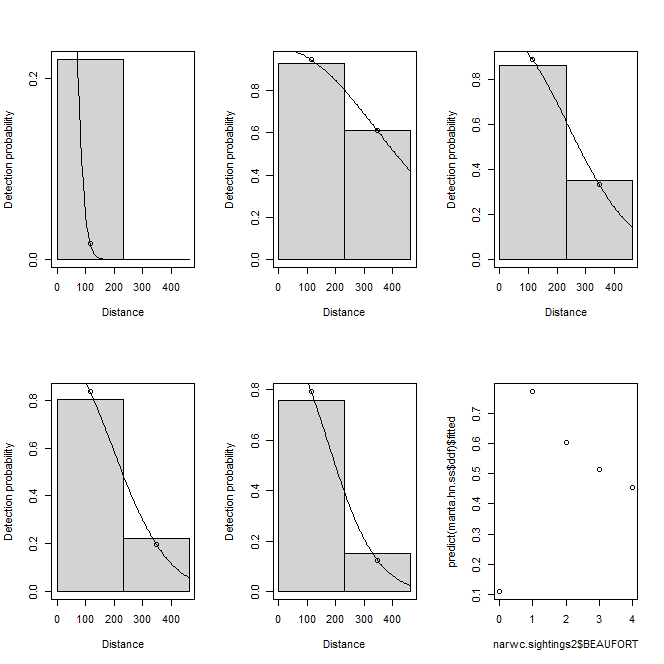


**Figure S9.** Final NARWC Skymaster distance-sampling functional fits to Beaufort Sea States 0-4. Note the reduced detection probability with flat water and gradual reduction in sighting probability at distance as sea states increase from 1.

> manta.hn.ss<-ds(narwc.sightings2, formula=~as.factor(BEAUFORT), key="hn", adjustment="cos")

Columns "distbegin" and "distend" in data: performing a binned analysis...

Model contains covariate term(s): no adjustment terms will be included.

Fitting half-normal key function

AIC= 155.07

No survey area information supplied, only estimating detection function.

> summary(manta.hn.ss)

Summary for distance analysis

Number of observations : 124

Distance range : 0 - 463

Model : Half-normal key function

AIC : 155.0696

Detection function parameters

Scale coefficient(s):

estimate se

(Intercept) 3.705950 400.3919

as.factor(BEAUFORT)1 2.150722 400.6671

as.factor(BEAUFORT)2 1.752356 400.6429

as.factor(BEAUFORT)3 1.556526 400.6270

as.factor(BEAUFORT)4 1.426675 400.6178

Estimate SE CV

Average p 0.5549046 26.99503 48.64806

N in covered region 223.4618524 10870.99465 48.64810

> ddf.gof(manta.hn.ss$ddf) #Cramer-von Mises p>0.05, so adequate fit

Goodness of fit results for ddf object

Chi-square tests

[0,232] (232,463] Total

Observed 8.70000e+01 3.700000e+01 1.240000e+02

Expected 8.70000e+01 3.700000e+01 1.240000e+02

Chisquare 2.77284e-14 6.519922e-14 9.292763e-14

No degrees of freedom for test

**
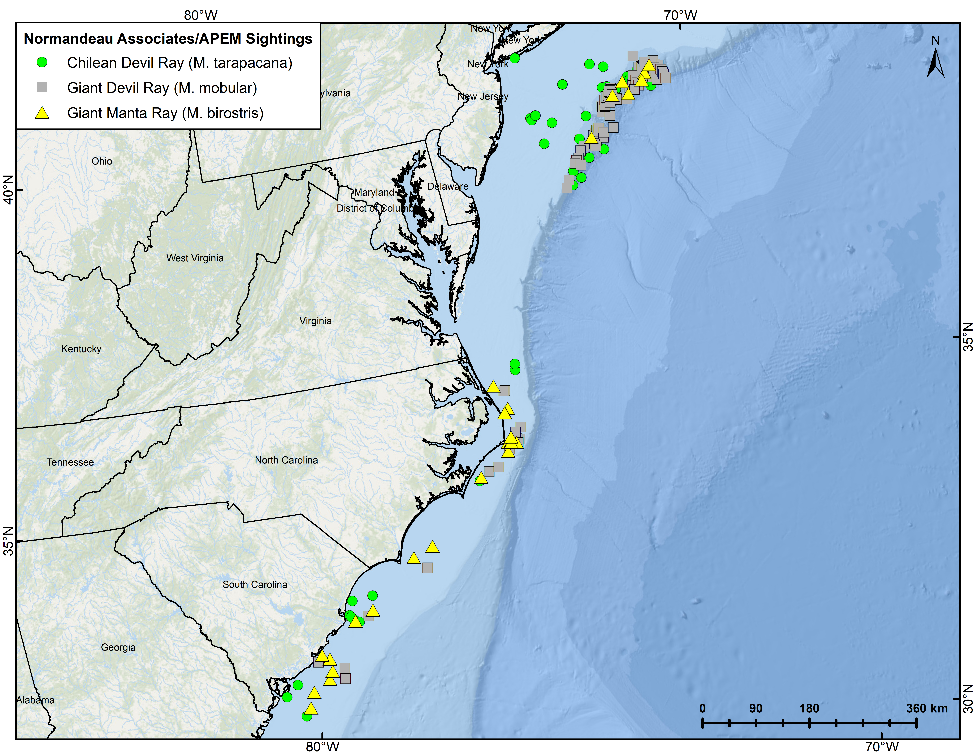
Figure S10.** Sightings of manta and other mobulid rays by Normandeau Associates/APEM surveys. Map generated in ArcMap 10.8.1. Basemap used with permission from ESRI Ocean Basemap and its partners.

**Figure S11.** Time series of Chlorophyll-a satellite measures within study area, 2003 to 2019. Map generated in R v4.1.2 (https://cran.r-project.org/bin/windows/base/).
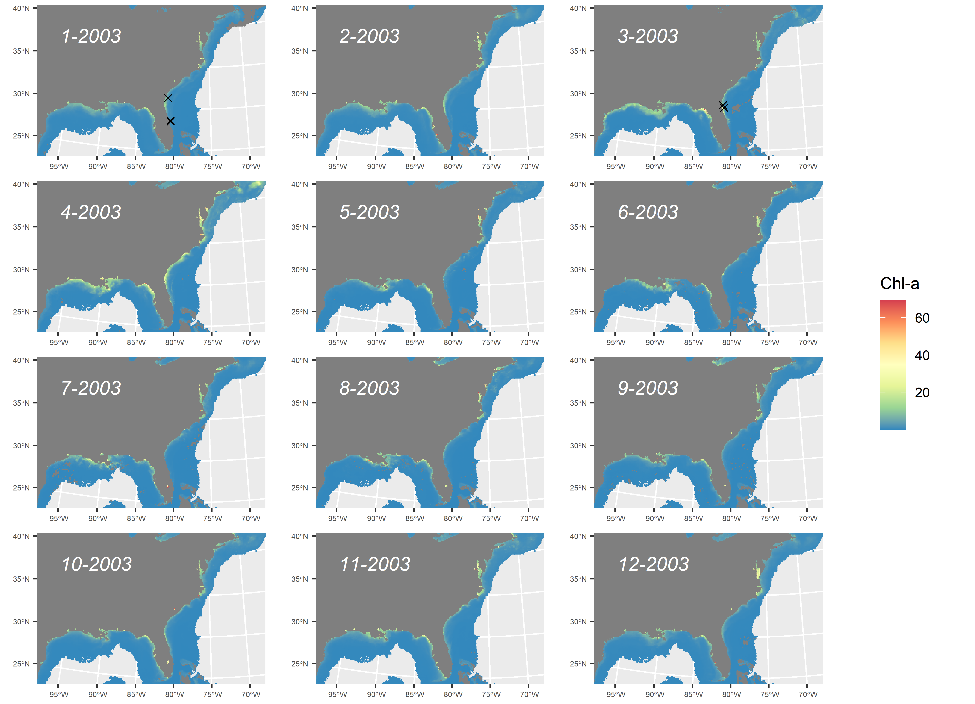

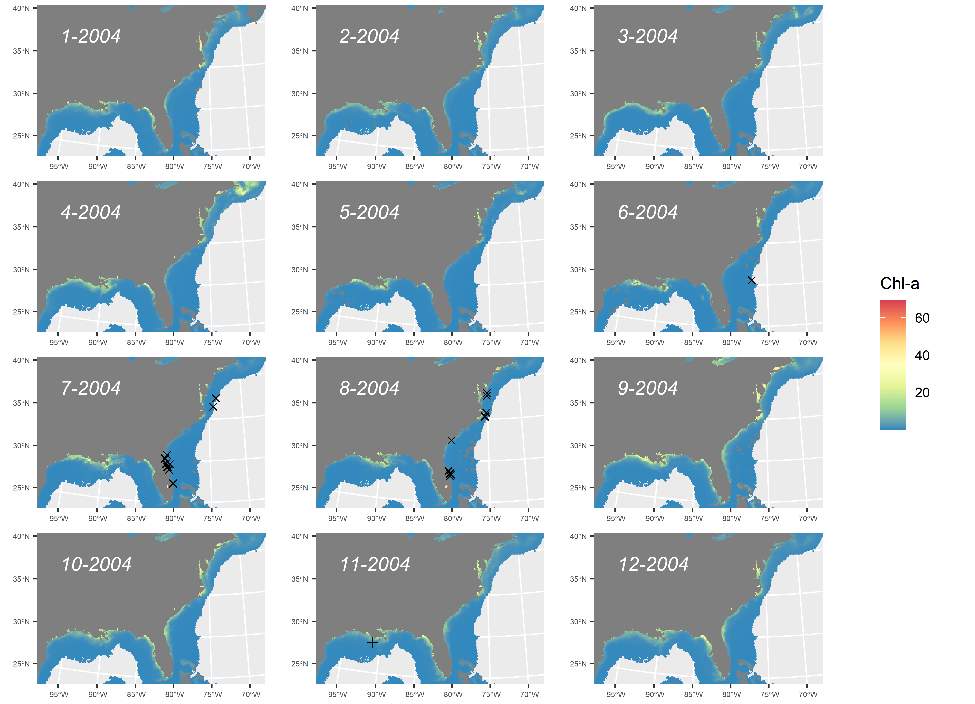

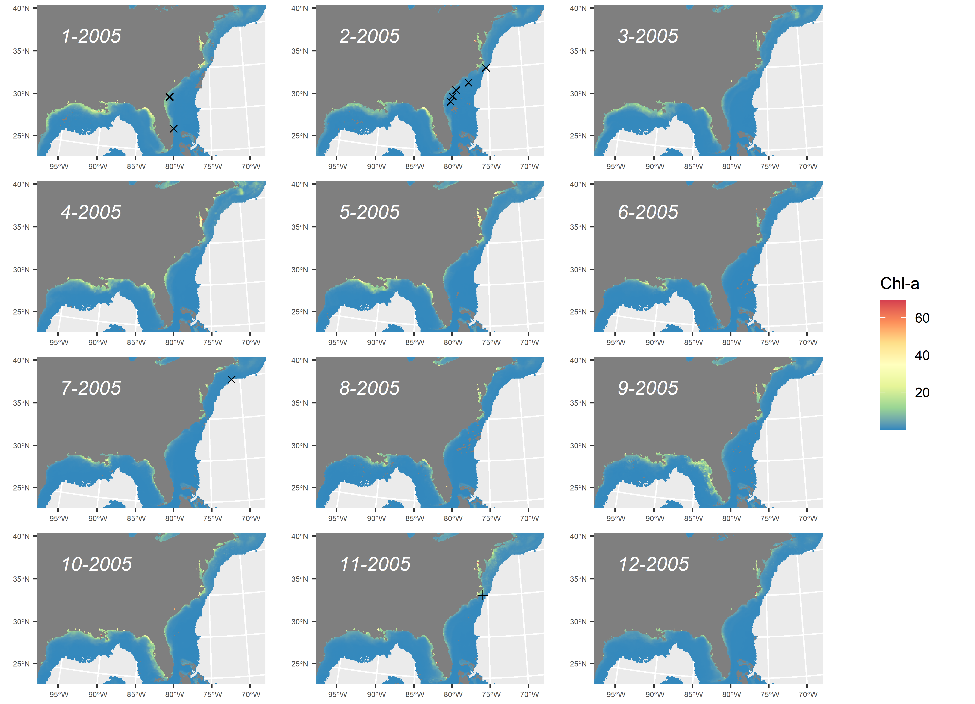

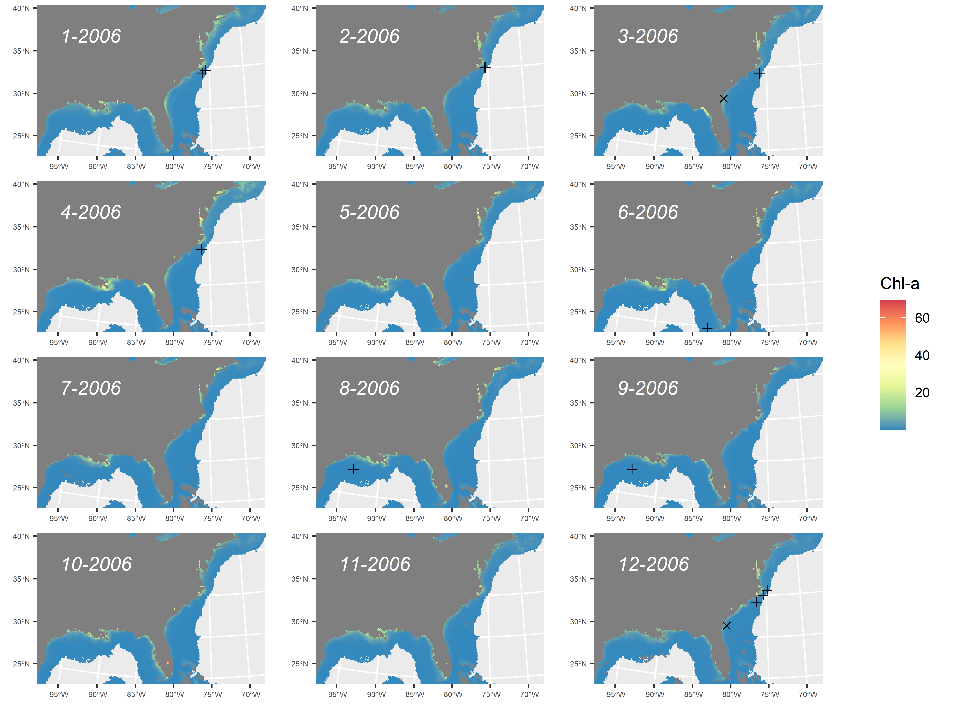

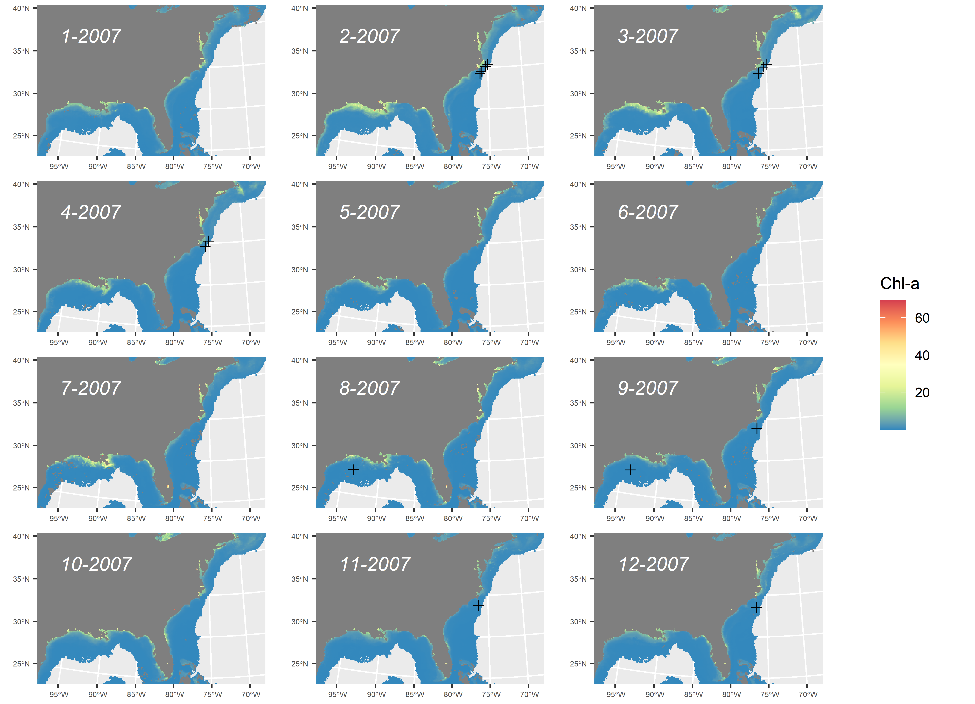

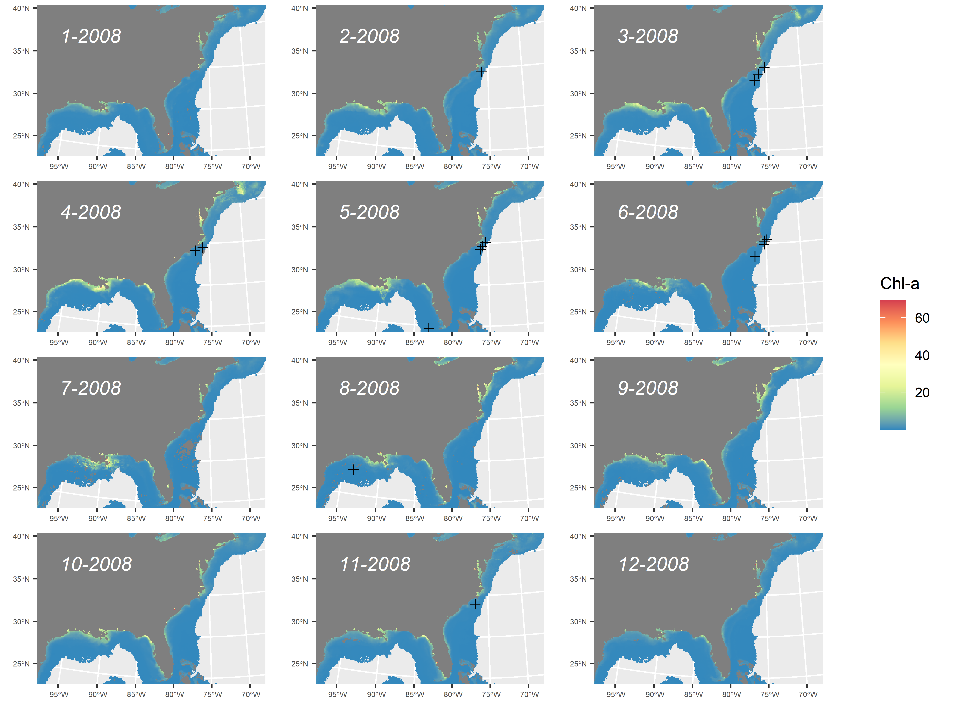

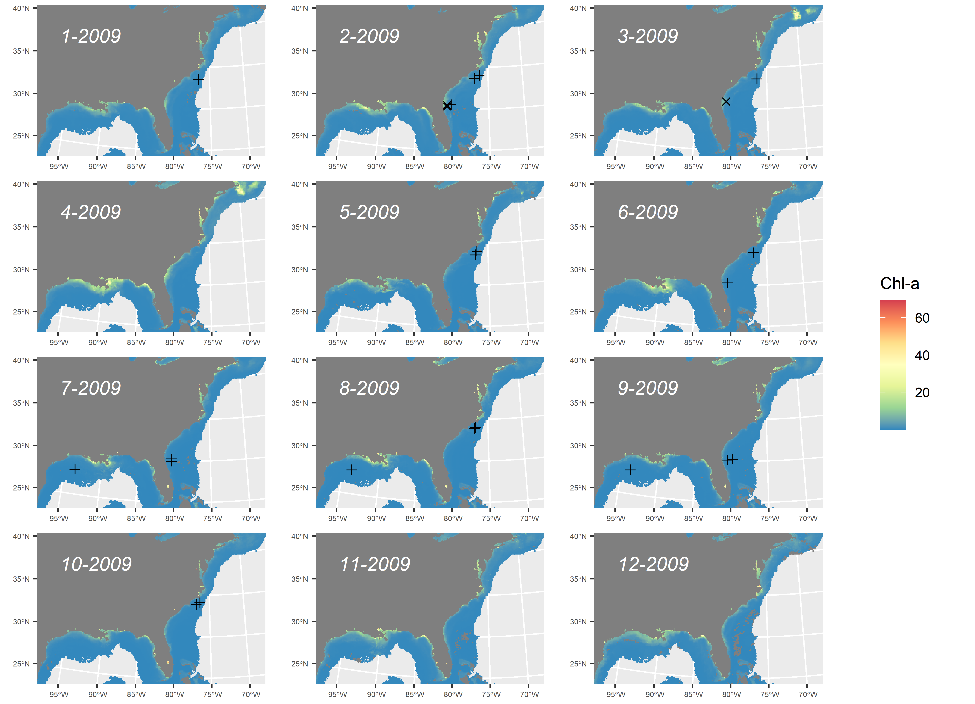

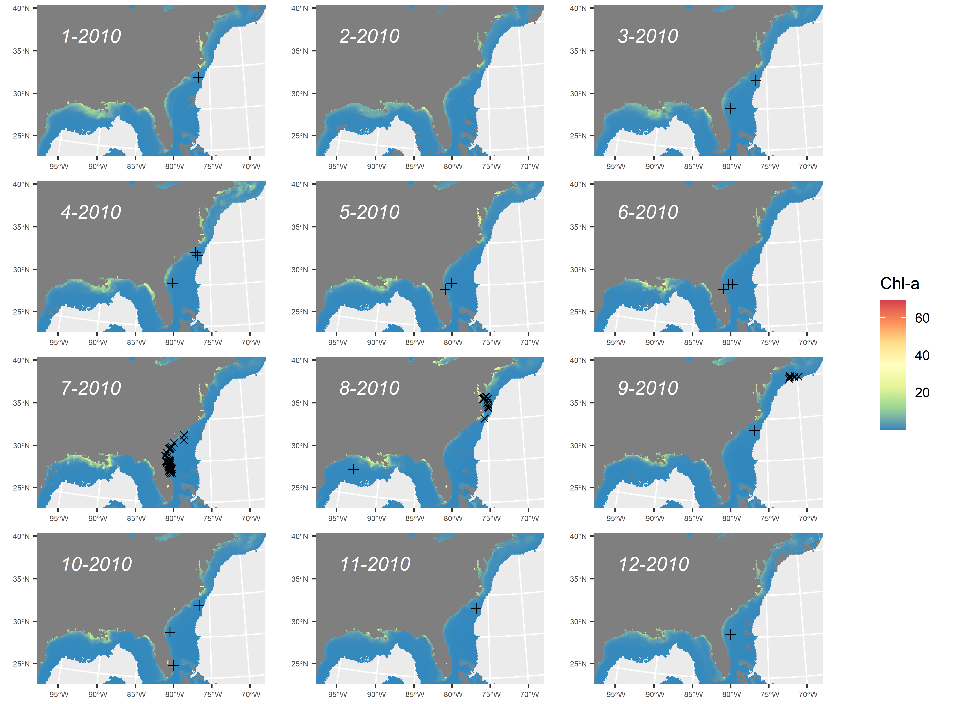

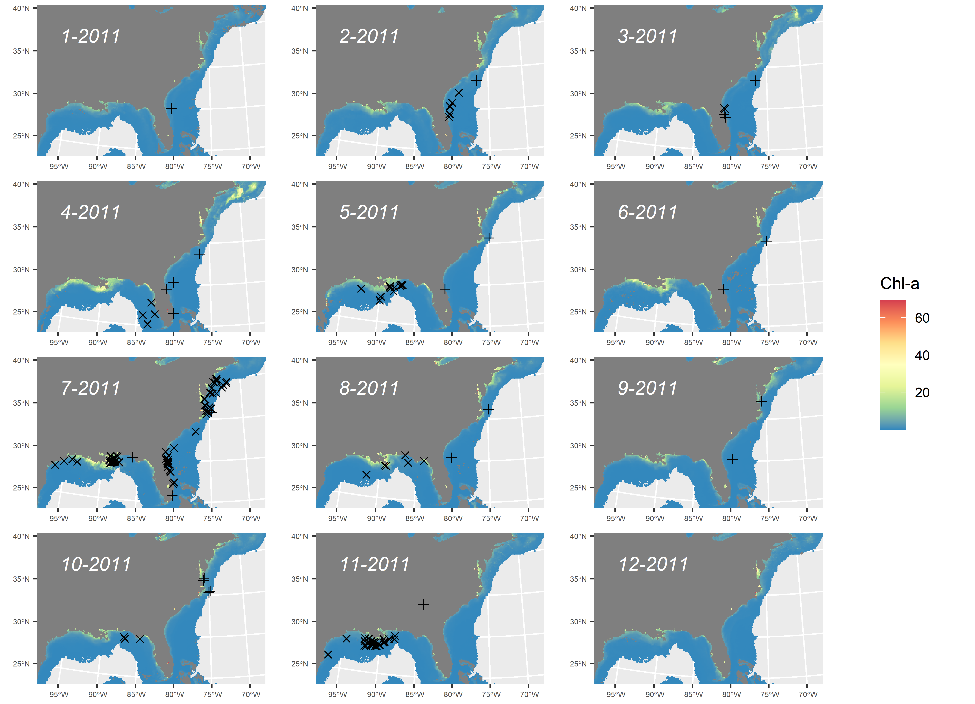

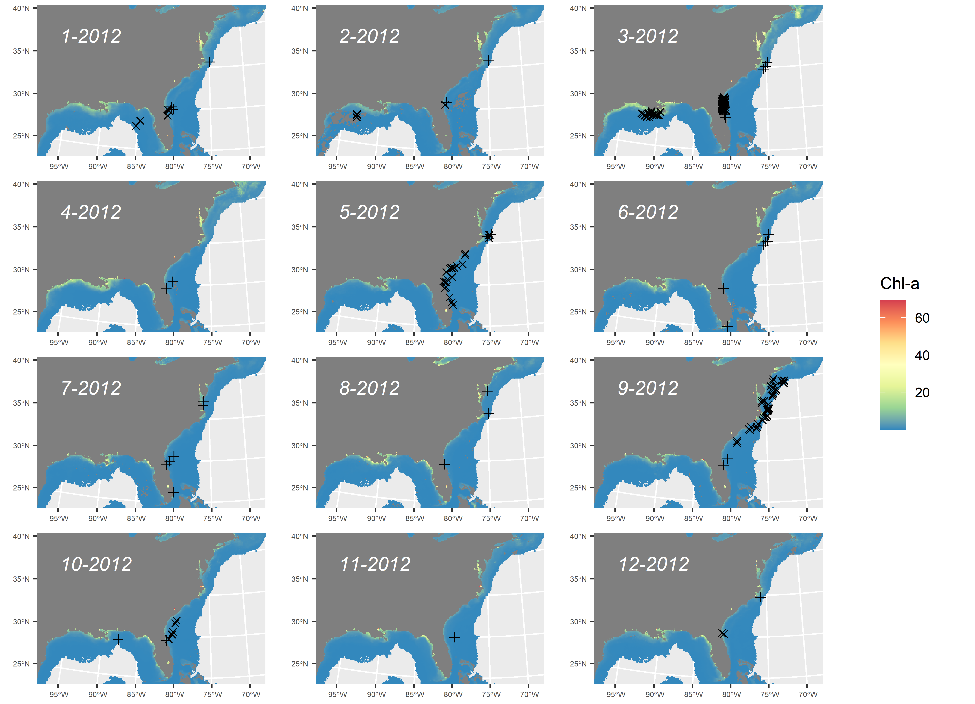

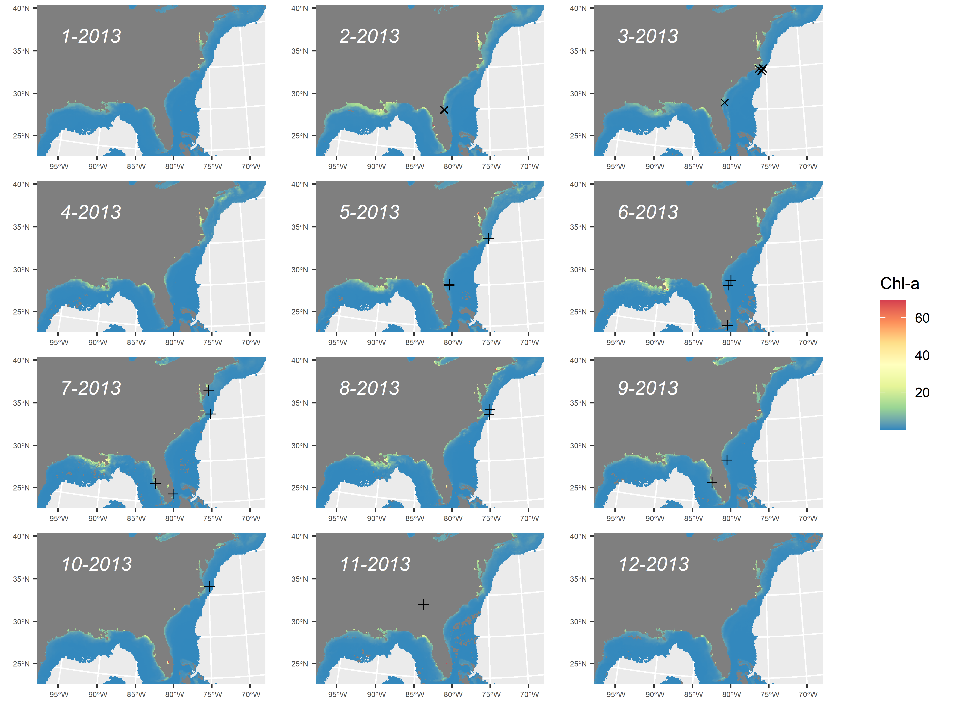

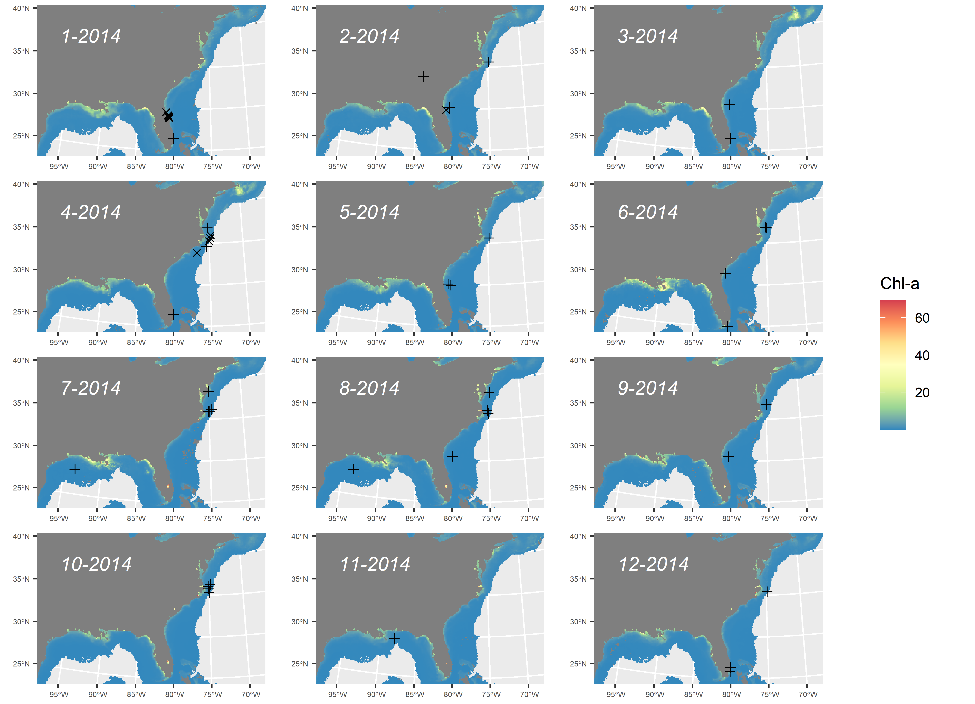

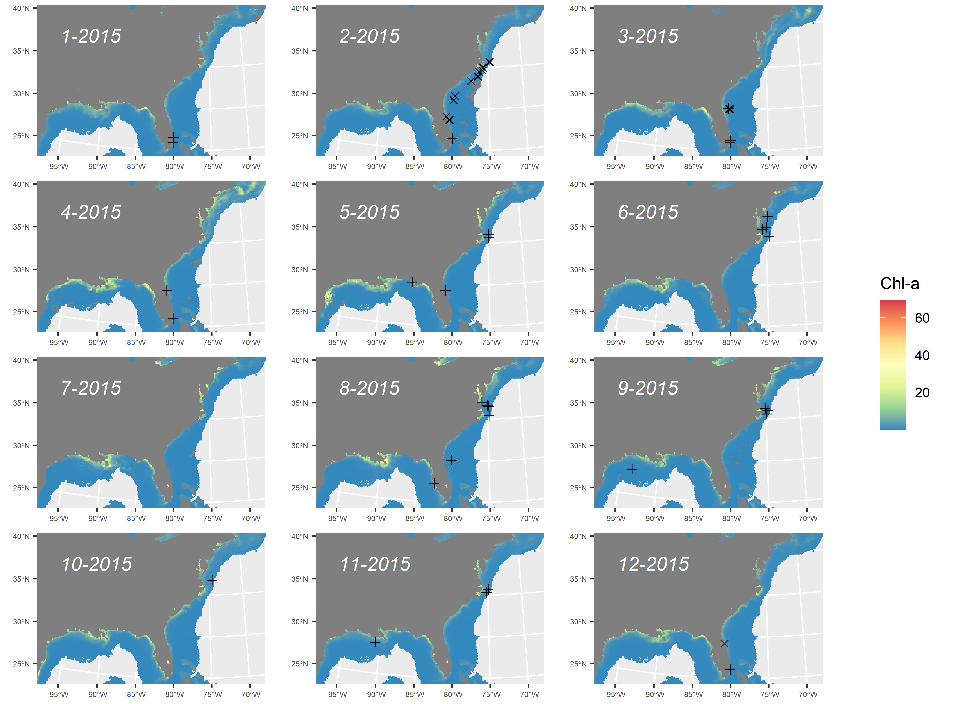

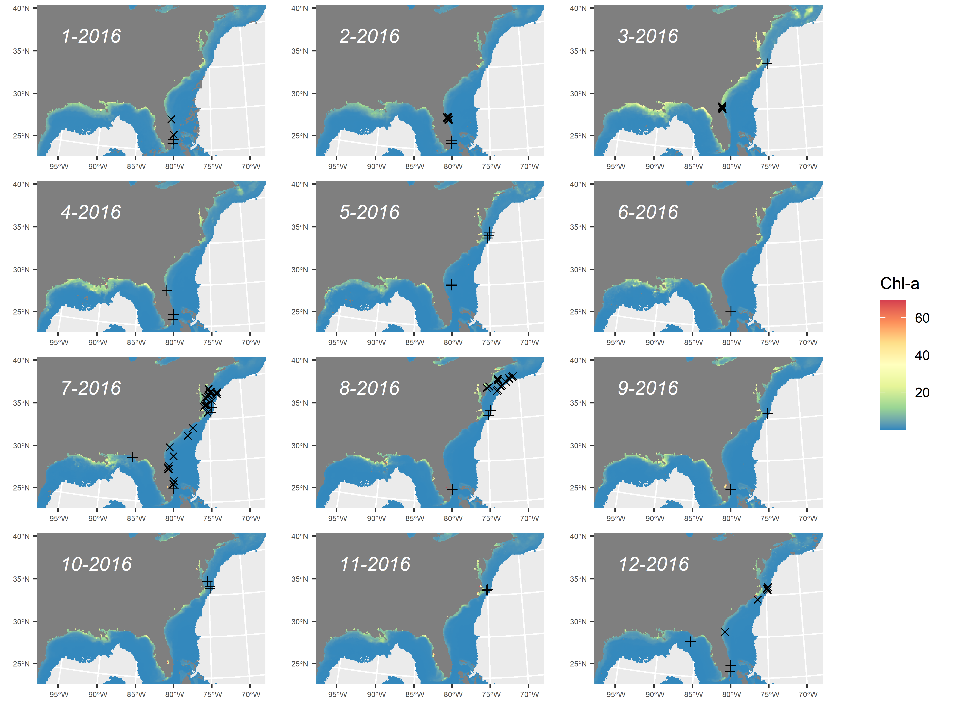

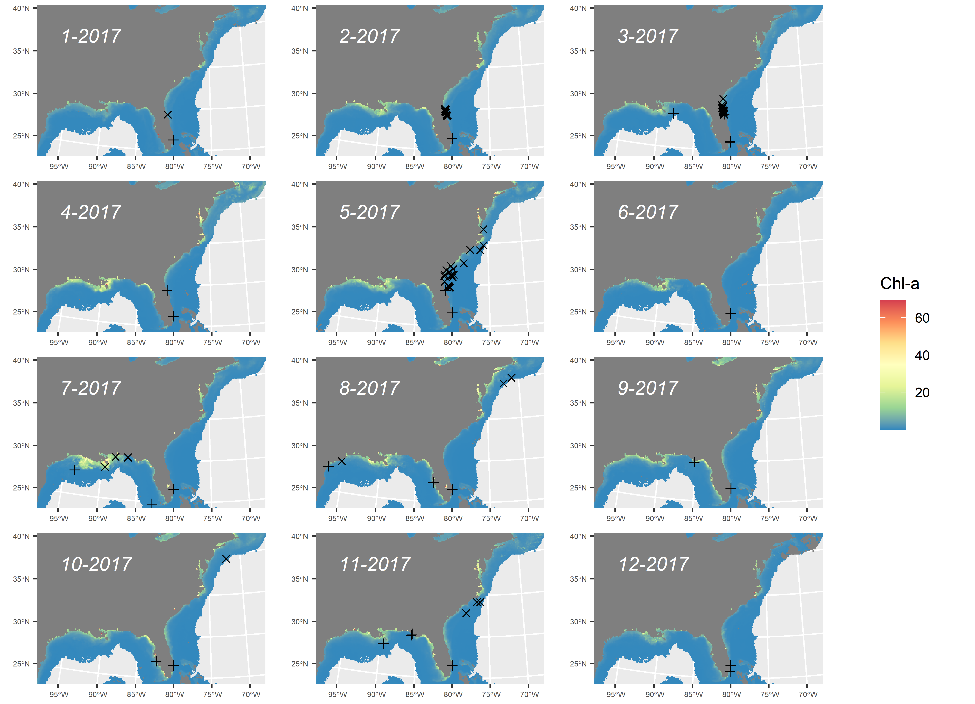

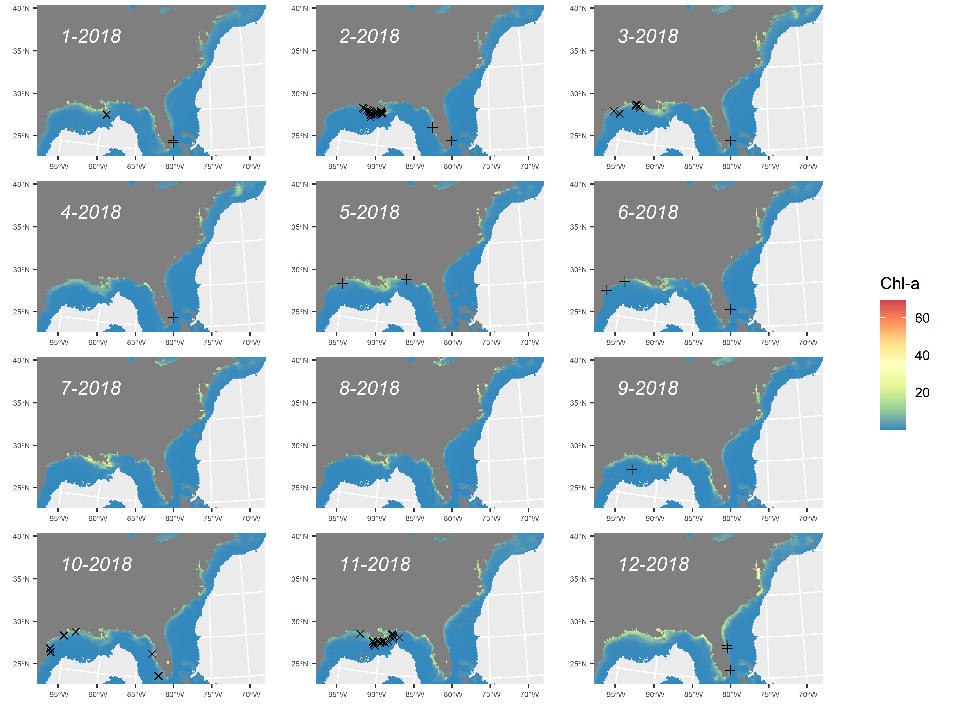

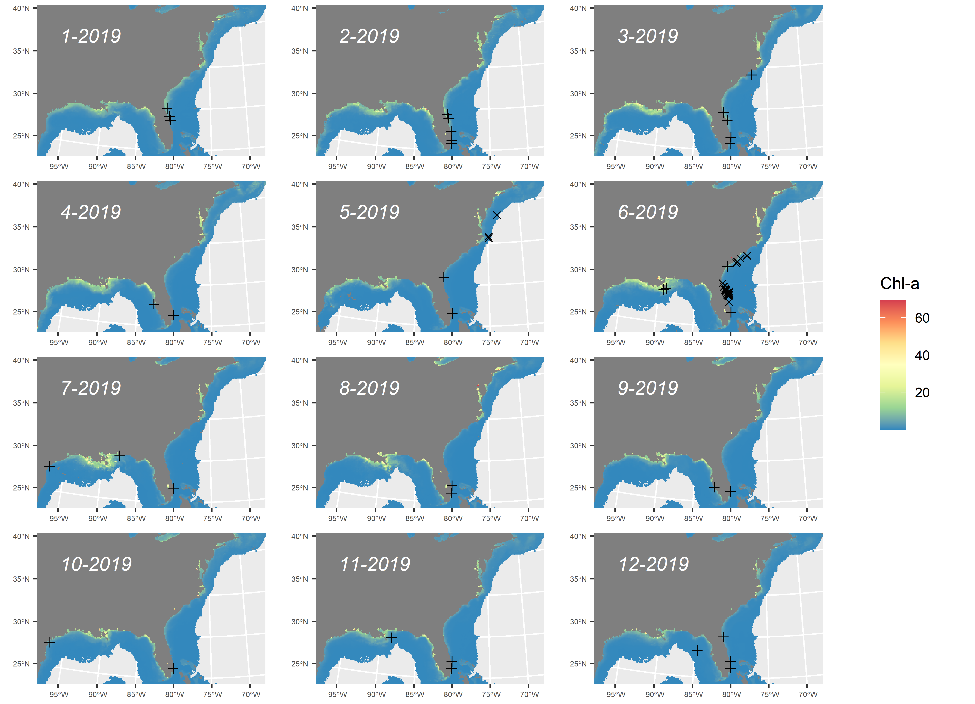


**Figure S12.** Time series of predicted probability of occurrence for manta rays for SEFSC model for monthly average environmental conditions 2003 to 2019 with overlay of internal (X) and external (+) validation points. Map generated in R v4.1.2 (https://cran.r-project.org/bin/windows/base/).
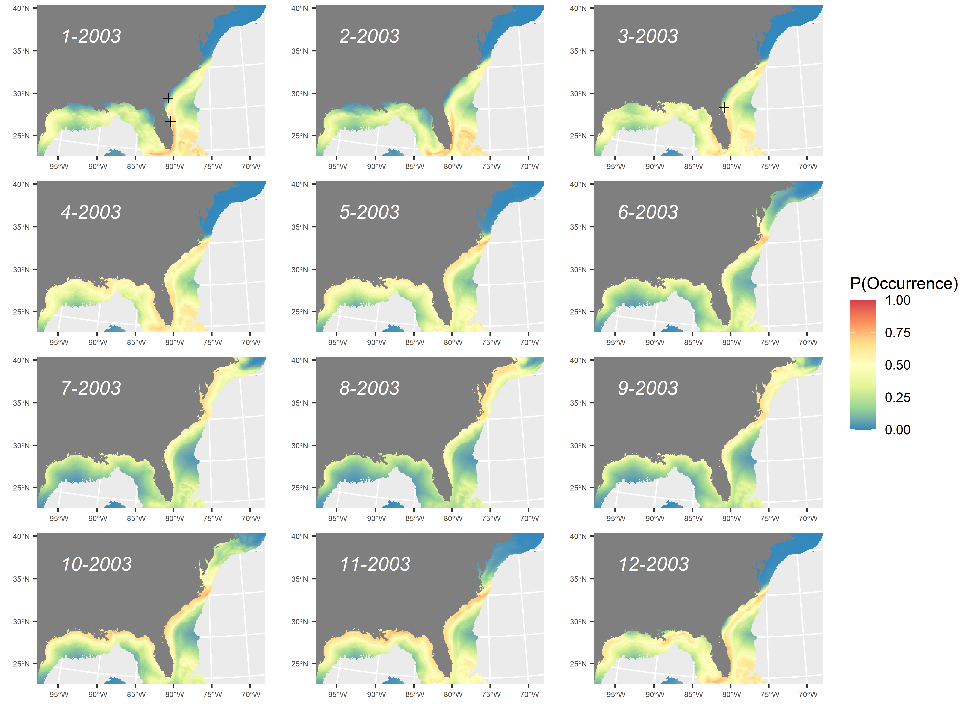

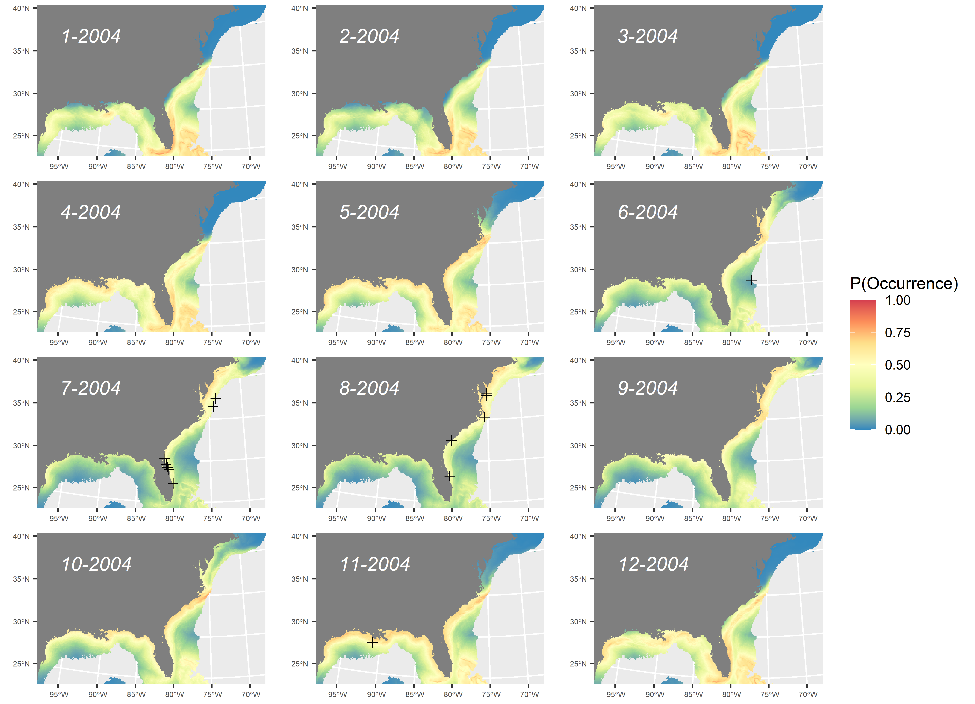

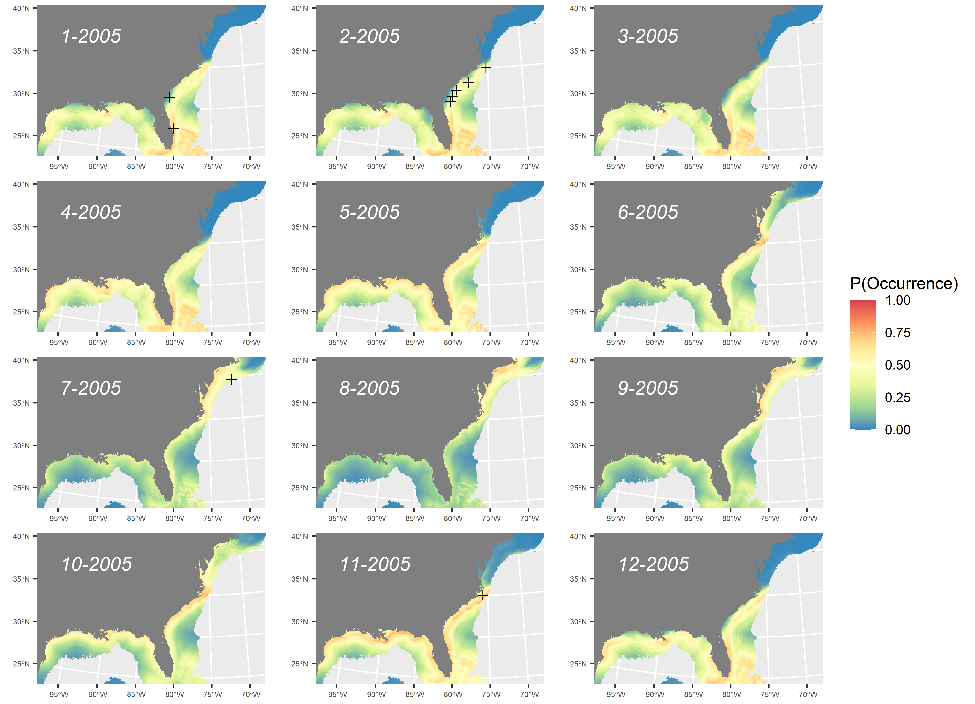

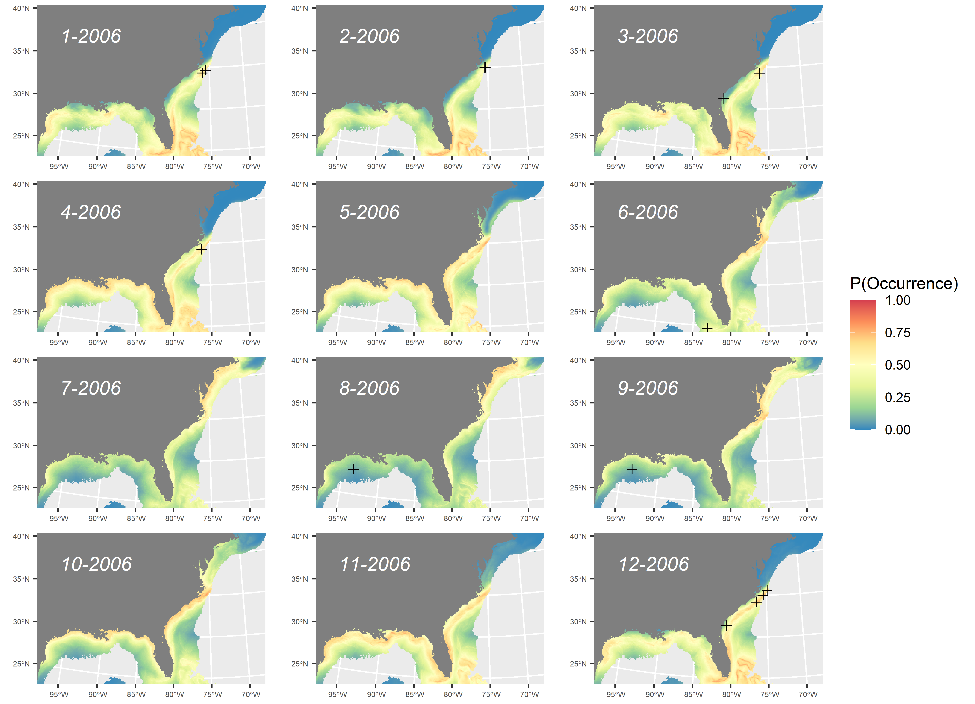

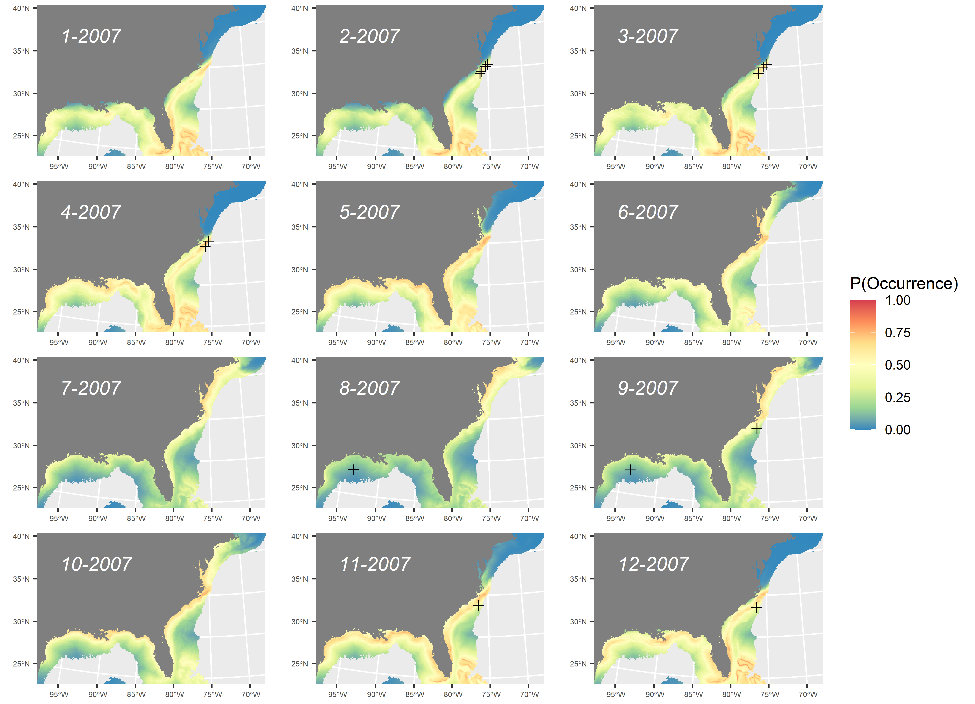

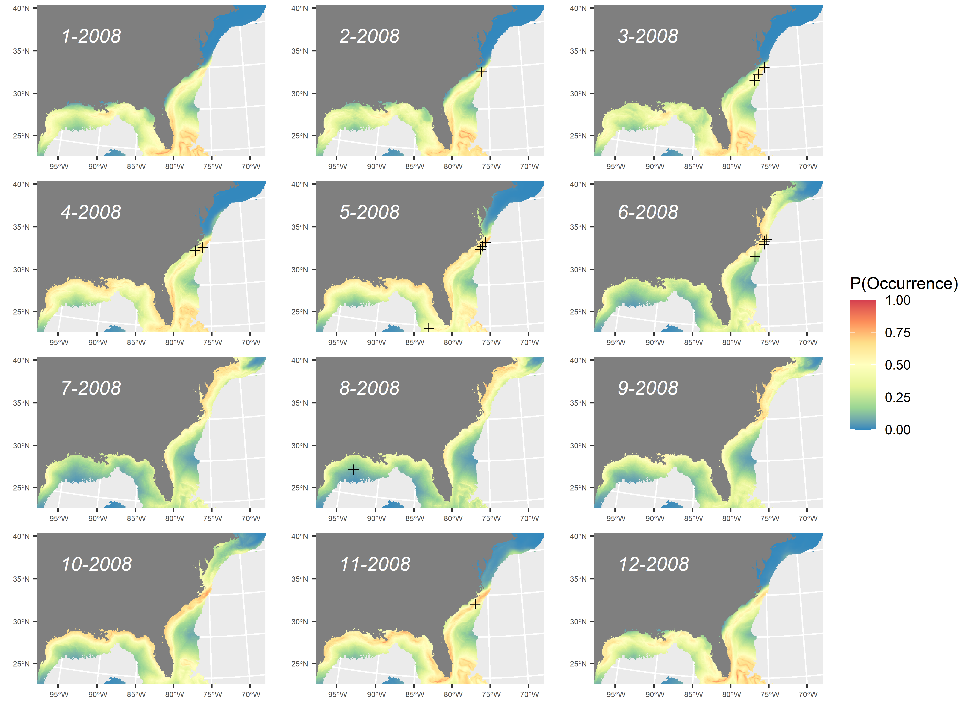

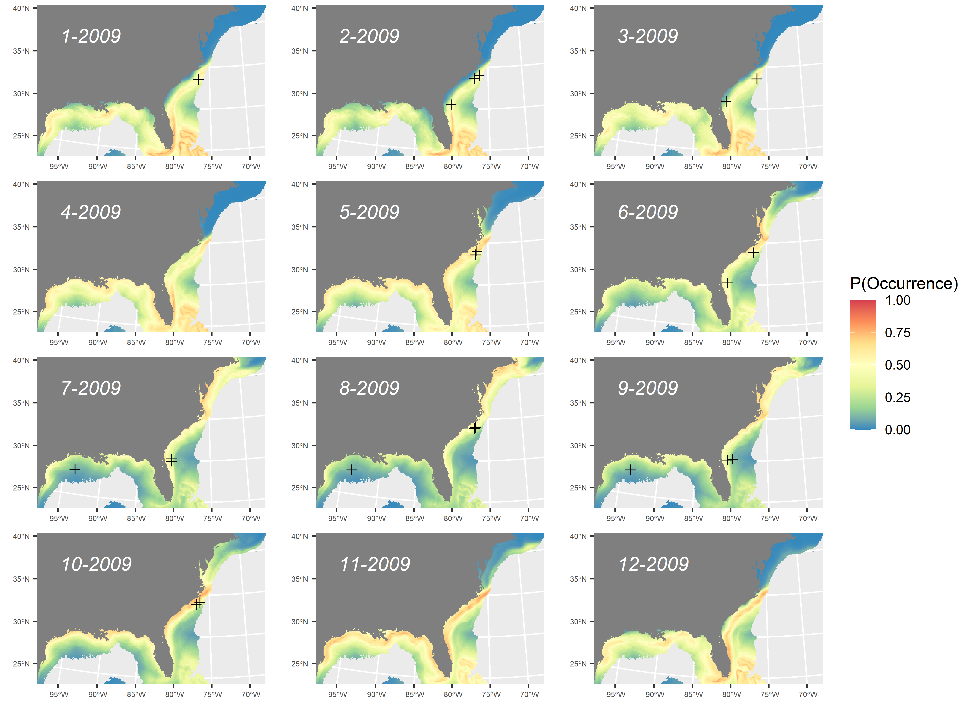

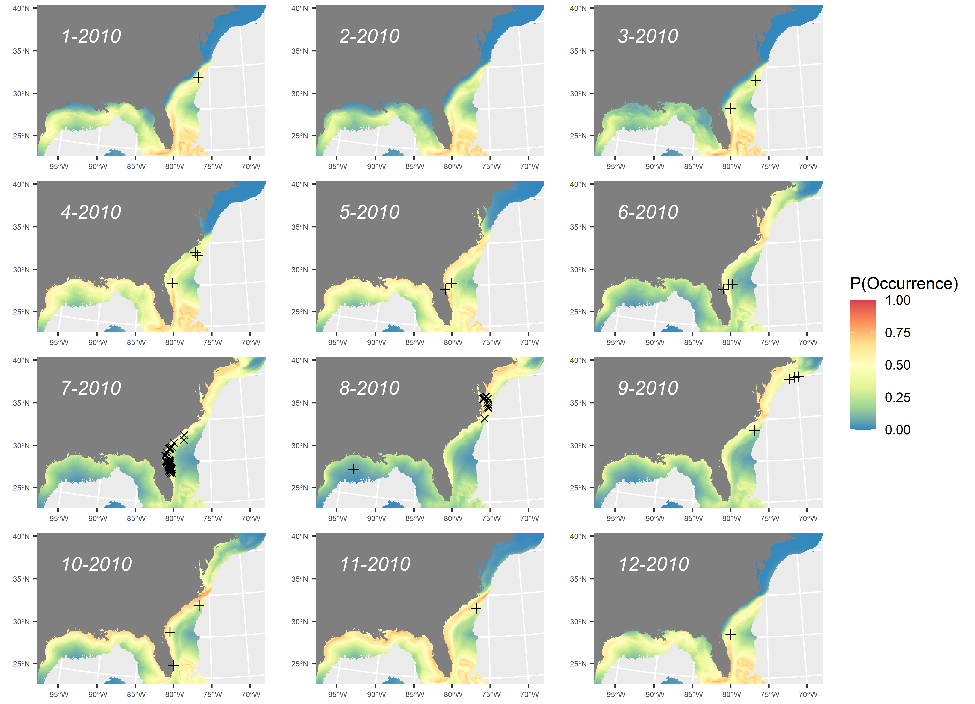

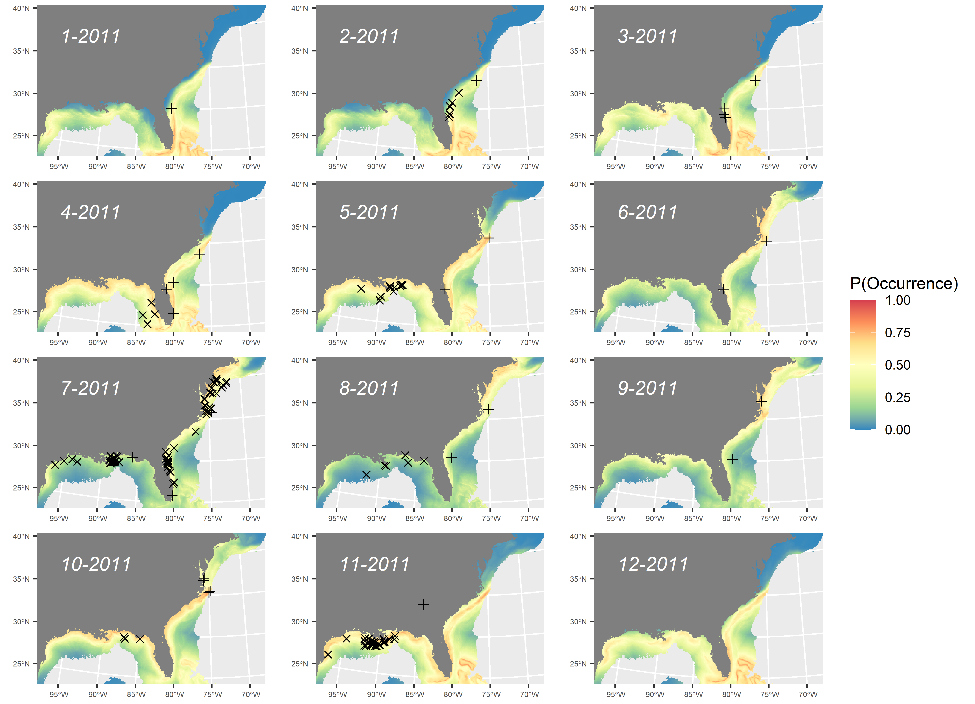

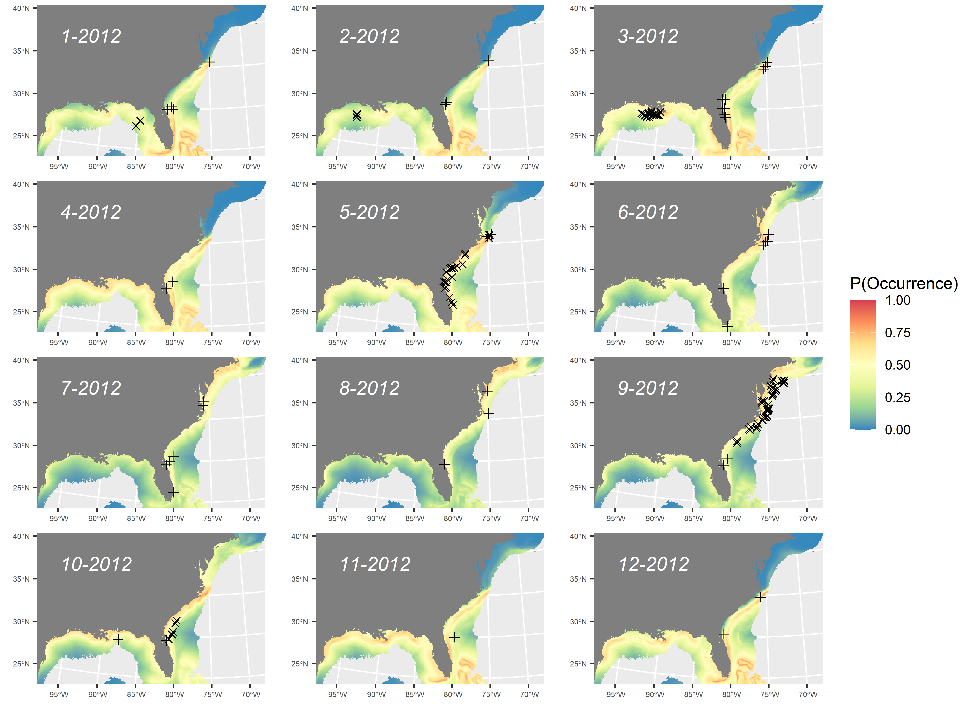

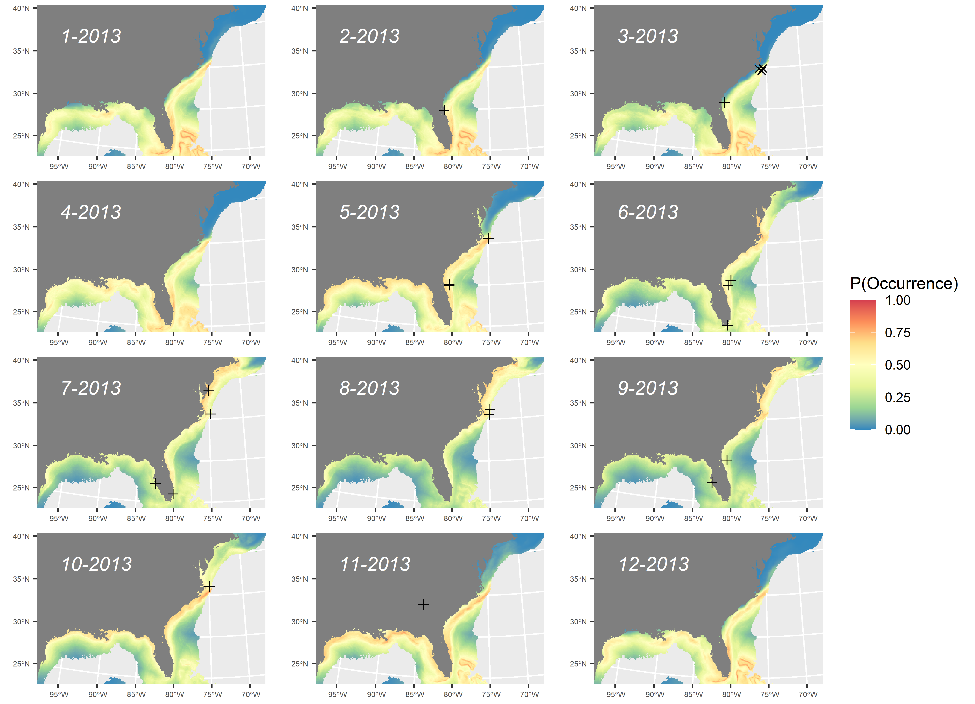

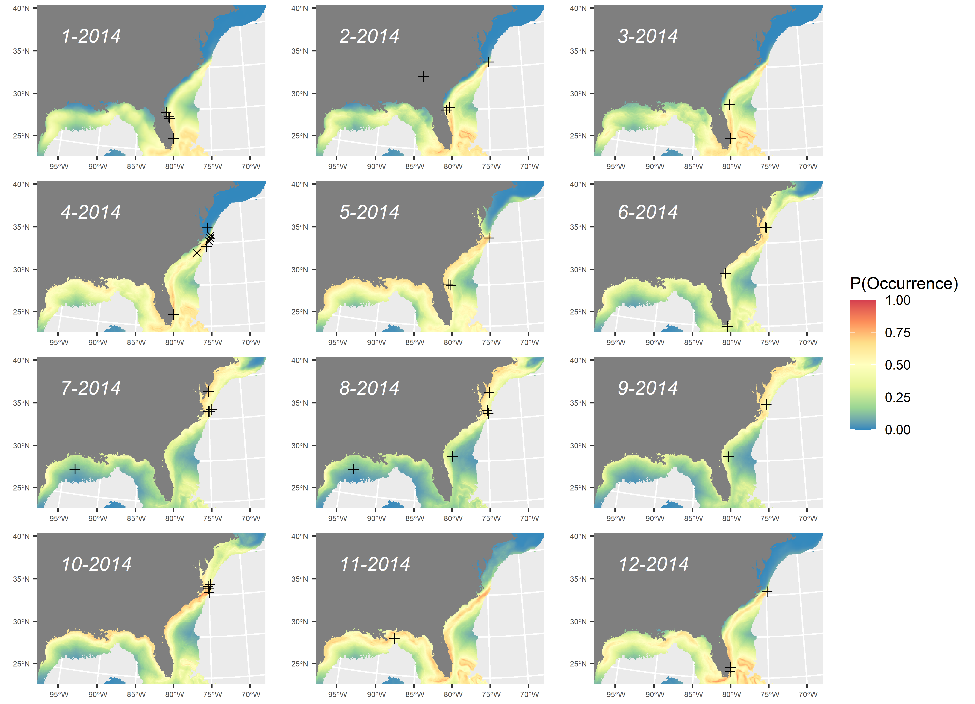

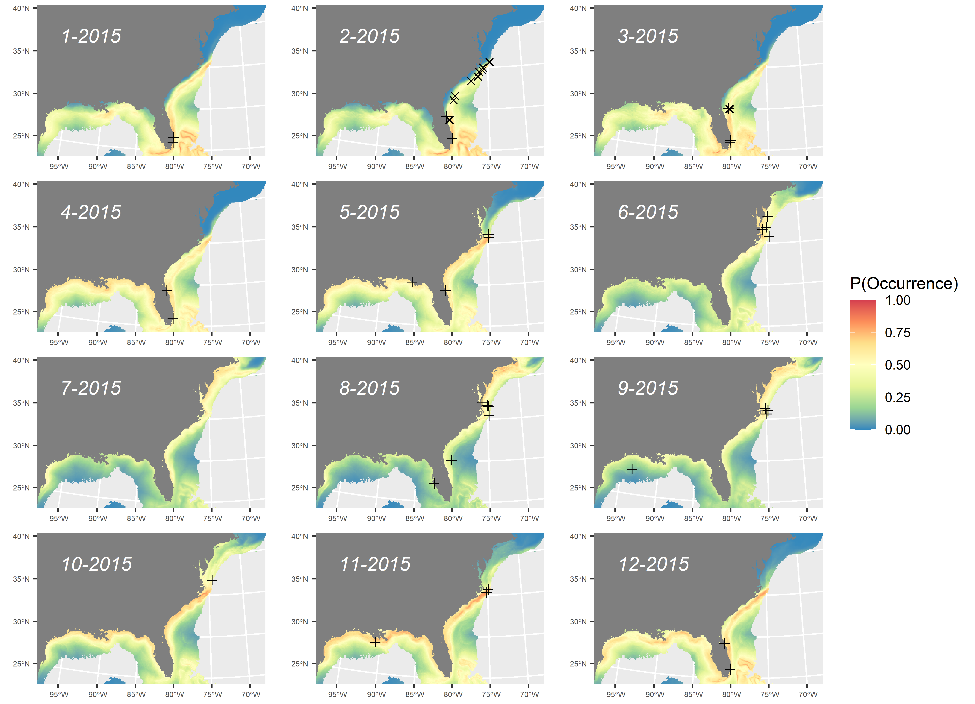

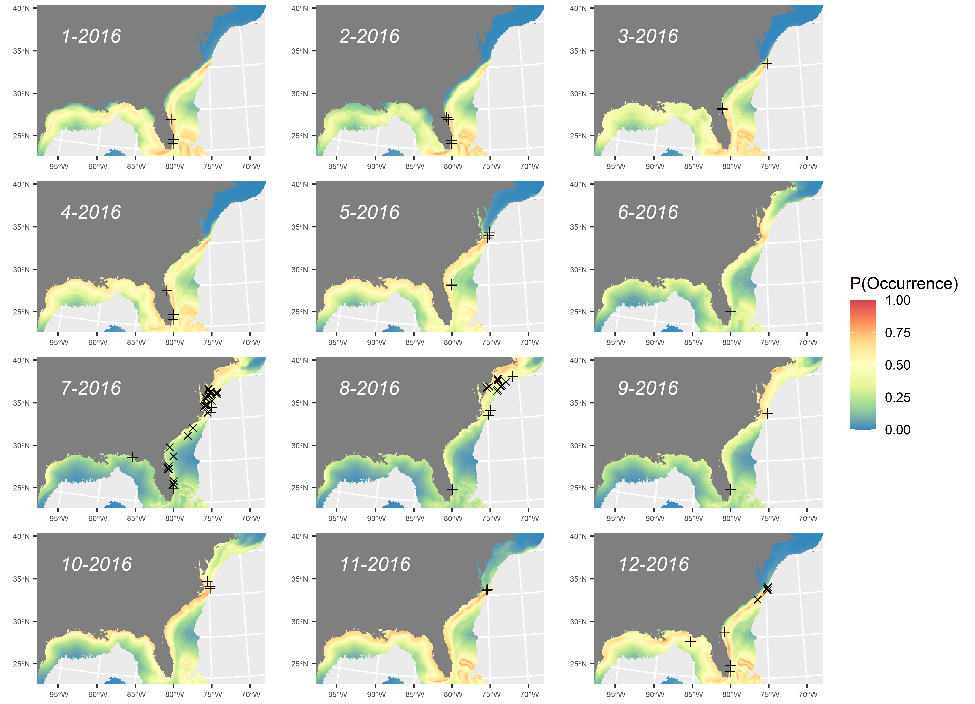

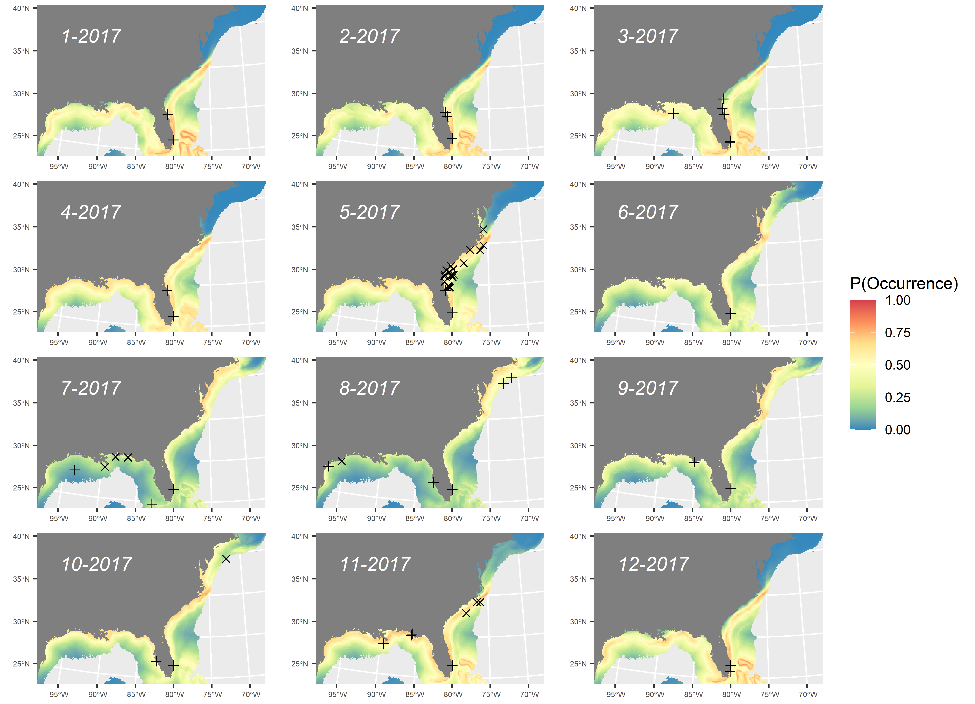

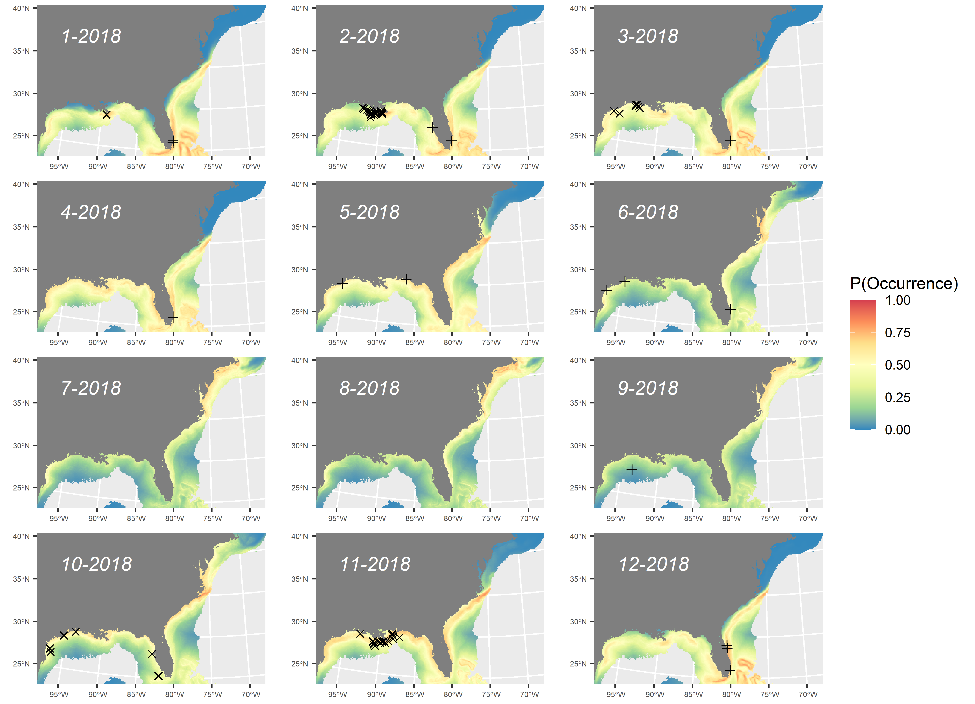

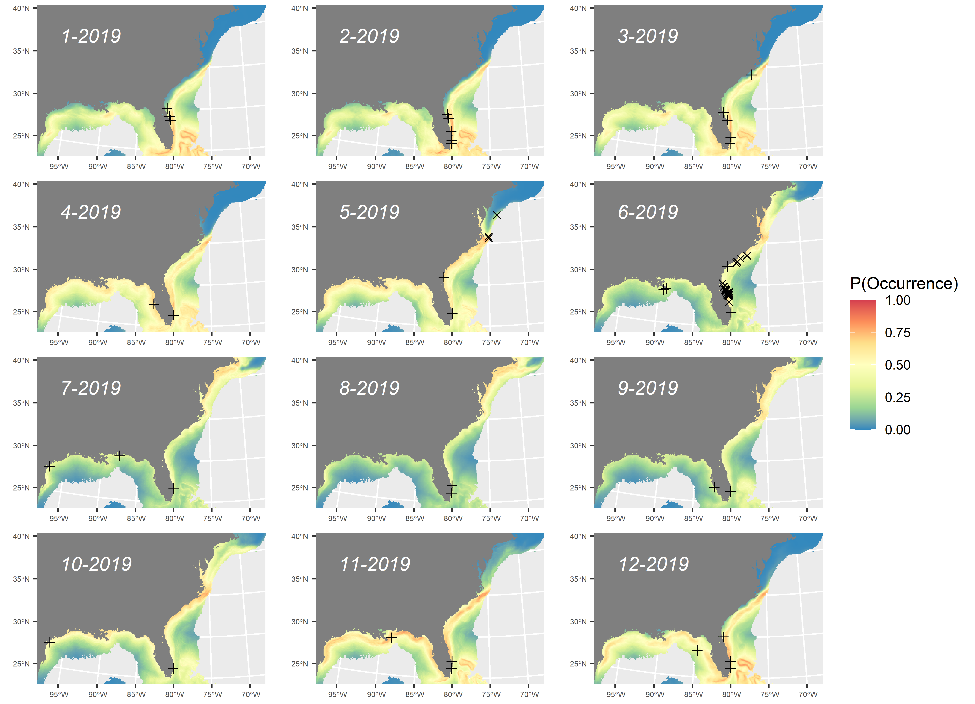


**Figure S13.** Time series of predicted probability of occurrence for manta rays for combined surveys (SEFSC, NARWC, and NYSERDA) weighted ensemble model for monthly average environmental conditions 2003 to 2019 with overlay of internal (X) and external (+) validation points. Map generated in R v4.1.2 (https://cran.r-project.org/bin/windows/base/).
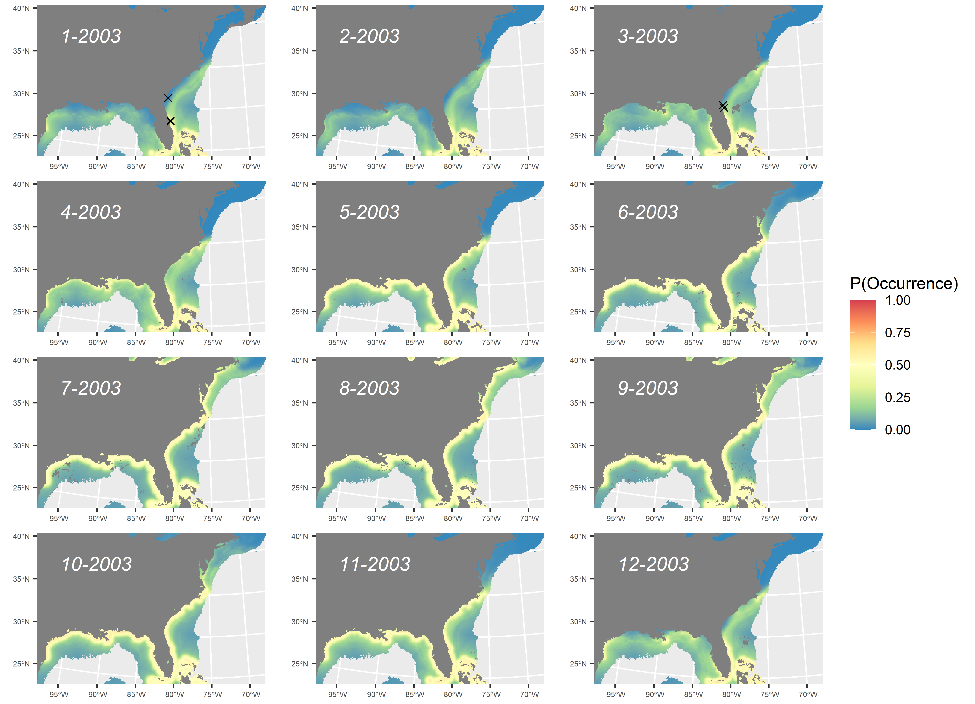

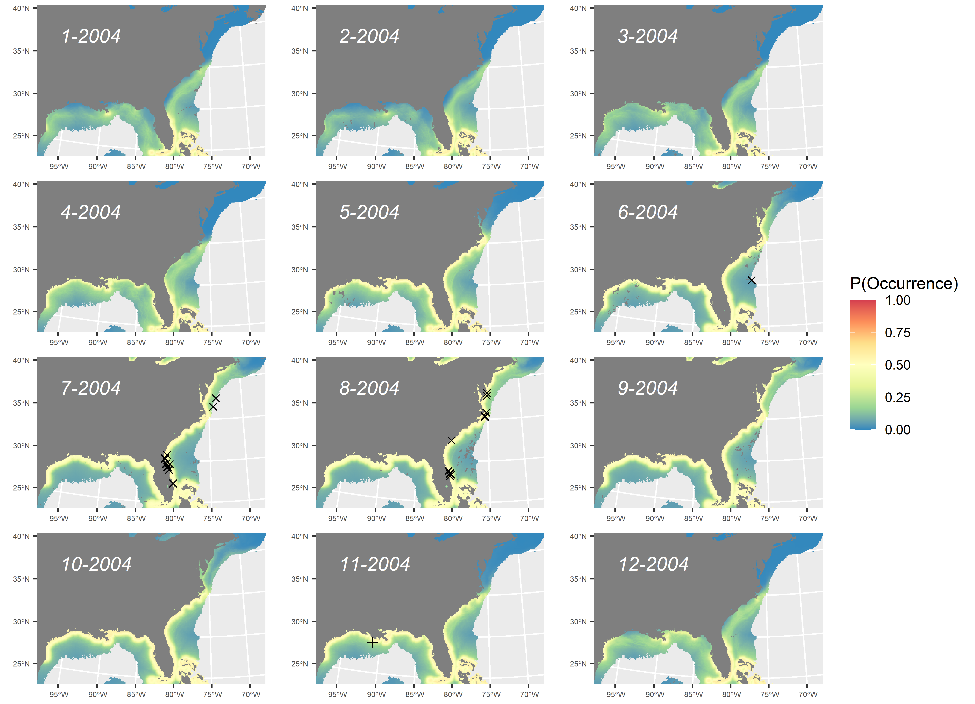

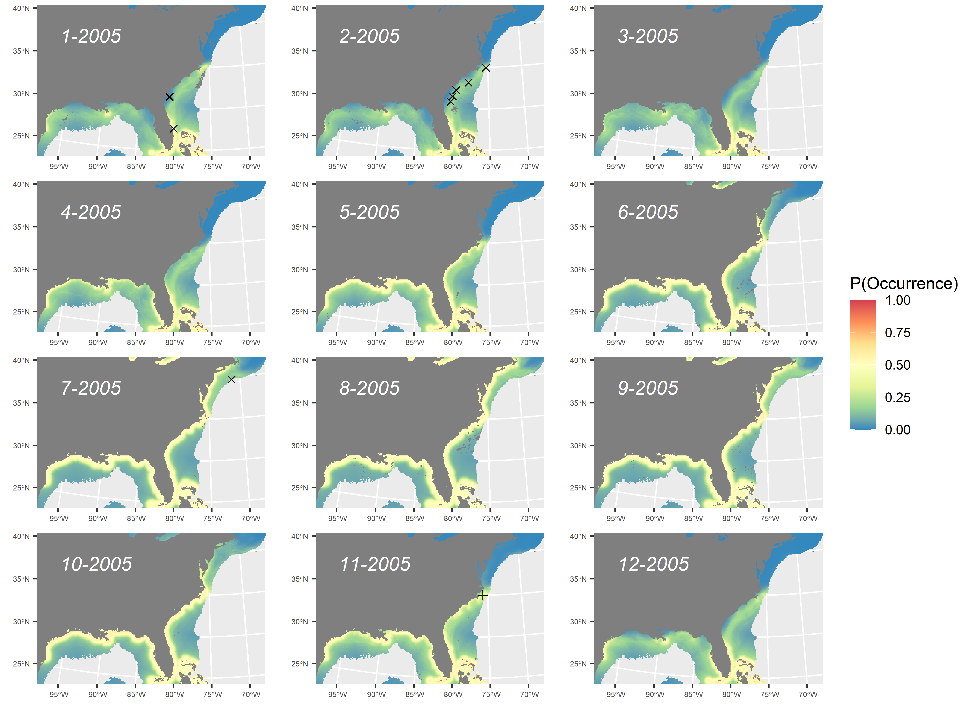

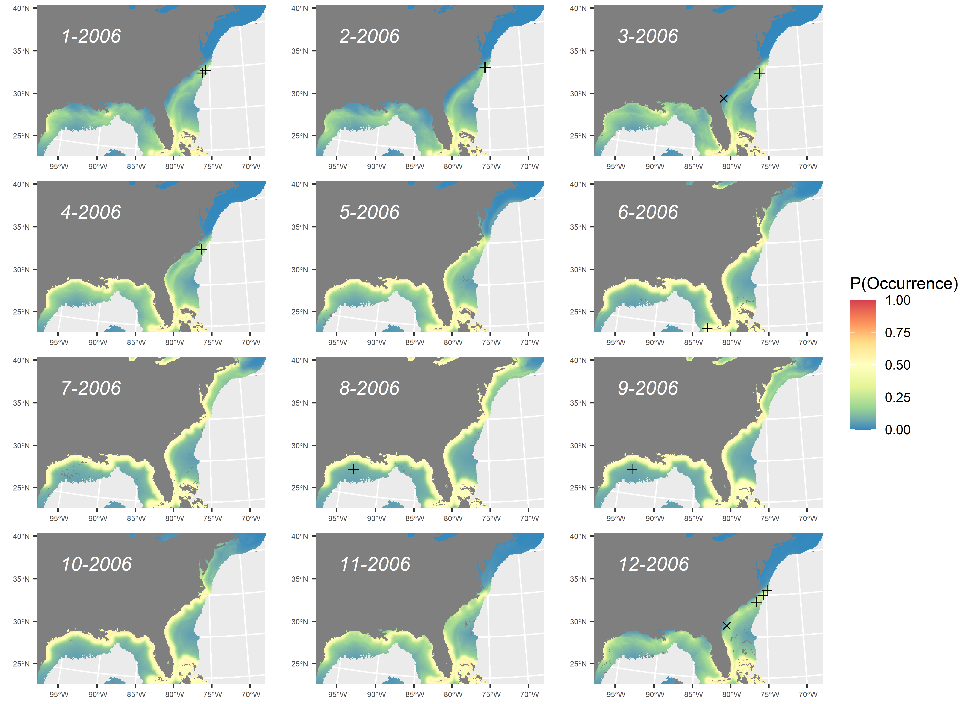

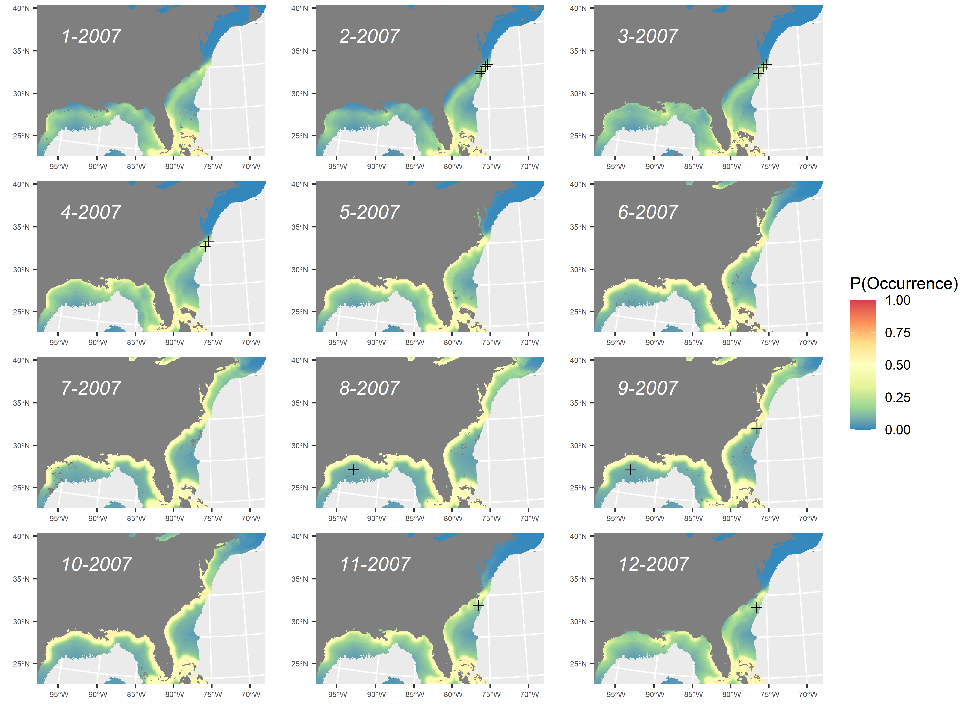

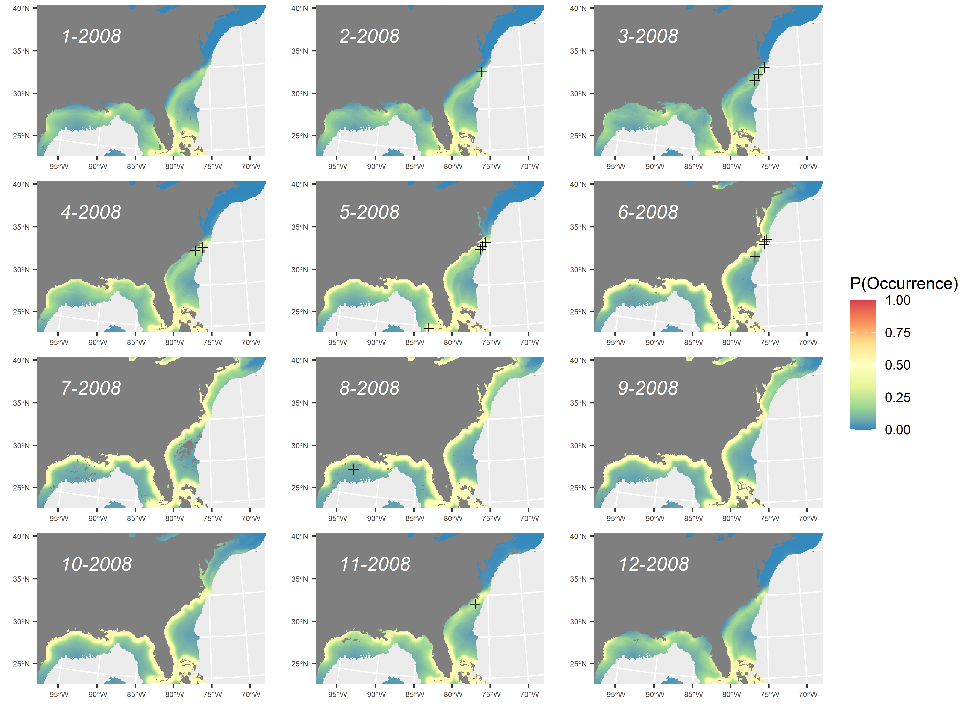

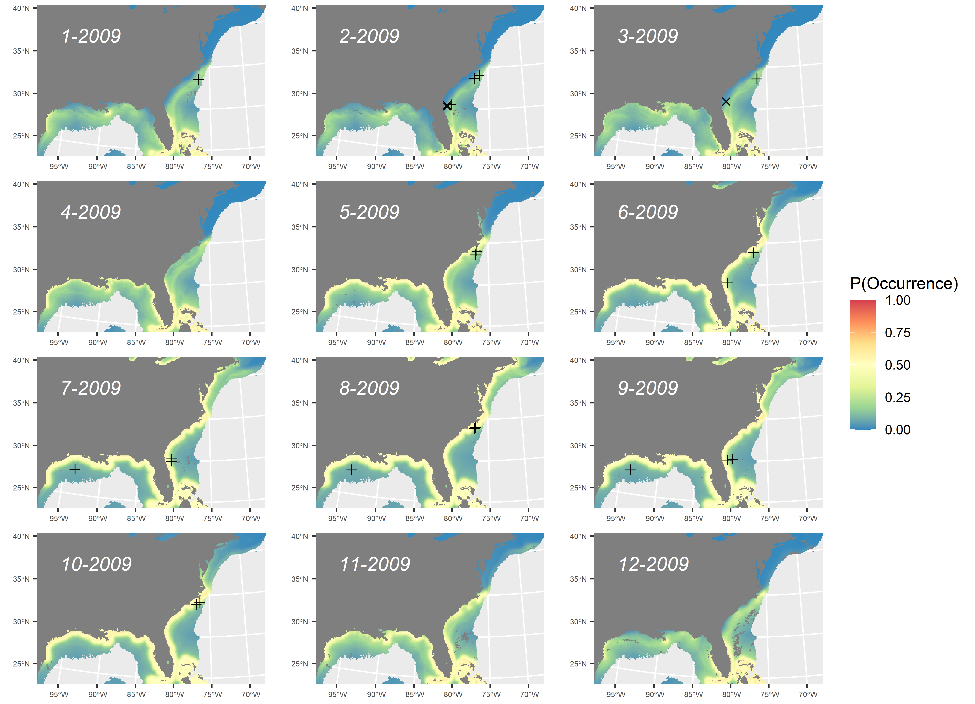

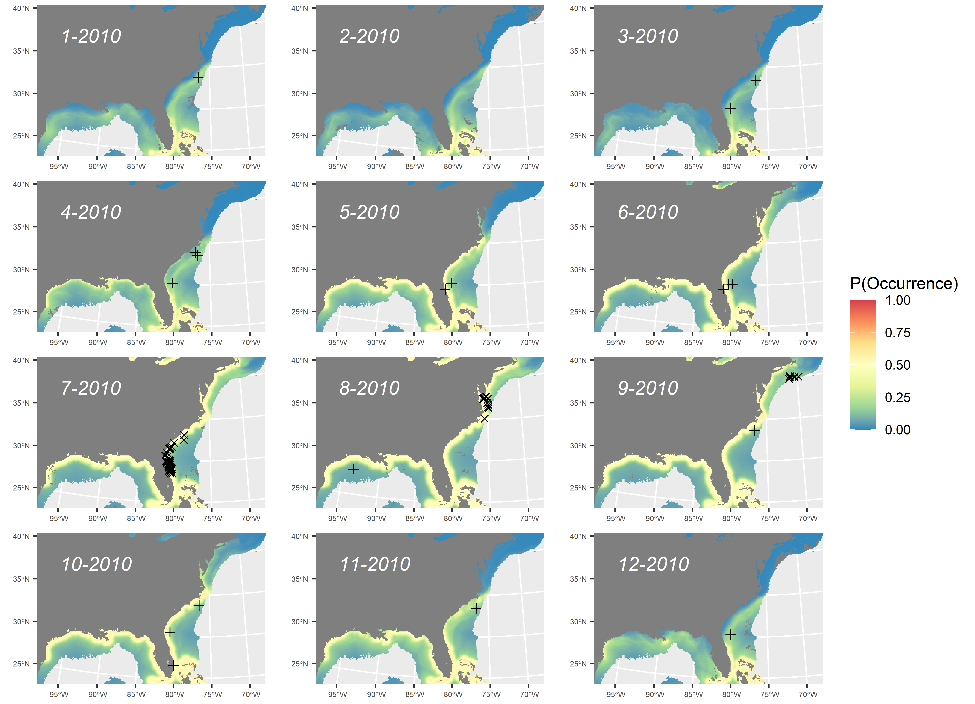

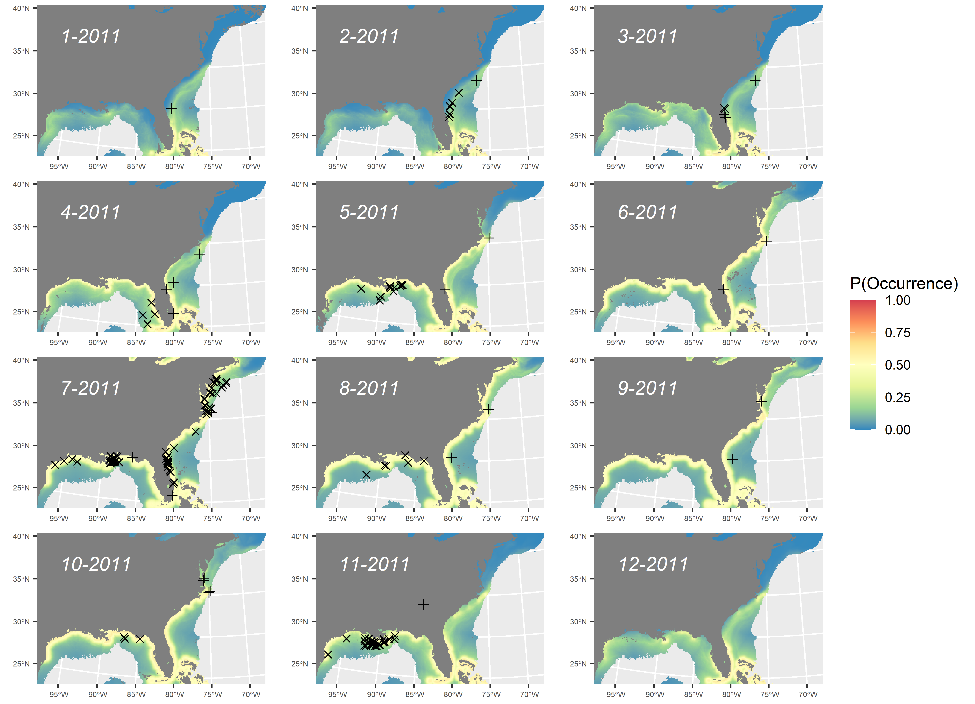

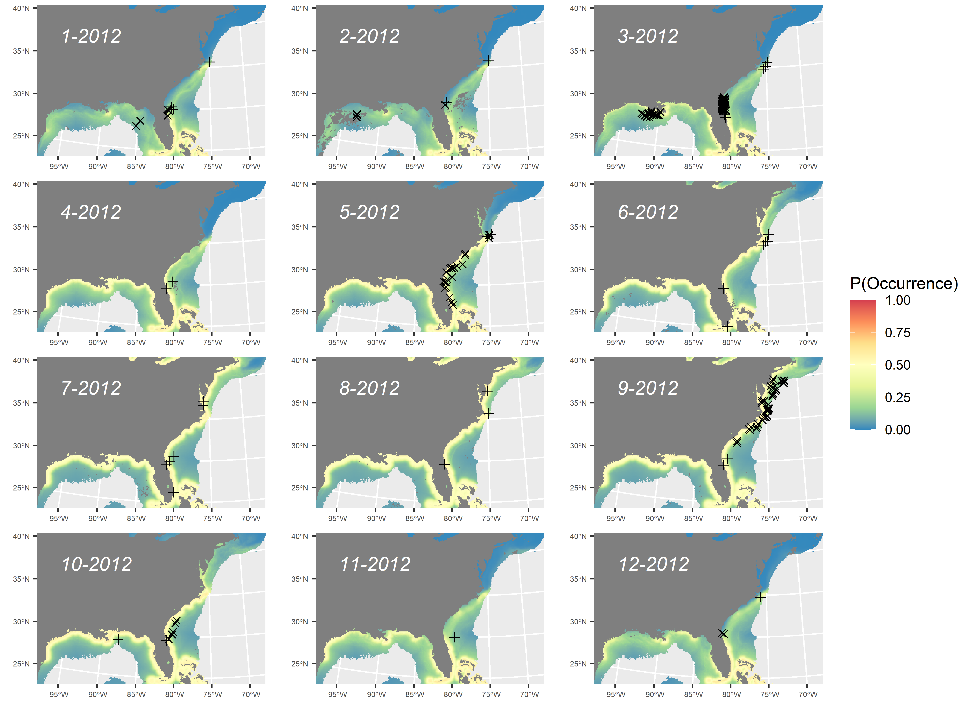

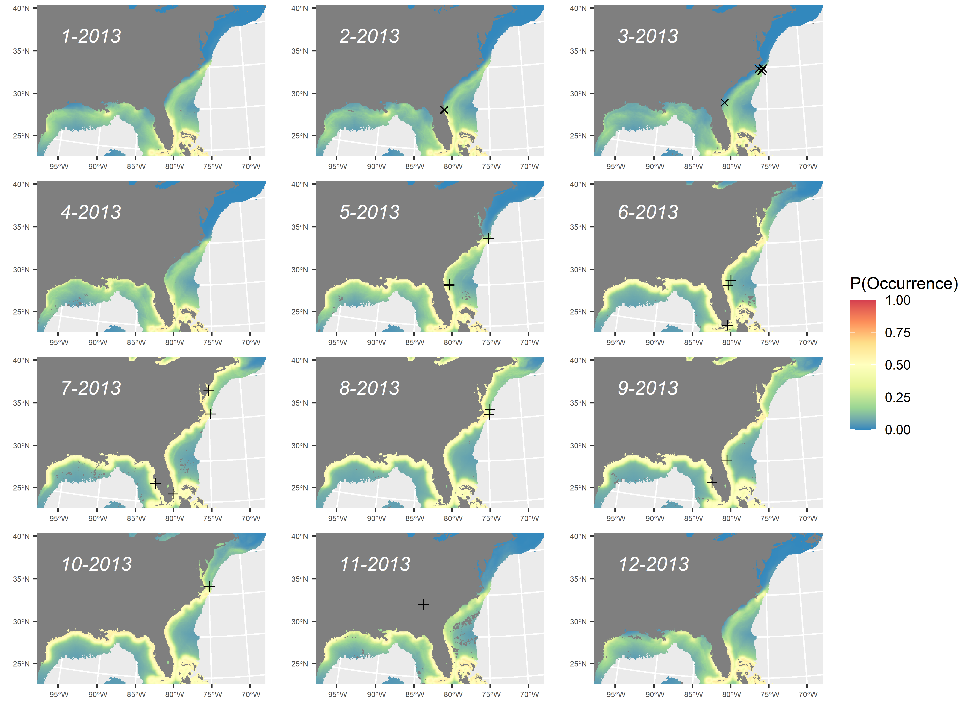

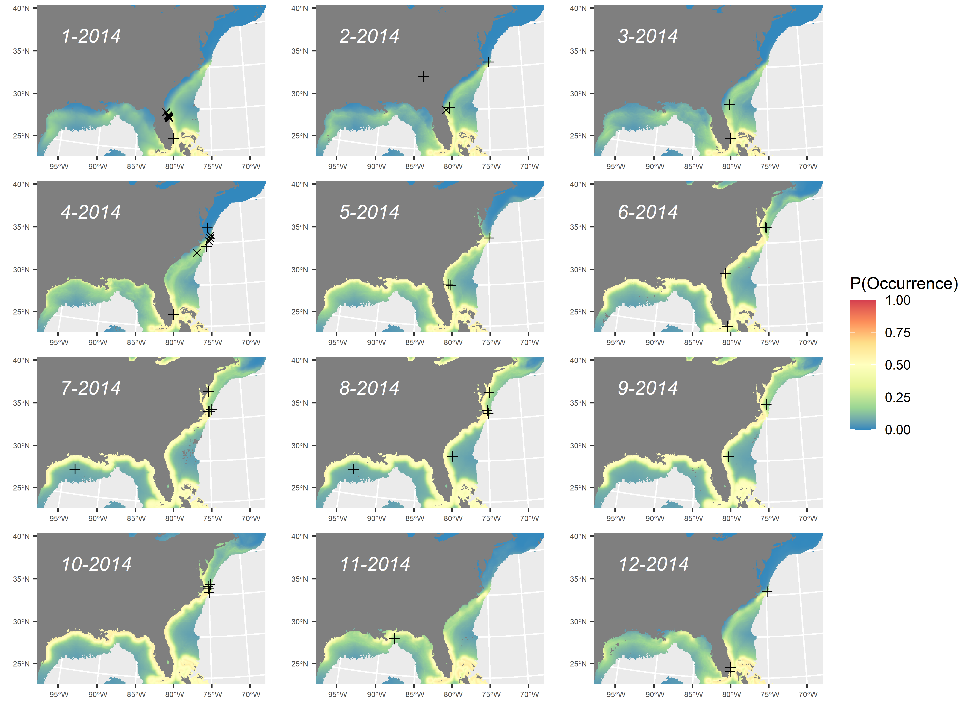

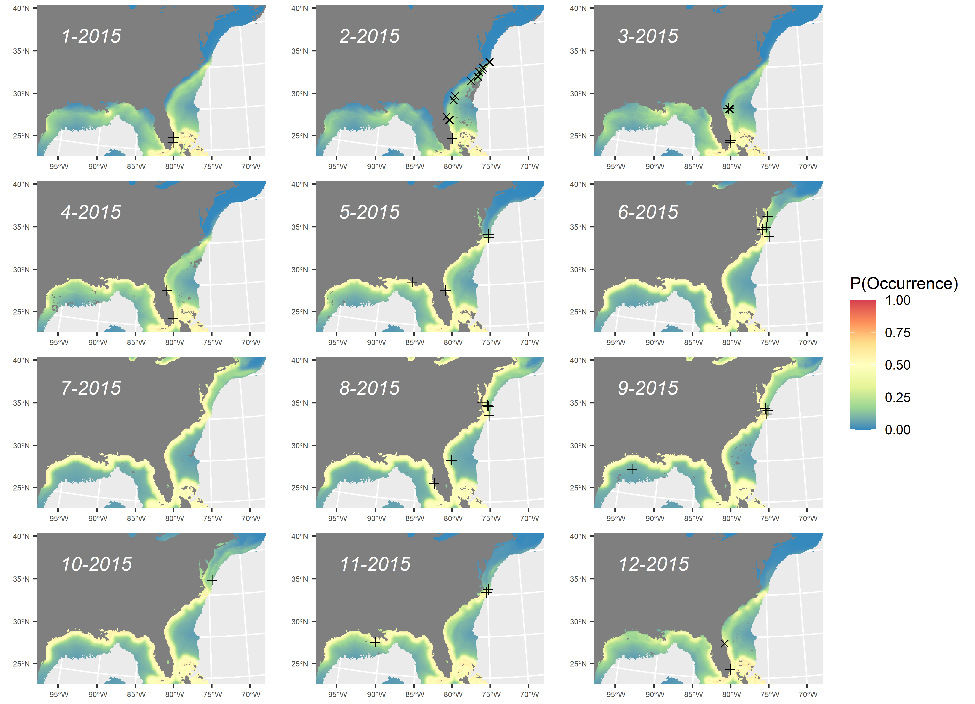

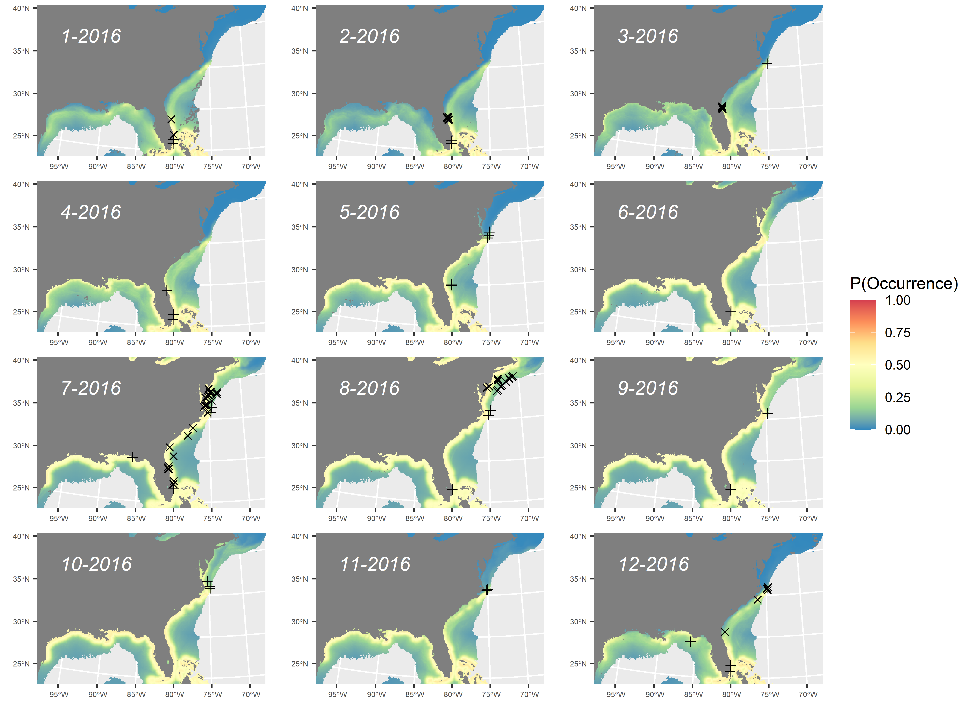

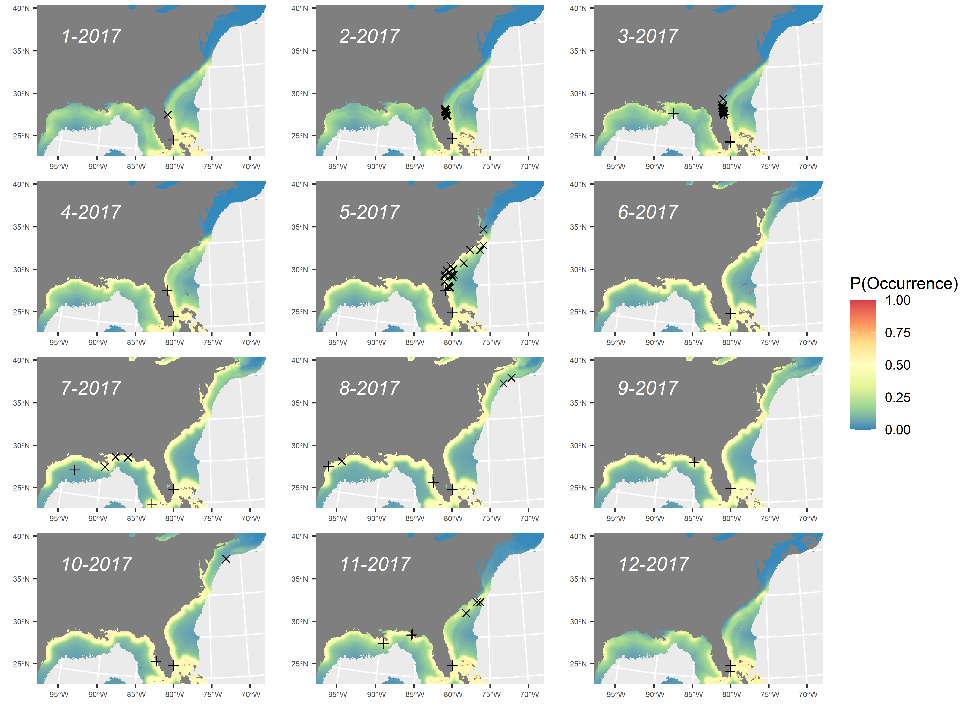

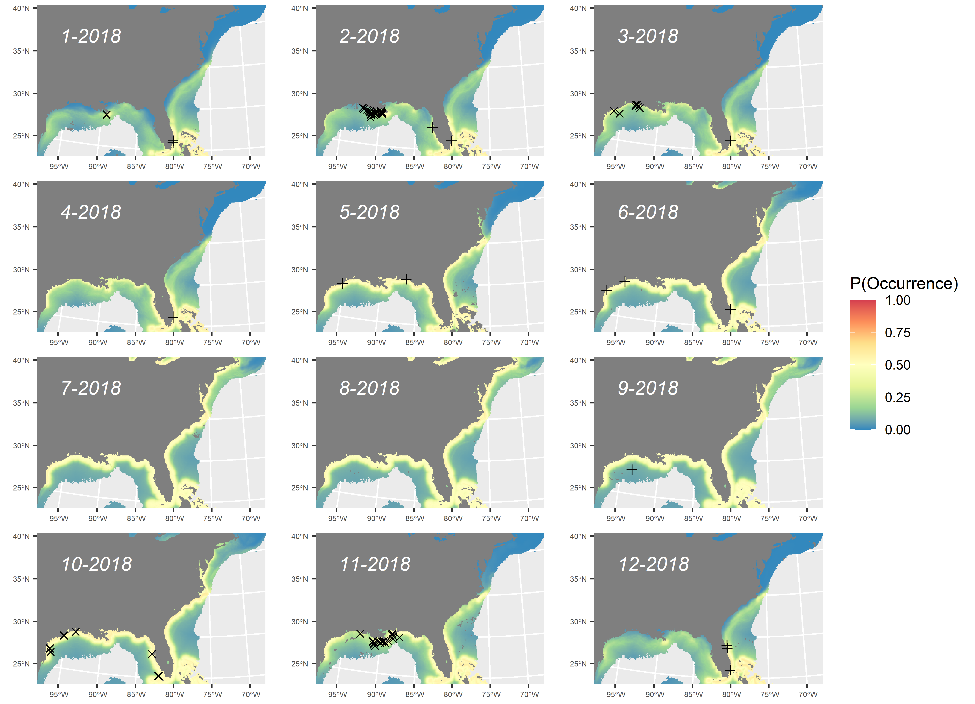

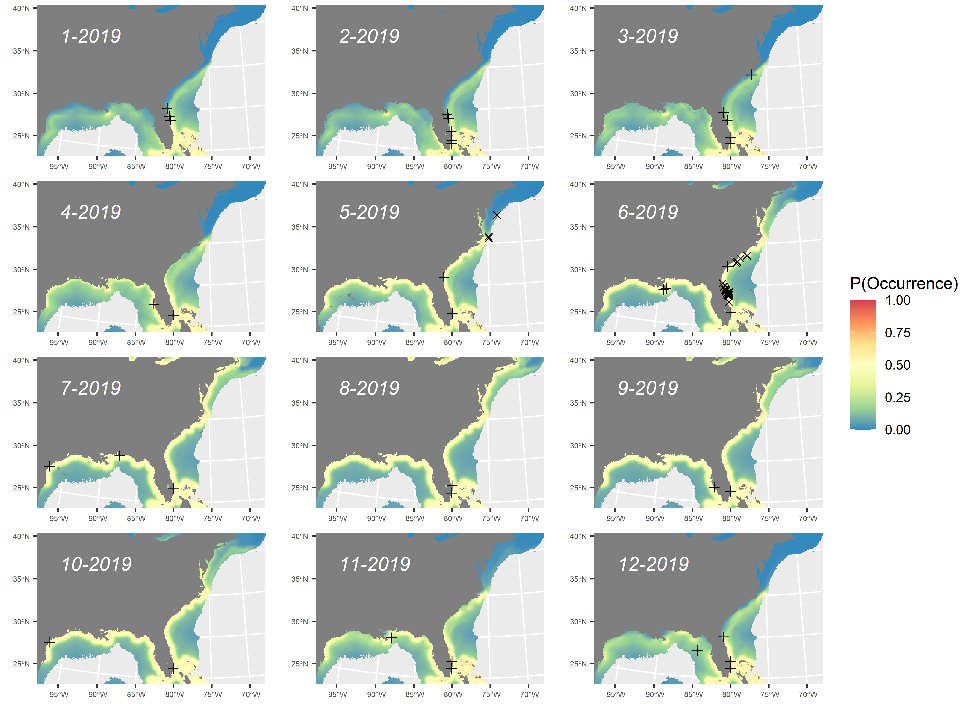


**Figure S14.** Time series of predicted probability of occurrence for manta rays for combined surveys (SEFSC, NARWC, and NYSERDA) model for monthly average environmental conditions 2003 to 2019 with overlay of internal (X) and external (+) validation points. Map generated in R v4.1.2 (https://cran.r-project.org/bin/windows/base/).
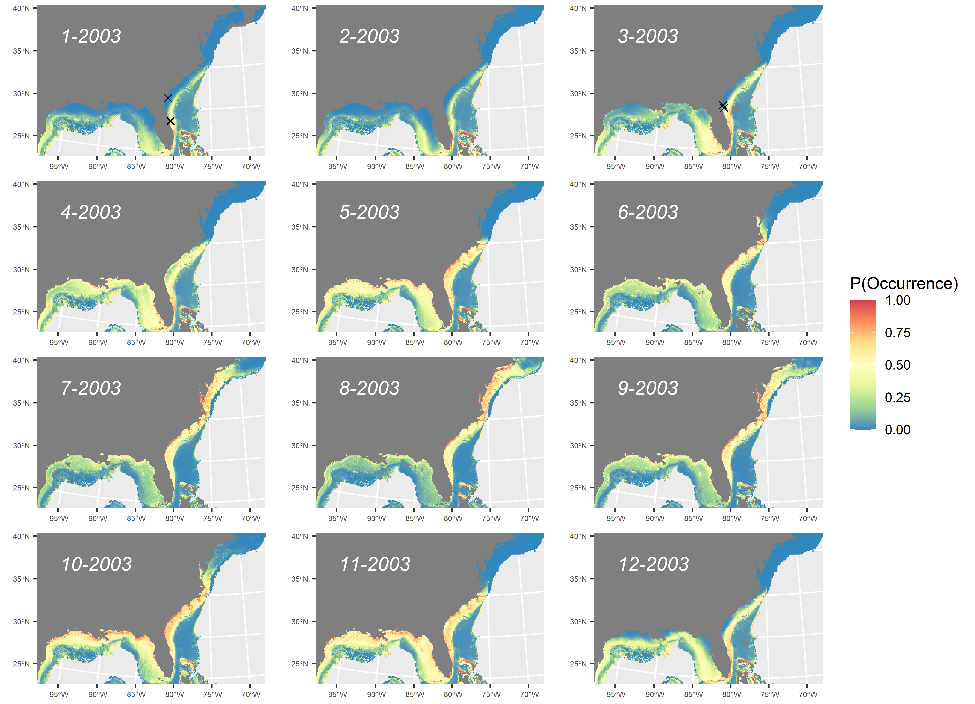

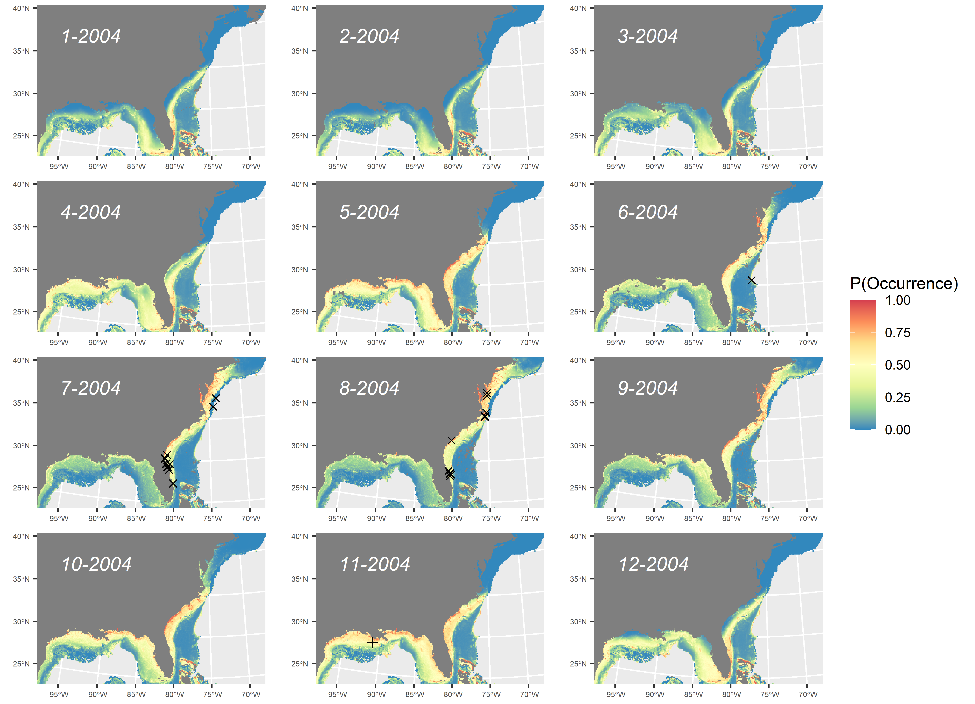

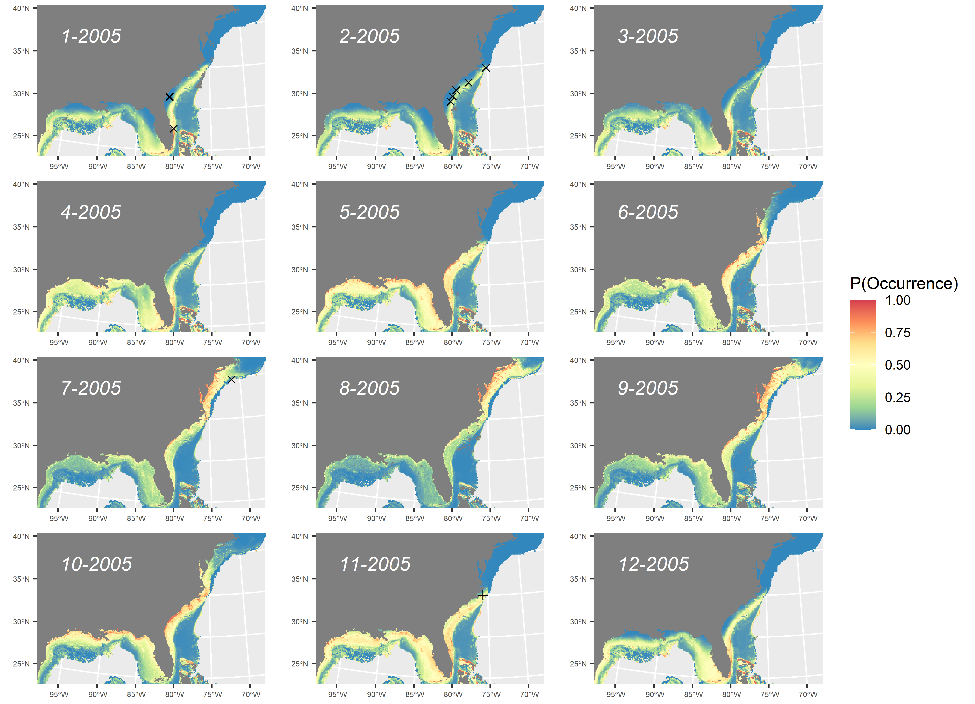

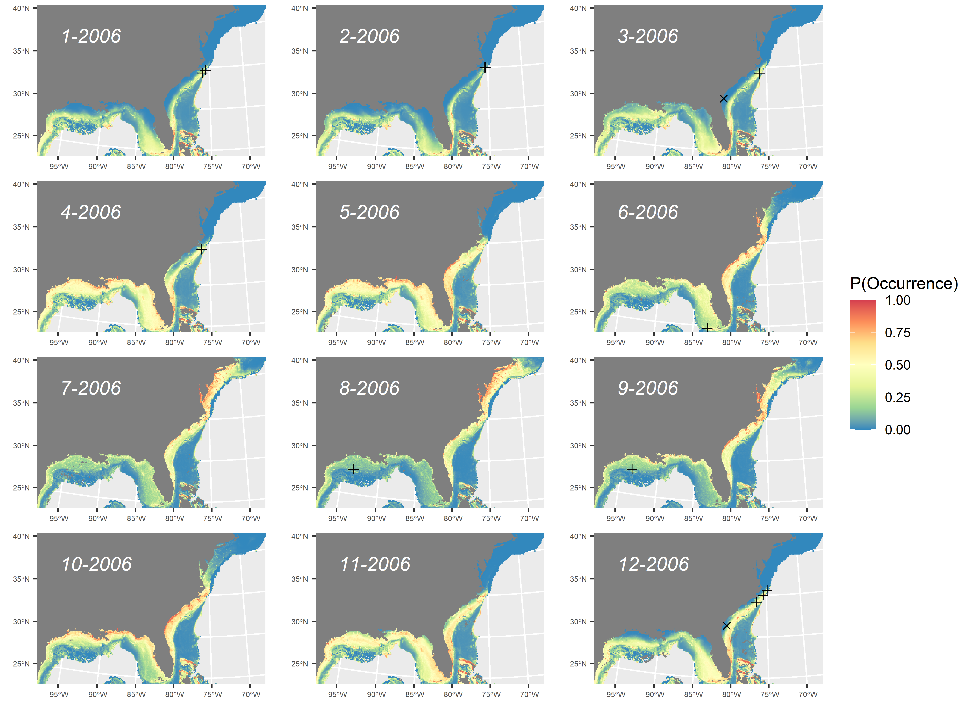

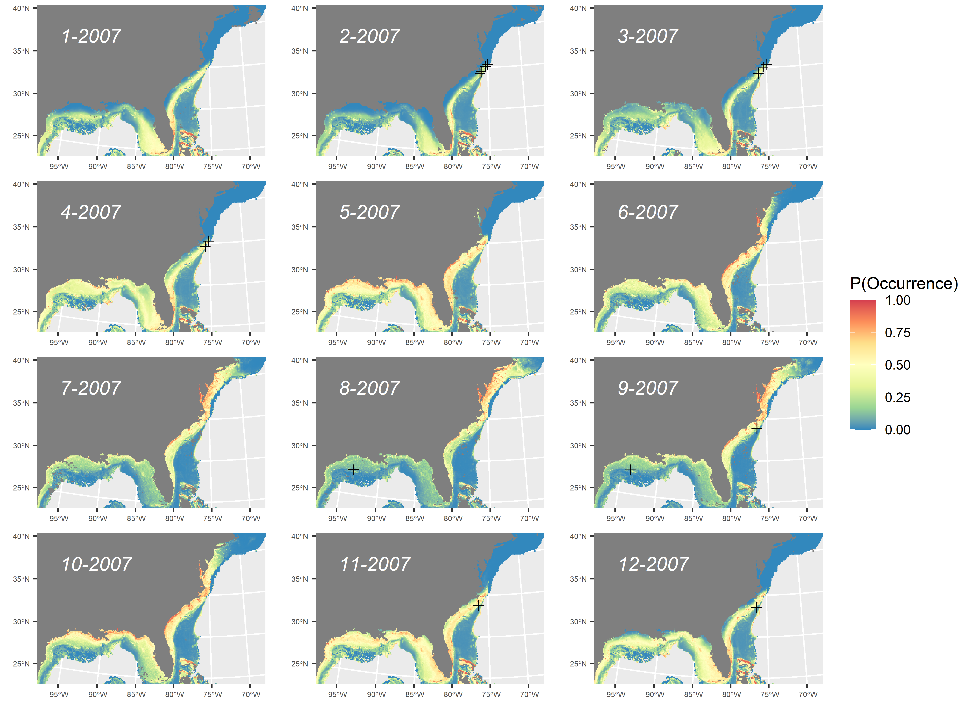

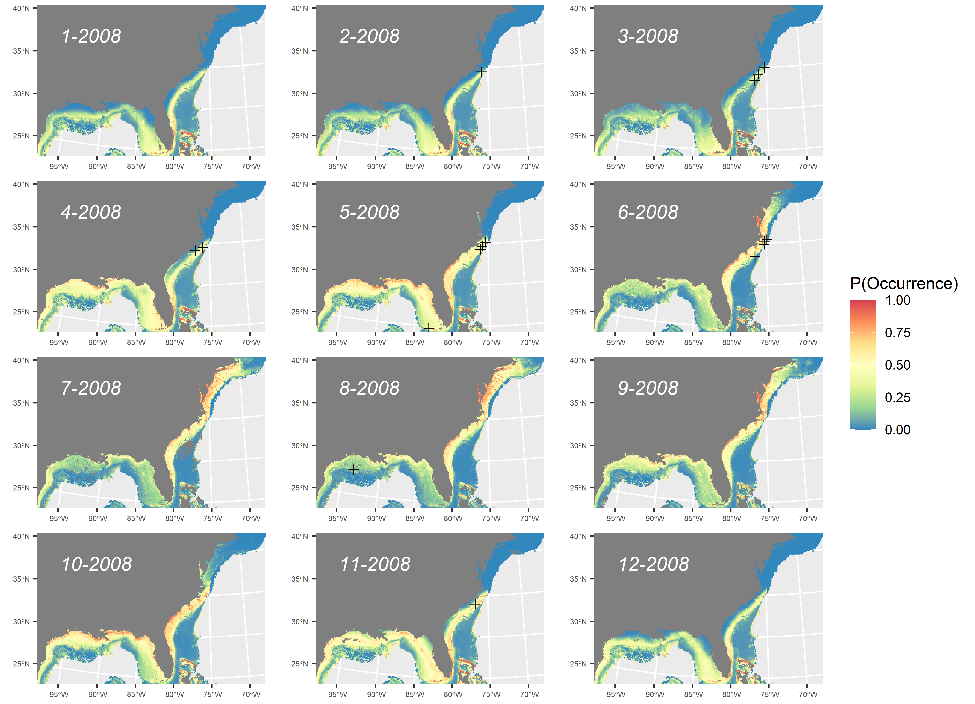

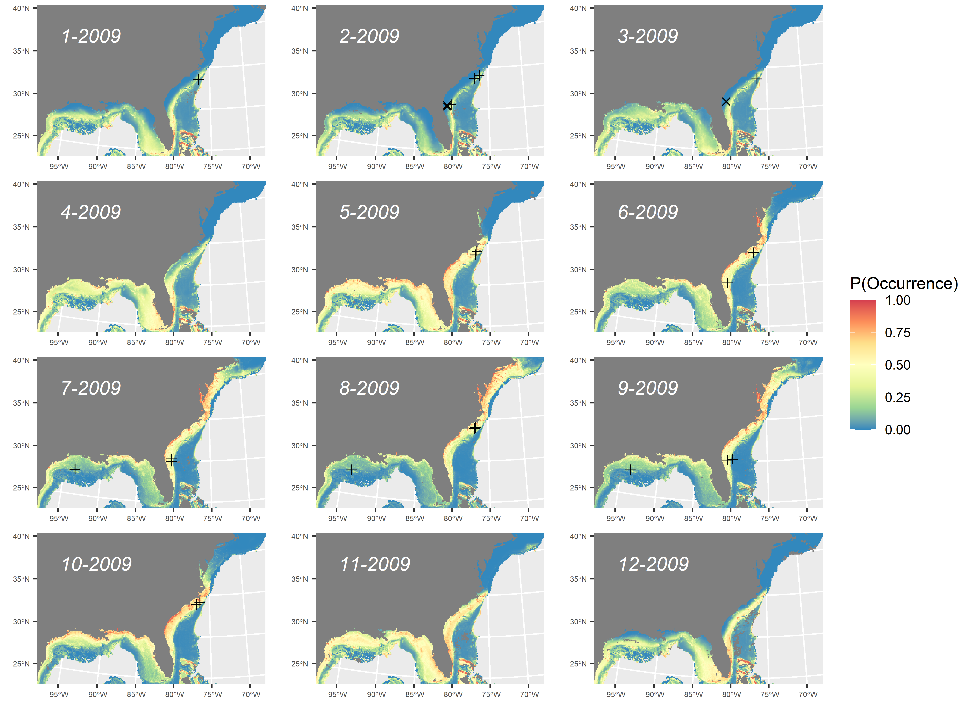

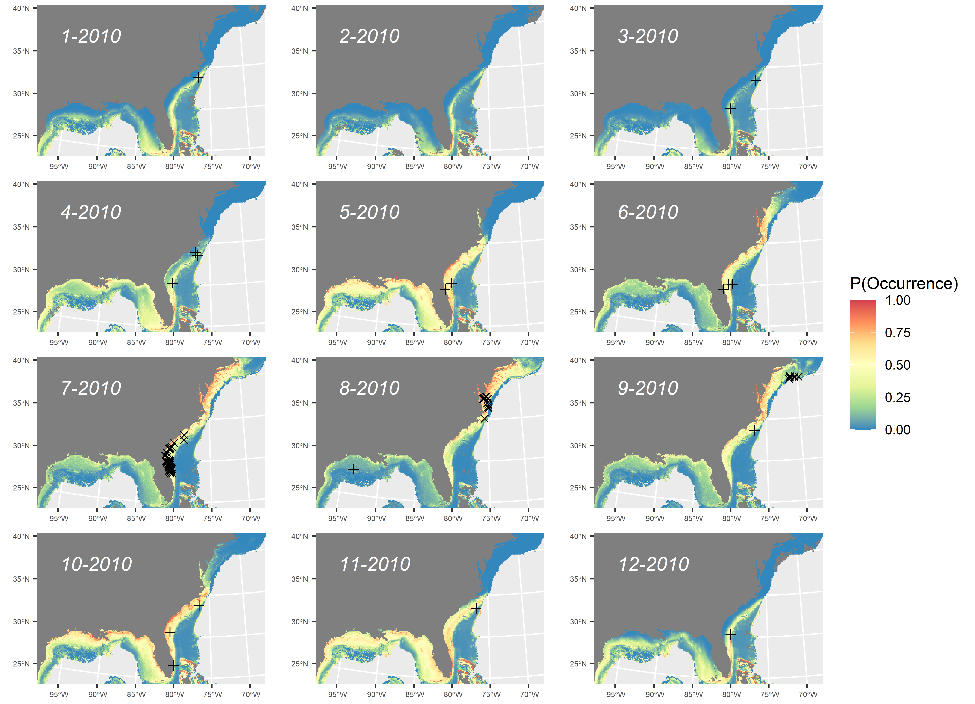

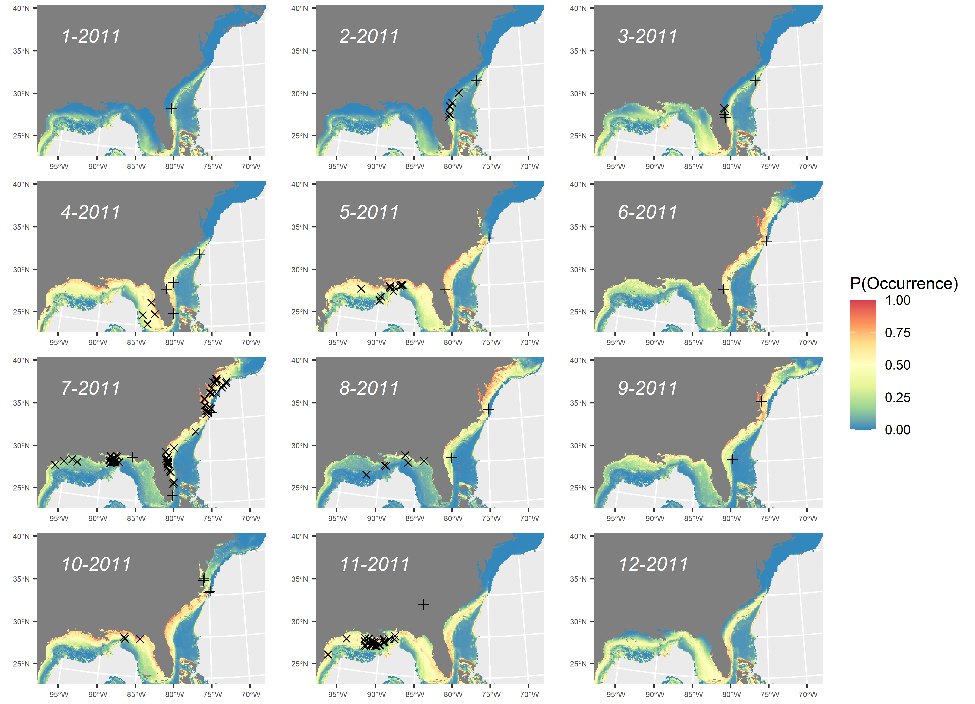

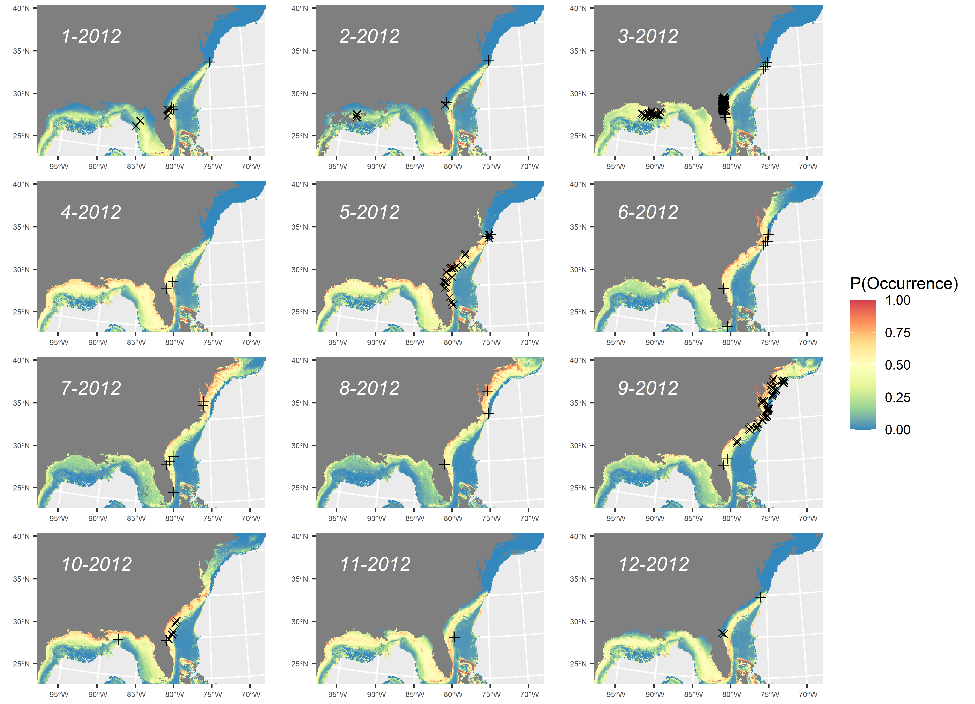

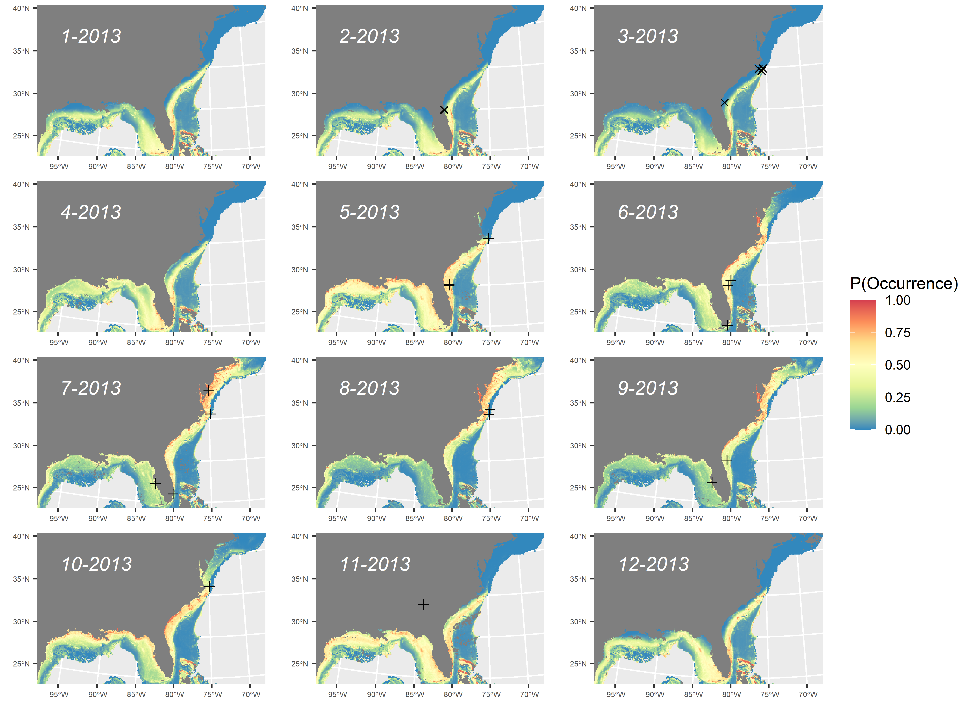

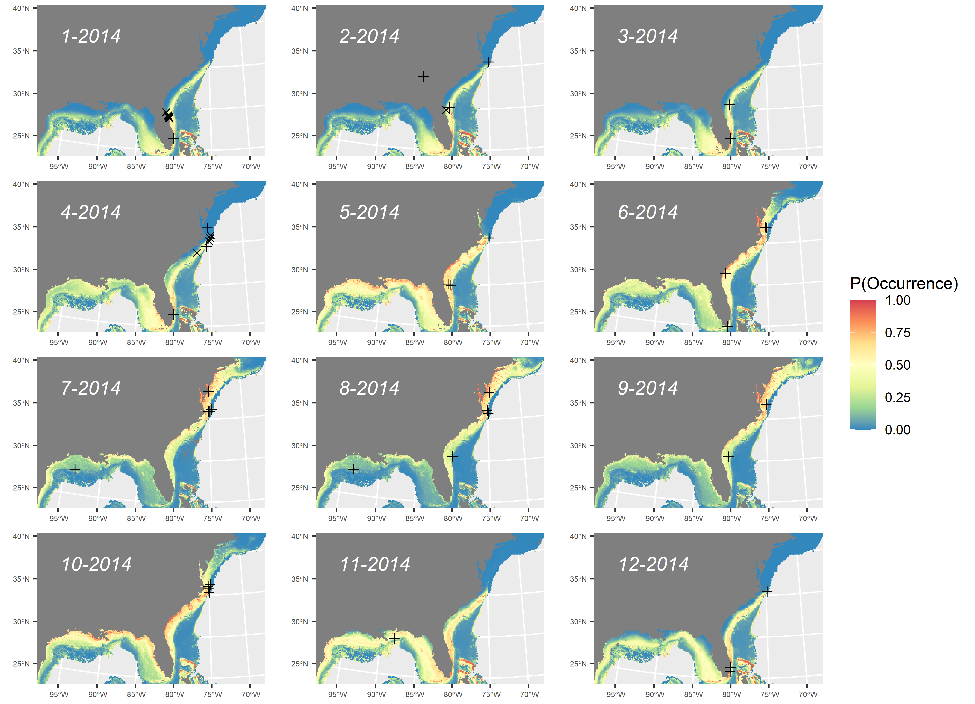

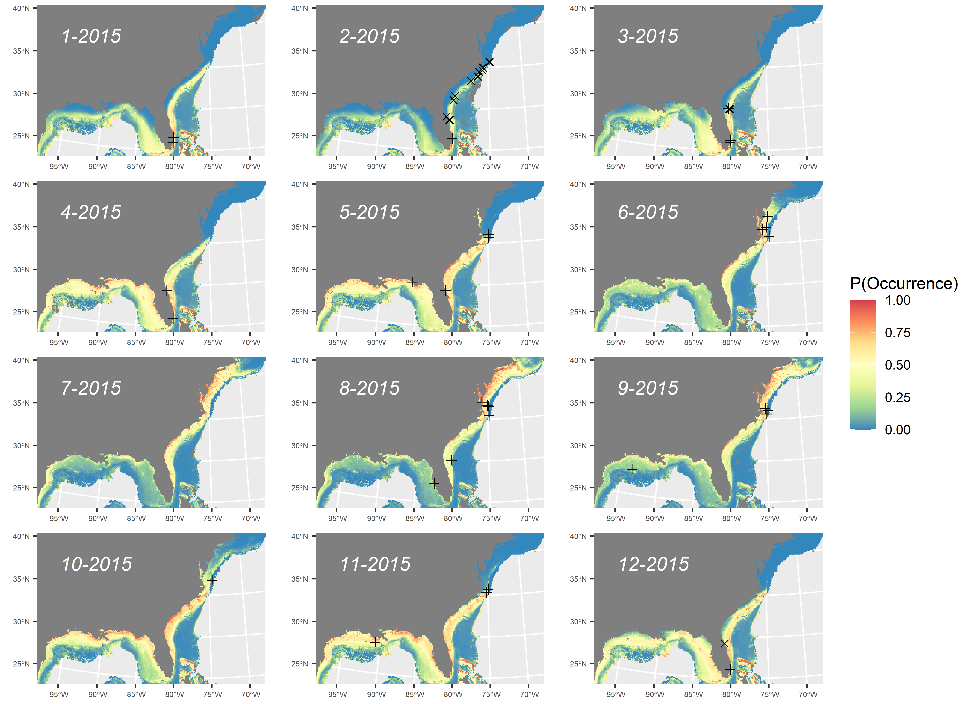

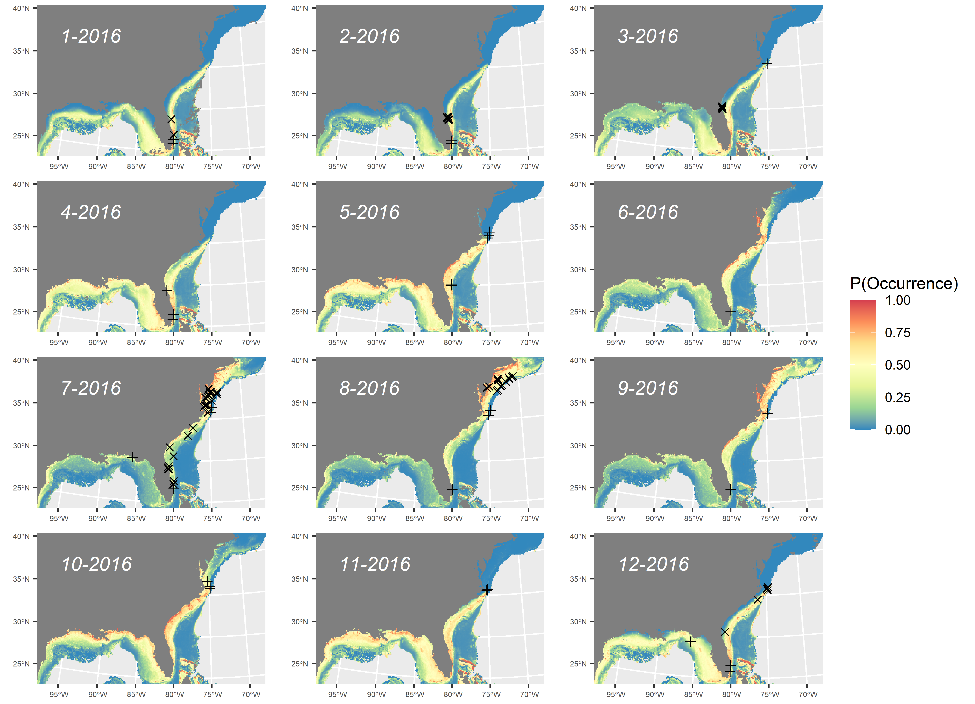

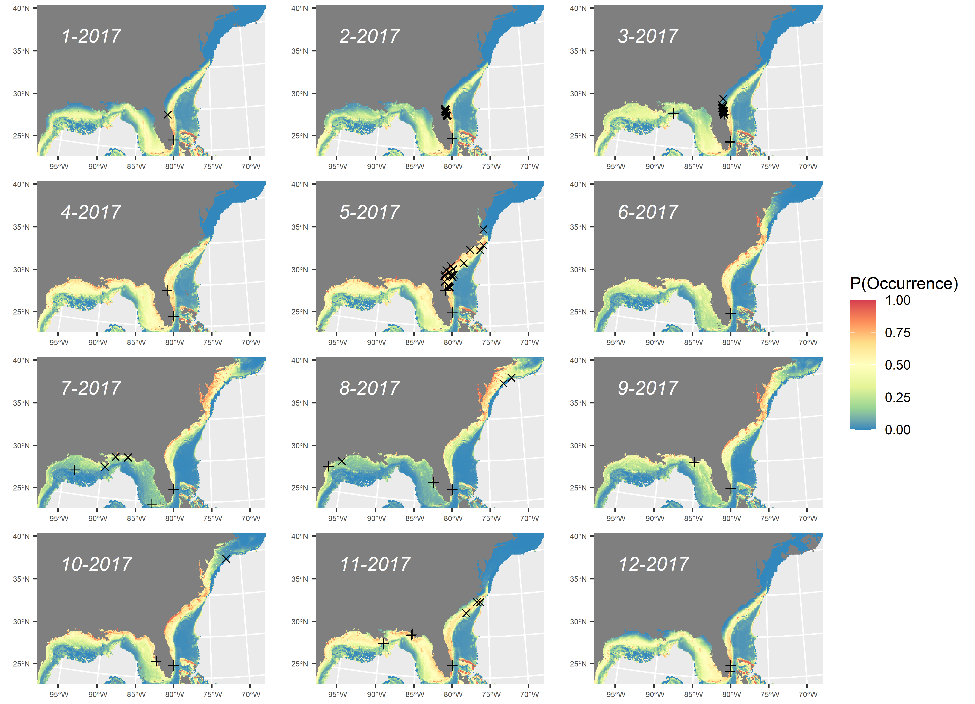

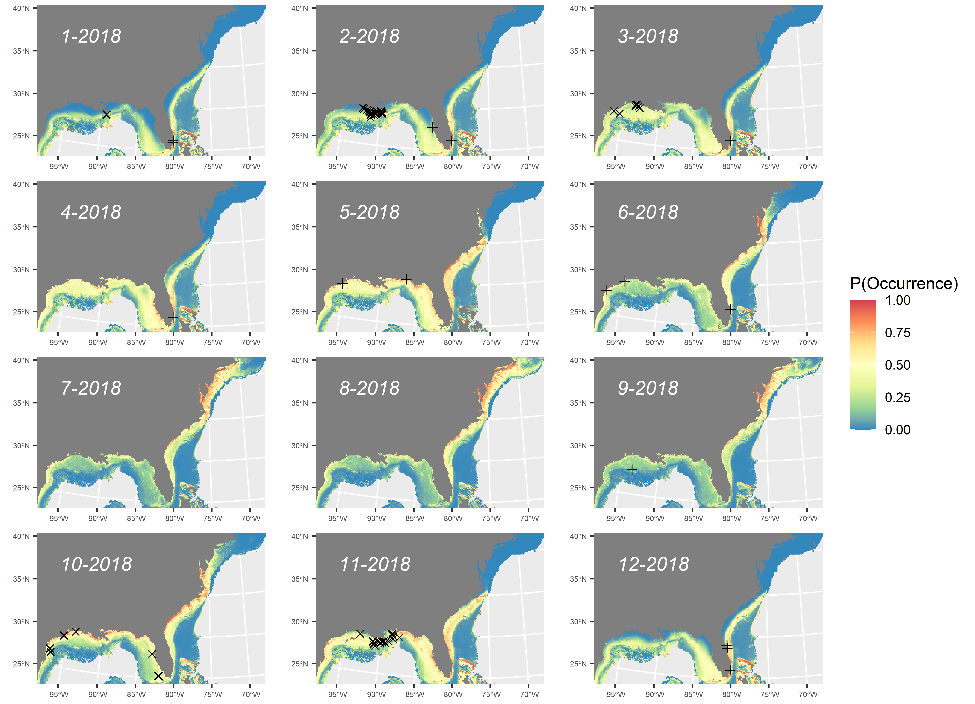

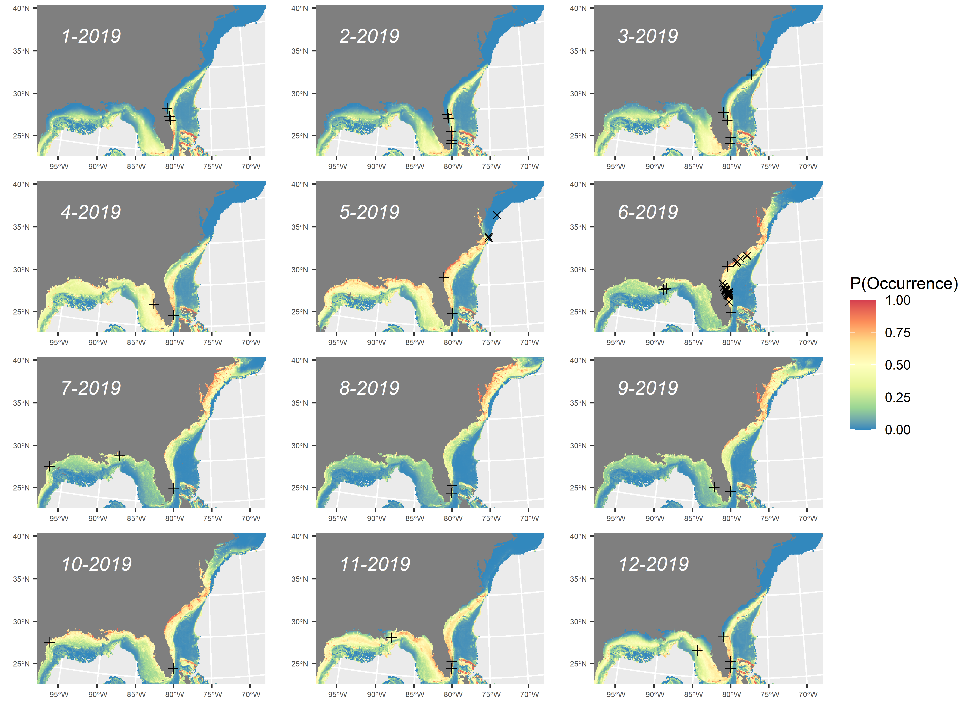


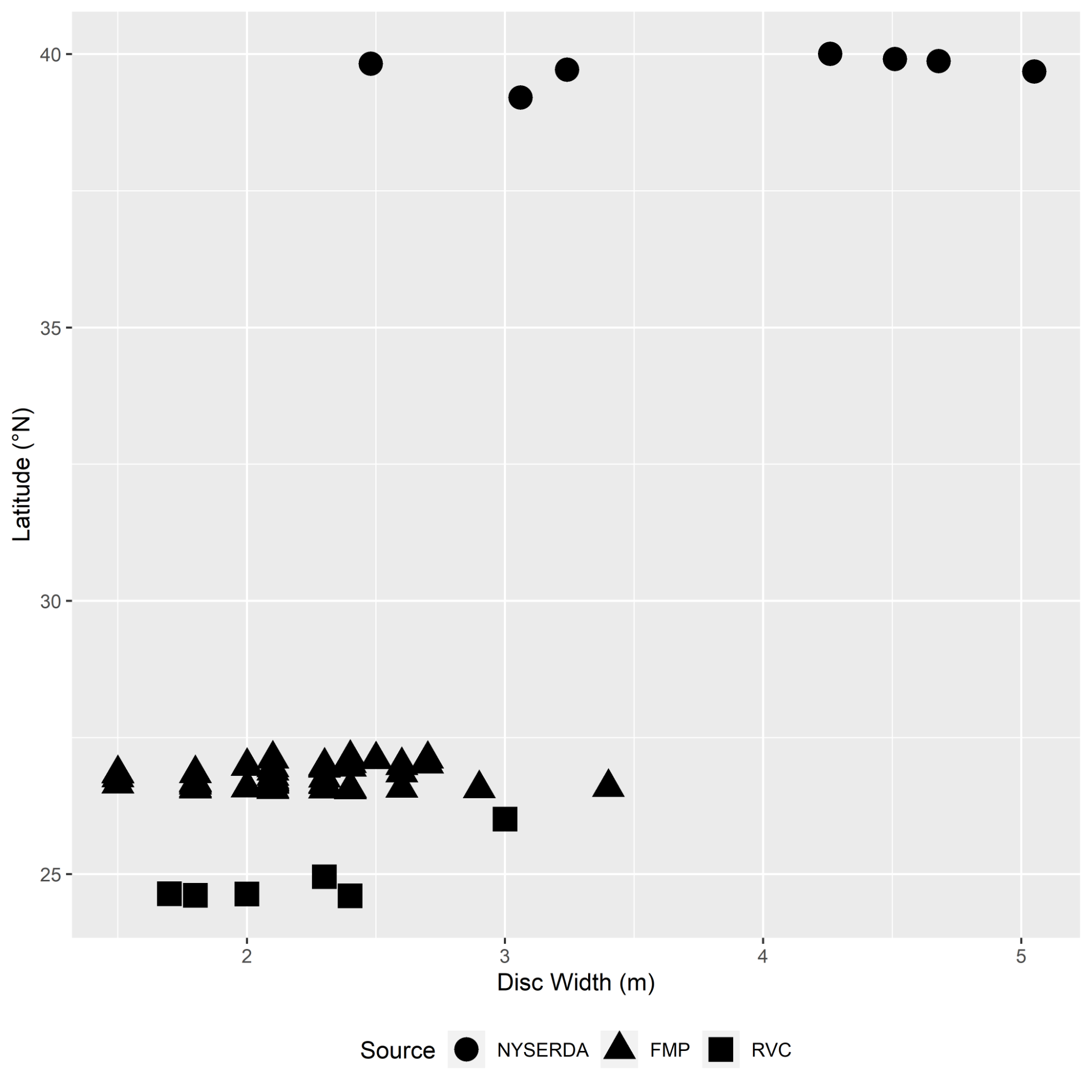


**Figure S15.** Estimated disc width (m) for manta rays observed by National Marine Fisheries Service Reef Visual Census (RVC) divers, Florida Manta Project (FMP) snorkelers, and NYSERDA aerial digital photographic survey, by latitude.


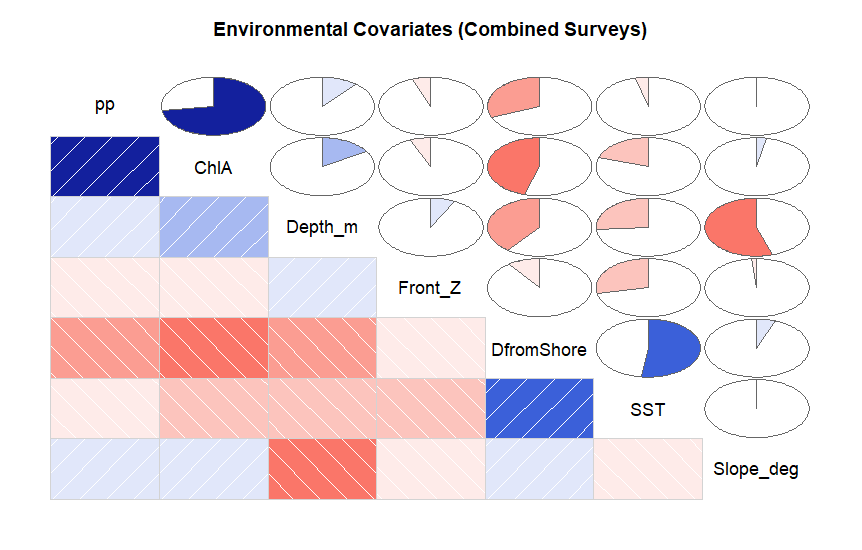


**Figure S16.** Correlogram of environmental covariates considered in species distribution modeling framework, including primary productivity (pp), chlorophyll-a (ChlA), bathymetric depth in meters (Depth_m), daily standardized thermal frontal gradients (Front_Z), distance from shore (DfromShore), sea surface temperature (SST), and bathymetric slope in degrees (Slope_deg). See Methods for more details on sources and resolution for these covariates. Map generated in R v4.1.2 (https://cran.r-project.org/bin/windows/base/).
